# π-Extended ligands with dual-binding behavior: hindered rotation unlocks unexpected reactivity in cyclometalated Pt complexes†

**DOI:** 10.1039/d4sc04799k

**Published:** 2024-09-11

**Authors:** Seiya Ota, Miguel A. Soto, Brian O. Patrick, Saeid Kamal, Francesco Lelj, Mark J. MacLachlan

**Affiliations:** a Department of Chemistry University of British Columbia 2036 Main Mall Vancouver V6T 1Z1 Canada msoto@chem.ubc.ca mmaclach@chem.ubc.ca; b La.M.I.and LaSCAMM INSTM Sezione Basilicata, Dipartiento di Scienze, Università della Basilicata via dell'Ateneo Lucano 10 Potenza 85100 Italy; c Stewart Blusson Quantum Matter Institute University of British Columbia 2355 East Mall Vancouver BC V6T 1Z4 Canada; d WPI Nano Life Science Institute Kanazawa University Kanazawa 920-1192 Japan

## Abstract

Cyclometalated platinum complexes play a crucial role in catalysis, bioimaging, and optoelectronics. Phenylpyridines are widespread cyclometalating ligands that generate stable and highly emissive Pt complexes. While it is common practice to modify these ligands to fine-tune their photophysical properties, the incorporation of polycyclic aromatic hydrocarbons into the ligand's structure has been largely overlooked. This report describes the cyclometalation of naphthalenyl- and anthracenylpyridine ligands, which has resulted in ten new luminescent Pt^II^ and Pt^IV^ complexes. These species are enabled by a dual-binding behavior discovered in our polyaromatic-containing ligands. The introduction of naphthalenyl and anthracenyl groups unlocks dual binding modes, with the Pt center bonding to either of two distant carbon atoms within the ligand. These complexes exhibit both symmetric structures with two 5-membered metallacycles and asymmetric structures with 5- and 6-membered metallacycles. This work presents a strategy for the regioselective synthesis of Pt complexes with bespoke structures and photophysical properties. Our findings offer new opportunities in platinum chemistry and beyond, with potential implications for materials and technologies.

## Abstract 2

Naphthalenyl- and anthracenylpyridine cyclometalating ligands bind to Pt in different ways, which can be used to tune their emissive properties.

## Graphical abstract

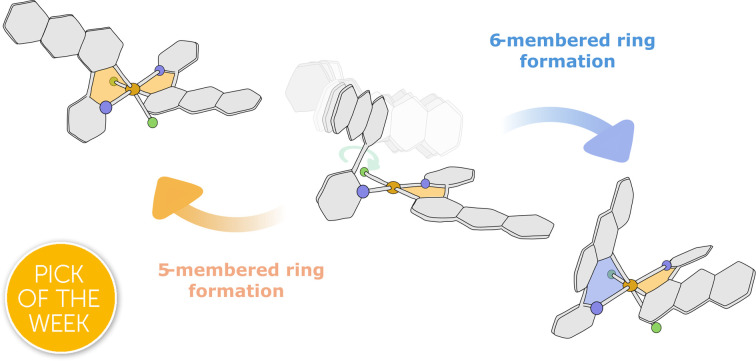

## Introduction

Cyclometalated platinum complexes have been the subject of extensive research in the last few decades due to their unique photophysical properties, reactivity, and stability.^1–4^ Many cyclometalating ligands have been reported in the literature, with arylpyridines^5–7^ and bis(aryl)pyridines^7,8^ being among the most studied. These structurally tailorable ligands are easy to access and once bound to Pt impart unique properties for various applications, including bioimaging,^9–11^ optoelectronics,^12,13^ sensing,^14,15^ and catalysis.^16,17^

**Fig. 1:**
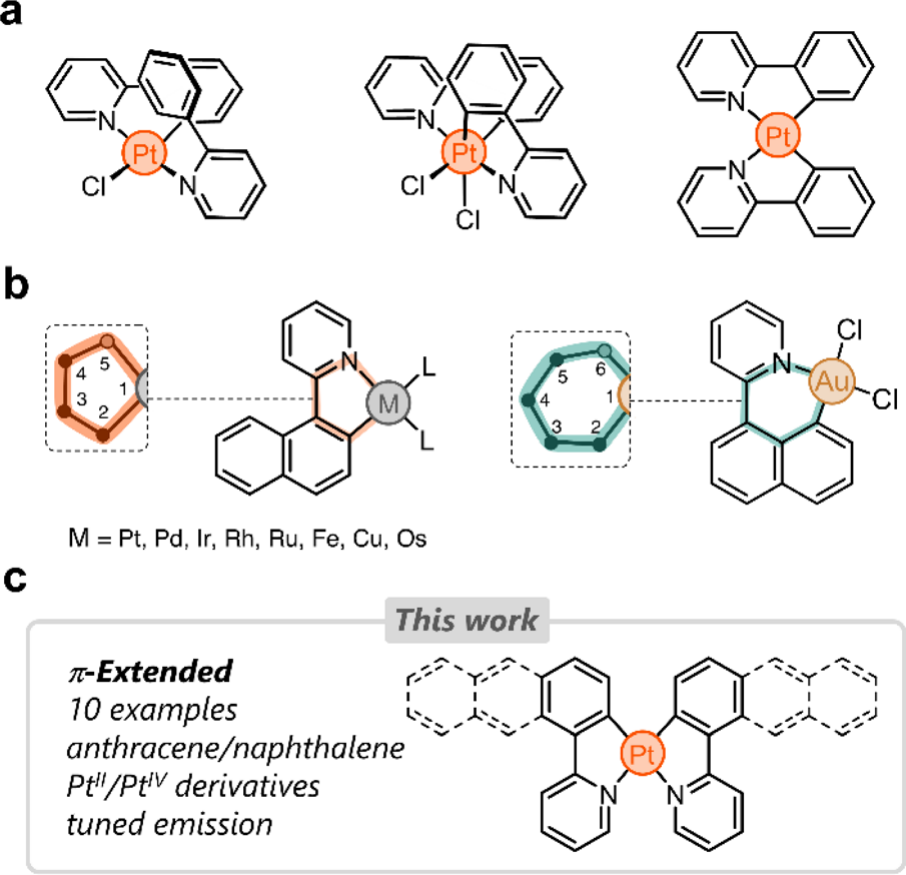
(a) Selected examples of cyclometalated Pt^II^ and Pt^IV^ complexes based on 2-phenylpyridine. (b) Dual binding behavior of 2-(naphthalen-1-yl)pyridine with various metals. (c) This work's overview.

A number of cyclometalated Pt species reported to date rely on phenylpyridine-based ligands.^8,18–30^ The simplest examples of this family consist of 2-phenylpyridine and Pt^II^ or Pt^IV^ centers (Fig. 1a). Several research groups have tailored these systems in different directions to fine-tune the photophysical properties of the resulting complexes.^31–33^ Our group has been particularly inspired by these and other examples to incorporate phenylpyridine-based complexes in supramolecular assemblies,^34^ focusing primarily on (i) bridging phenylpyridines with a single oxygen atom,^35^ (ii) appending ring molecules to phenylpyridines,^36,37^ and (iii) incorporating flexible linkers to shape Pt-bridged macrocycles.^38^

Despite the growing interest in phenylpyridine ligands, studies on derivatives containing π-extended polyaromatics are scarce. Specifically, only 2-(naphthalen-1-yl)pyridine has been investigated in the cyclometalation of Pt,^39^ Pd,^40^ Ir,^41^ Rh,^42^ Ru,^43^ Fe,^44^ Cu,^45^ and Os.^46^ In these examples, the ligand binds to the metal *via* the pyridine's nitrogen donor and through a carbon atom from the naphthalenyl group (2-position, C2). This results in the formation of 5-membered metallacycles (Fig. 1b).^47^ When the same ligand forms a complex with Au, the naphthalenyl unit binds to the metal through the carbon at position 8 (C8), yielding a 6-membered metallacycle (Fig. 1b). This regioselective behavior has been observed only in heteroleptic Au complexes to date.^48^ We hypothesized that the dual binding behavior that the naphthalenyl motif displays could be exploited in other polyaromatics appended to a pyridine donor. This could uncover new directions in chemical reactivity as well as enable tailoring of the emission in the resulting Pt complexes.

Here we investigate the cyclometalation of phenyl-, naphthalenyl-, and anthracenylpyridine ligands (Fig. 1c). By exploiting the dual binding behavior of naphthalenyl and anthracenyl groups, we have isolated ten new cyclometalated complexes, with Pt^II^ and Pt^IV^ centers. These display tunable photoluminescence from blue to infrared light. All complexes have been characterized and analyzed through 1-D and 2-D nuclear magnetic resonance (NMR) spectroscopy, UV-vis spectroscopy, fluorescence spectroscopy, high-resolution mass spectrometry (HRMS), density-functional theory (DFT), and single-crystal X-ray diffraction (SCXRD).

## Results and discussion

**Scheme 1:**
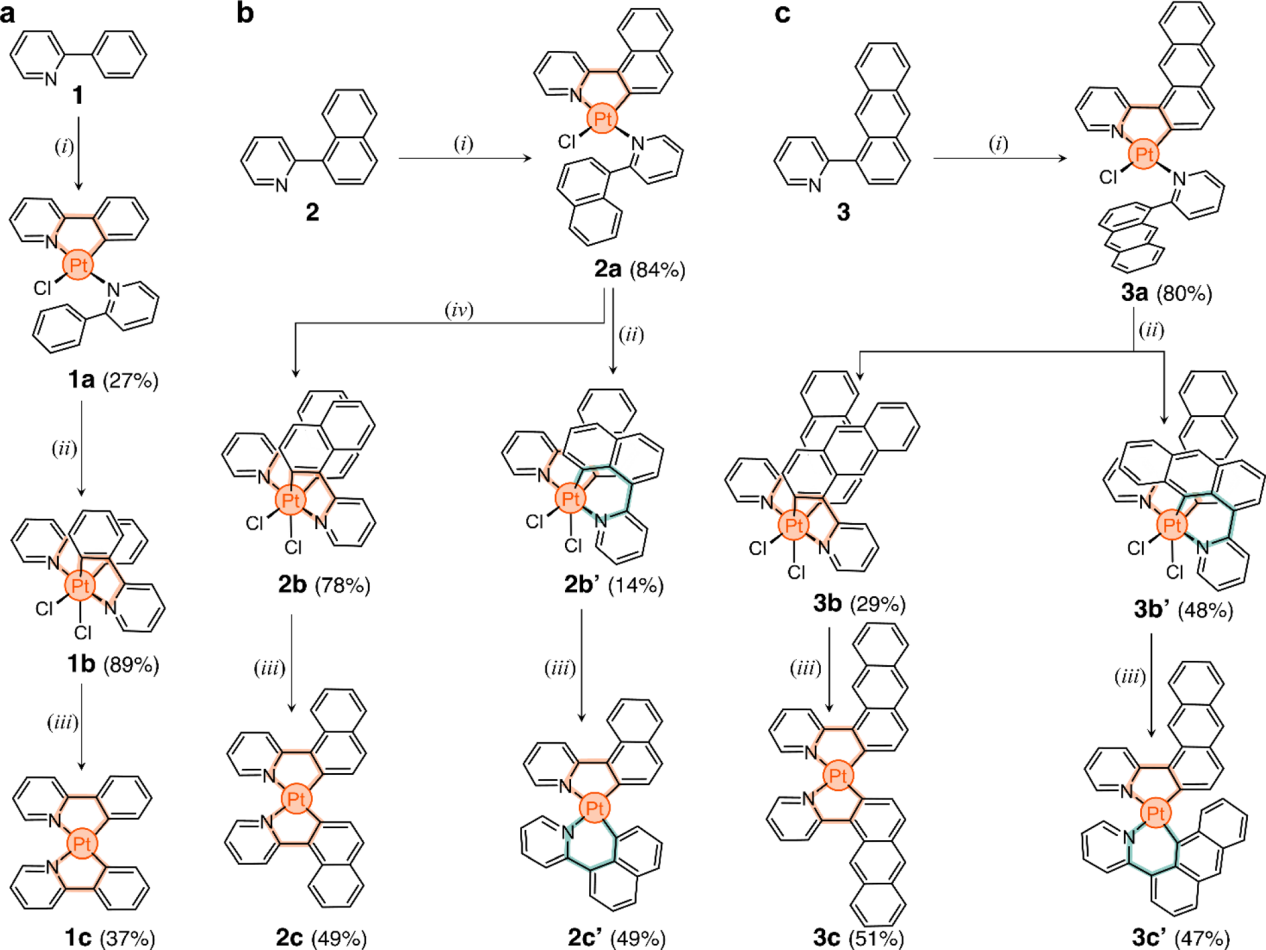
Synthesis of cyclometalated Pt complexes using (a) phenyl-, (b) naphthalenyl-, and (c) anthracenylpyridine ligands 1, 2, and 3. Reaction conditions: (i) K_2_[PtCl_4_], H_2_O, 2-ethoxyethanol, 80 °C, N_2_; (ii) PhICl_2_, DCM, 25 °C; (iii) *t*-BuOK, THF, 60 °C, N_2_; (iv) PhICl_2_, 1,1,2,2-tetrachloroethane, 130 °C.

The three target ligands are shown in Scheme 1a–c. Compound 1 (2-phenylpyridine) was purchased and used without purification. Ligand 2-(naphthalen-1-yl)pyridine (2) was synthesized through the Suzuki cross-coupling of naphthalen-1-ylboronic acid and 2-bromopyridine (91% yield).^49^ 2-(Anthracen-1-yl)pyridine (3) was obtained in three steps. First, 1-aminoanthracene-9,10-dione was converted to 1-bromoanthracene *via* a Sandmeyer reaction, followed by reduction.^50^ The product was borylated *via* a Miyaura borylation,^51^ and then coupled with 2-bromopyridine to obtain ligand 3 in 35% overall yield (full details in ESI†).

In the direct metalation of 2-phenylpyridine, the ligand (1) is typically reacted with K_2_[PtCl_4_] in polar media, leading to the formation of the Pt^II^ square planar complex 1a (Scheme 1a).^20^ This compound has one cyclometalated phenylpyridine (forming a 5-membered metallacycle) and another one only *N*-coordinated, with a free phenyl ring. The coordination sphere of the metal center is completed by a Cl^−^ ion. With this in mind, we tested the direct metalation of ligands 2 and 3, anticipating that their reactivity would be similar to 1.

**Fig. 2:**
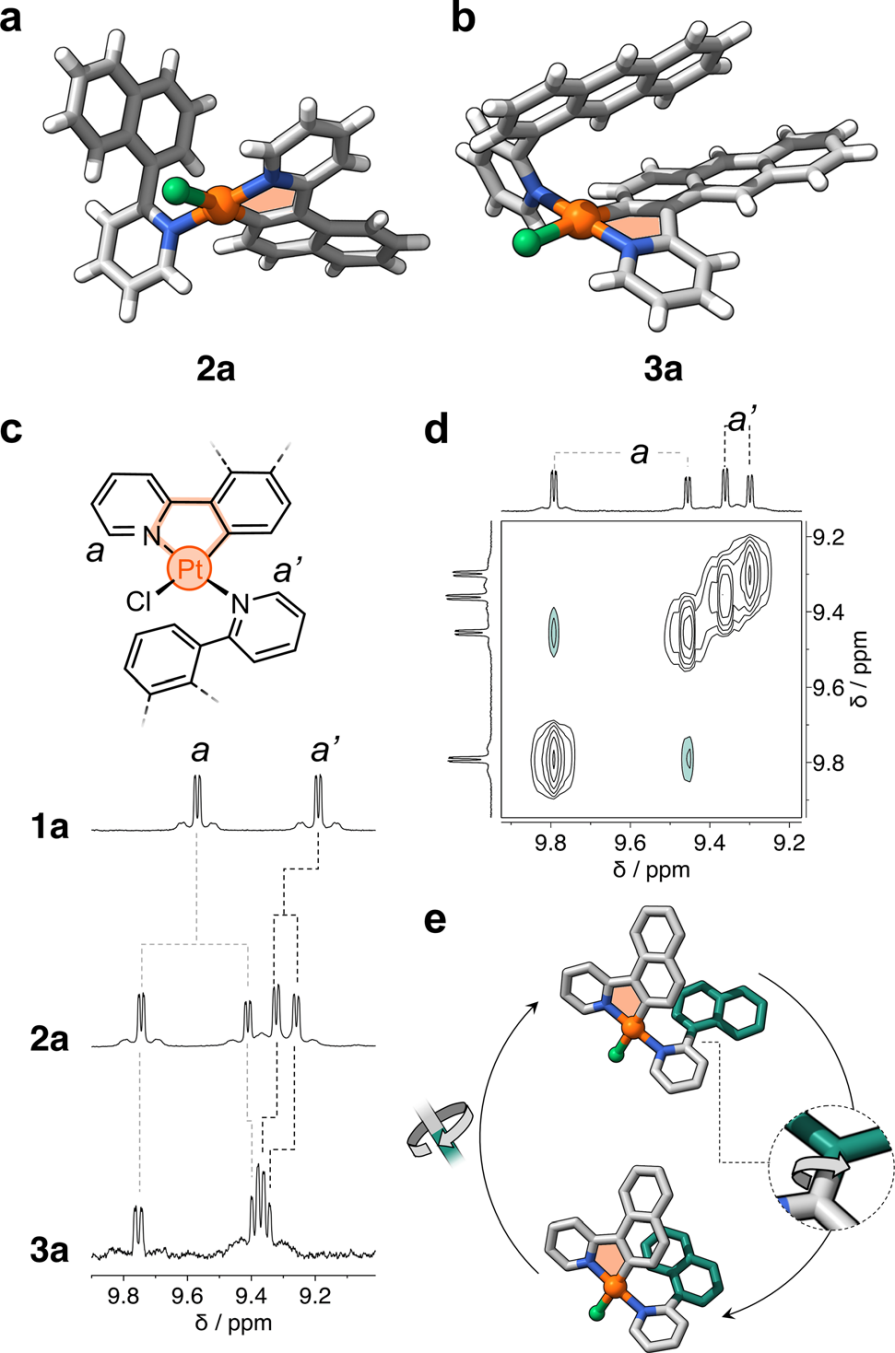
Solid-state molecular structures of complexes (a) 2a and (b) 3a. (c) Representative chemical structure of complexes 1a, 2a, and 3a. Partial ^1^H NMR spectra (400 MHz, DCM-*d*_2_) of such complexes is shown in this panel. (d) Partial EXSY NMR spectrum (600 MHz, DCM-*d*_2_) of complex 2a showing cross peaks between the signals of two atropisomers. (e) Proposed rotational dynamics observed in complex 2a in solution. The structures correspond to the two DFT-computed isomers. Hydrogen atoms omitted for clarity. The cyclometalated and free-rotating naphthalenyl moieties are colored grey and green, respectively.

After metalation, complexes 2a and 3a (Scheme 1b and c) were isolated and their structures were unequivocally assigned by SCXRD. Both molecules have one cyclometalated ligand and another one coordinated only through the pyridine unit (Fig. 2a). Crystals of 2a have two molecules in the asymmetric unit, both with the cyclometalated and free naphthalenyl groups facing opposite directions. Similarly, 3a has two molecules in the asymmetric unit, one that has a similar geometry to 2a and a second one with the free and cyclometalated anthracenyl groups facing in the same direction, with an interplanar distance averaging 5.75 Å and a 48° deviation from coplanarity (see Fig. 2b).

Complexes 2a and 3a were characterized in solution using ^1^H NMR spectroscopy; the recorded spectra were more intriguing than expected (full data in Fig. S1†). For comparison, complex 1a displays distinct signals for each of its phenylpyridine units,^20^ with resonances attributed to protons *a* and *a*′ at 9.6 and 9.2 ppm (Fig. 2c), respectively. However, for 2a and 3a, each of these resonances split into two signals (*e.g.*, *d* = 9.6, 9.3, 9.23 and 9.17 ppm for 2a, Fig. 2c). Exchange spectroscopy (EXSY) NMR of 2a and 3a confirmed that the two sets of protons undergo exchange at room temperature (Fig. 2d and S2†). This suggested that in solution two atropisomers may interconvert through slow rotation of the unbound aryl units (naphthalenyl and anthracenyl) in 2a and 3a.

The initial support for this hypothesis comes from the solid-state structure of 3a, which revealed two distinct atropisomers in the crystal lattice (Fig. S3†). Subsequent investigation using variable-temperature ^1^H NMR spectroscopy showed that the exchanging resonances for both 2a and 3a broaden as the temperature exceeds 90 °C (see Fig. S4 and S5†). DFT analysis showed that the interconversion is likely due to slow rotation about the C–C bond that connects the unbound aryl groups with the pyridine unit (Fig. 2e and ESI†). The computed activation barriers are 19.2 and 20.4 kcal mol^−1^ for 2a and 3a, respectively. These values are in good agreement with those obtained experimentally by ^1^H–^1^H EXSY NMR spectroscopy, which correspond to 18.4 (2a) and 19.1 kcal mol^−1^ (3a) in DCM-*d*_2_ at 25 °C (Fig. S6 and Table S1†). In contrast, the rotation along the same C–C bond in 1a (containing a phenyl ring) occurs with a much lower energy barrier (7.7 kcal mol^−1^, see ESI†) – this explains the observation of only one species by ^1^H NMR spectroscopy. It is noteworthy that the rotation about the N–Pt bond may have a negligible effect on the exchange process due to its higher activation barrier, estimated to range from 26 to 33 kcal mol^−1^ in 1a, 2a and 3a (see ESI, Fig. S7, S8 and Table S2†).

The unexpected slow interconversion of rotamers raised the question of whether this behavior would influence the reactivity of 2a and 3a towards oxidation (Scheme 1b and c). Typically, treating compound 1a with PhICl_2_ leads to oxidation of the metal center (Pt^II^ to Pt^IV^) and cyclometalation of the free phenyl ring, generating compound 1b (Scheme 1a).^22^ A similar outcome was expected from 2a and 3a, and thus their oxidations were tested.

**Fig. 3:**
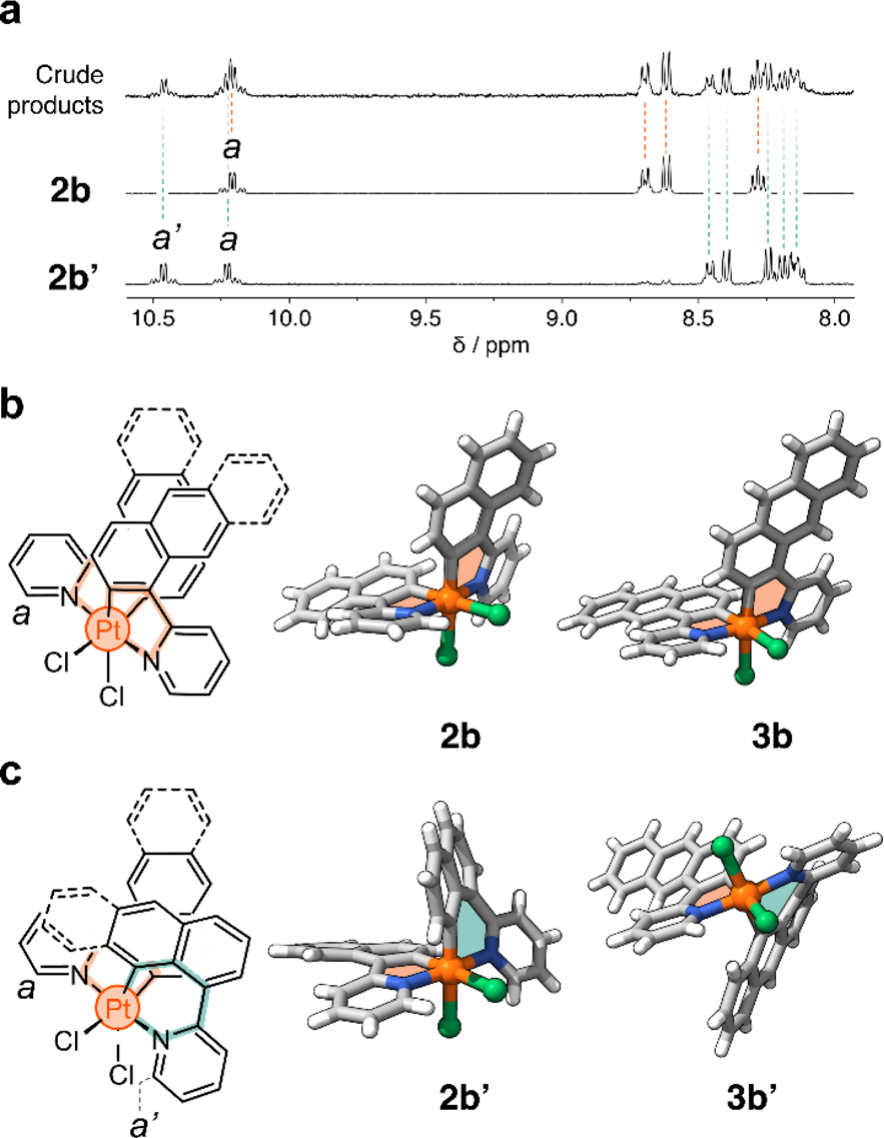
(a) Partial ^1^H NMR spectra (400 MHz, DCM-*d*_2_) of the reaction crude product obtained from the oxidation of 2a. (b) Chemical structure and solid-state structures of complexes (b) 2b and 3b, and (c) 2b′ and 3b′. Panels (b) and (c) contain the proton assignment of resonances *a* and *a*′.

In a representative experiment, a solution of 2a in DCM-*d*_2_ was treated with 1 equiv of PhICl_2_ at room temperature, and the resulting mixture was analyzed by ^1^H NMR spectroscopy (Fig. S9†). Upon addition of the oxidant, all resonances corresponding to 2a disappeared, confirming its conversion into a new species. Based on the precedent of converting 1a to 1b, it was anticipated that the product would also have *C*_2_ symmetry. However, the recorded ^1^H NMR spectrum showed many resonances (Fig. 3a), suggesting the formation of more than one product. Taking advantage of subtle solubility differences between the products, a new compound, 2b′ (Scheme 1b), was first isolated in 14% yield. HRMS confirmed that 2b′ contains a Pt center and two deprotonated cyclometalating ligands ([2b′ − Cl]^+^, *m*/*z* (exp.) = 638.0953 Da, *m*/*z* (calc.) = 638.0963 Da). The ^1^H NMR spectrum of 2b′ showed individual sets of resonances for each naphthalenylpyridine unit, with the α-pyridine protons *a* and *a*′ appearing at 10.2 and 10.0 ppm (Fig. 3a). The solid-state structure (SCXRD) of 2b′ proved that each ligand binds to Pt with a distinct coordination mode. As shown in Fig. 3c, one of the ligands coordinates to the Pt center *via* the C2 naphthalenyl carbon atom, forming a 5-membered metallacycle. The second ligand cyclometalates to Pt generating a 6-membered ring *via* C8-ligation to the naphthalenyl group.

We were unable to isolate the anticipated product 2b (Scheme 1b) in sufficient purity. DFT calculations performed on both 2b and 2b′ revealed that 2b is thermodynamically more stable than 2b′ by 3–4 kcal mol^−1^ (see ESI, Table S3 and S4†). Considering this, 2a was treated with PhICl_2_ in 1,1,2,2-tetrachloroethane at 130 °C. A ^1^H NMR spectrum of the isolated product showed only one set of signals (see, *e.g.*, proton *a* at 10.1 ppm, Fig. 3a), indicating the selective formation of 2b. This compound (78% yield) was analyzed by SCXRD (Fig. 3b), confirming that it contains a Pt^IV^ center coordinated to two Cl^−^ ions and two cyclometalating ligands. Similar to 1b,^22^2b also contains two ligands forming 5-membered metallacycles (C2-ligated).

Oxidation of the anthracene-based compound 3a was also tested, resulting in the detection of two products in a 1 : 2 ratio in the crude reaction product (Fig. S10†). Compounds 3b and 3b′ (Scheme 1c) were obtained in 29% and 48% yield, respectively, after purification. Notably, we were able to purify 3b and 3b′ by silica gel column chromatography, in contrast to the more challenging purification of 2b and 2b′. SCXRD analysis confirmed that the two complexes have analogous structures to 2b and 2b′ (see Fig. 3b and c). Compound 3b′ has 5- and 6-membered metallacycles, with the Pt^IV^ center bound to the C2 and C9 carbon atoms of the anthracenyl units, respectively, while 3b has two 5-membered metallacycles (C2-bound).

We envisioned that the unexpected reactivity of 2a and 3a, enabled by the dual-binding behavior of anthracenyl and naphthalenyl groups, would serve as a unique handle to trap other unpredicted cyclometalated species, especially in the form of Pt^II^ complexes. For this, reduction of the four Pt^IV^ species 2b, 2b′, 3b, and 3b′ was tested (Scheme 1b and c). All compounds were heated at 60 °C with 20 equiv of *t*-BuOK in THF under a N_2_ atmosphere.^52^ The reaction mixtures were purified using column chromatography and the expected products were isolated in yields of 47% to 51% (details in ESI†).

**Fig. 4:**
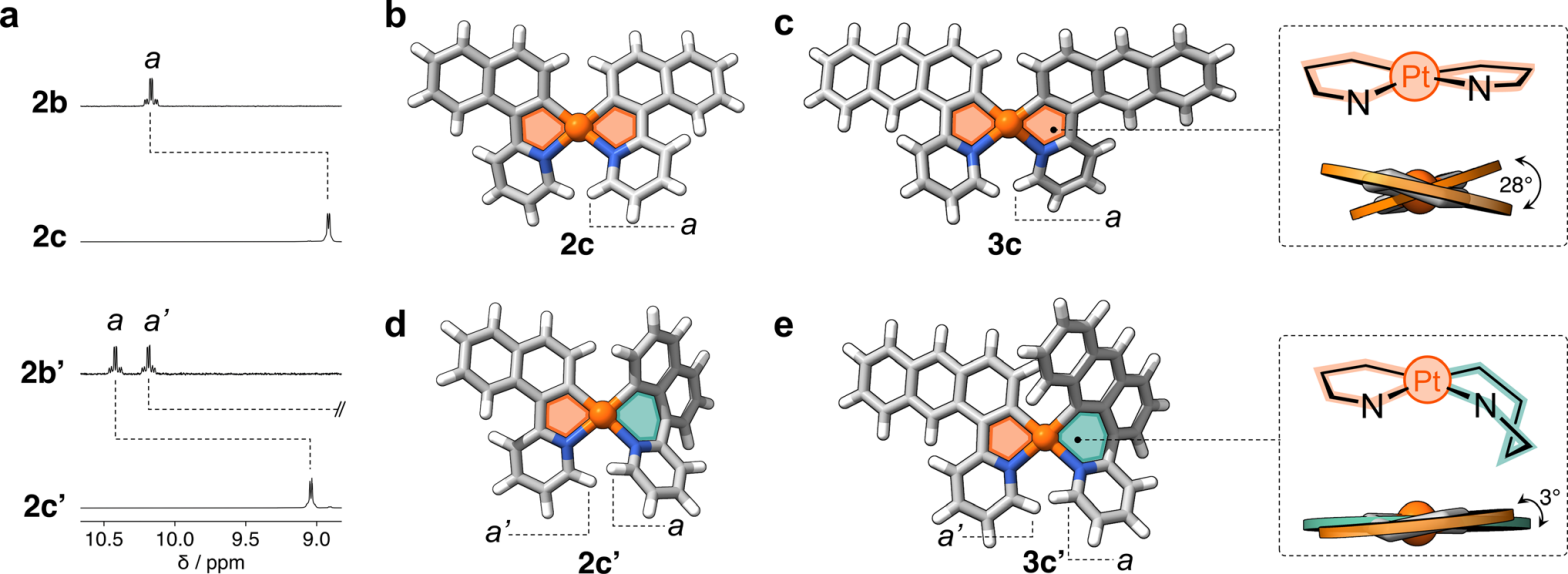
(a) Partial ^1^H NMR spectra (400 MHz, DCM-*d*_2_) of complexes 2c and 2c′ and their precursors 2b and 2b′. (b) Crystal structures as determined by SCXRD of complexes (b) 2c, (c) 3c, and DFT-minimized structures of (d) 2c′ and (e) 3c′. Panels (c) and (e) depict the conformation of the 5- and 6-membered metallacycles as well as the dihedral angles in complexes 3c and 3c′.

Fig. 4a shows the partial ^1^H NMR spectra of compounds 2c and 2c′ (full data in ESI†). In comparison to their precursors, 2b and 2b′, some of the resonances (*e.g.*, *a*) show an upfield shift (Δ*δ*_*a*_ = −1.3 ppm and −1.4 ppm, respectively), indicating the successful reduction of Pt^IV^ to the less electrophilic Pt^II^ center. The ^1^H NMR spectrum of 2c′ contains twice the number of resonances as its analogue 2c. This decreased symmetry is also observed in the ^13^C{^1^H} NMR spectrum, with thirty ^13^C signals detected for 2c′ and only fifteen for 2c (Fig. S11†). Similarly, the ^1^H NMR spectrum of 3c′ showed twice as many resonances as that of its analogue 3c, clearly indicating a significant reduction in symmetry. This observation suggests a more complex and less symmetrical environment in 3c′, which is also confirmed by the 38 resonances observed in the ^13^C{^1^H} NMR spectrum of this complex compared to the 19 signals observed for compound 3c (see Fig. S12†).

The molecular structures of complexes 2c and 3c were determined by SCXRD (Fig. 4b and c). Each molecule has two flat 5-membered metallacycles, although with noticeable deviation from the ideal square planar geometry of the Pt center. Specifically, the dihedral angles (*θ*) are 26° and 28° for 2c and 3c, respectively. This deviation, which has been observed in previous studies,^19^ originates from steric repulsion between hydrogen atoms on the ligand (*e.g. a* in Fig. 4b and c). Unfortunately, attempts to obtain single crystals of 2c′ and 3c′ failed, so insights into their structures were gained through DFT computations (see ESI, Table S5†). Complexes 2c′ and 3c′ show minimal deviation from first coordination square planar geometry, with *θ* of 5° and 3°, respectively (Fig. 4d and e). This distortion is attributed to the boat-like conformation adopted by the six-membered metallacycles, where the aryl and pyridine rings deviate from the plane of the coordination sphere. This deviation effectively mitigates the steric repulsion between adjacent hydrogen atoms in complexes 2c and 3c.

After isolating ten different complexes from ligands 2 and 3, we investigated their emission properties and compared them with those of 1a, 1b, and 1c,^8,19,20^ (Fig. S13 to S16†). All complexes except for 1c exhibited long emission lifetimes (up to 15 μs) in solution and relatively low photoluminescent quantum yields (*Φ*). Compound 2a had the highest *Φ* (2.3%, Table S6†).

**Fig. 5:**
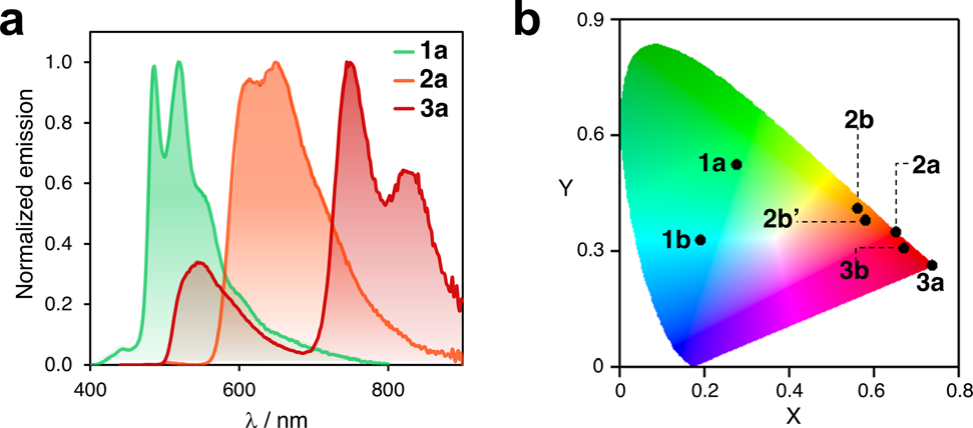
(a) Emission spectra of complexes 1a, 2a, and 3a in DCM at 25 °C. (b) CIE plot of selected Pt^II^ and Pt^IV^ cyclometalated complexes containing ligands 1, 2 and 3. Data corresponding to DCM solutions recorded at 25 °C.

In addition, the collected data shed light on several important aspects. First, the compounds exhibit a concomitant redshift of their absorption and emission bands as the ligand's π-conjugation extends from phenyl (1) to naphthalenyl (2) to anthracenyl (3). For instance, in solution, compounds 1a, 2a, and 3a emit green, orange, and red-to-infrared light, respectively (see Fig. 5a). Related complexes have been previously reported in the literature to undergo photoinduced isomerization.^30^ However, the consistency of the UV-vis absorption and excitation spectra indicated that this is not the case for the compounds investigated here. It should be noted that the excitation spectra for some complexes were not recorded owing to their very weak emission (see Fig. S15†). The CIE plot in Fig. 5b illustrates the significant differences in emission that can be achieved within this family of compact emitters. Additionally, the sets of isomers, such as 2b/2b′ and 3b/3b′, show similar photophysical properties. The emission bands of 2c and 2c′ differ in a range of 10 to 42 nm in solution, in a glassy matrix, and in the solid state (Fig. S17†), indicating that subtle structural differences can fine-tune emission properties. Finally, for some complexes, luminescence is activated by switching from phenyl to naphthalenyl or anthracenyl. Although compound 1c is not luminescent in solution, the analogous complexes formed with ligands 2 and 3 are emissive. This may be due to the significant deviation from planarity that 1c (*θ* > 80°) shows in the excited state, which promotes non-radiative decay.^53,54^ However, this phenomenon may be reduced in complexes 2c, 2c′, 3c, and 3c′ with bulkier ligands. Altogether, these observations indicate potential areas for further exploration, such as customizing the emission properties of cyclometalated Pt complexes by expanding to other polyaromatics. In addition, it may be beneficial to explore the circularly polarized emission of the complexes 2c′ and 3c′, and other parent compounds, following chiral resolution.

## Conclusions

In summary, our exploration of cyclometalated platinum complexes bearing compact, π-extended ligands has led to significant findings. The hindered rotation around the aryl–pyridine bond led to the discovery of unique rotamers, which had a pivotal impact on both structural and reactivity aspects. In particular, selective oxidation yielded Pt^IV^ complexes with symmetric and asymmetric structures, providing valuable insights into their reactivity and isolation. Our synthesized complexes show promising photophysical properties, particularly in the near-infrared region, which makes them suitable for use in bioimaging and optical sensors. This study advances the understanding of Pt complexes with extended π-conjugation, providing opportunities for future research and applications.

## Author contributions

M. J. MacLachlan, M. A. Soto and S. Ota designed the research. S. Ota conducted the experiments. S. Ota, M. A. Soto, B. O. Patrick and S. Kamal analyzed the data. F. Lelj performed DFT calculations. M. A. Soto and S. Ota co-write the manuscript. M. J. MacLachlan and M. A. Soto conducted general guidance, project directing, and manuscript revision.

## Conflicts of interest

There are no conflicts to declare.

## Supplementary Material

SC-015-D4SC04799K-s001

SC-015-D4SC04799K-s002

## Data Availability

All experimental procedures, mechanistic investigations, characterisation data, NMR spectra and computational data can be found in the article or in the ESI.† Crystallographic data for compounds analyzed by SCXRD have been deposited at the CCDC under deposition numbers CCDC Deposition Numbers: 2357023 (2b′), 2357024 (2b), 2357025 (3b), 2357026 (2c), 2357027 (3c), 2357028 (3a), 2357029 (2a), and 2357030 (3b′), and can be obtained from https://www.ccdc.cam.ac.uk/

## References

[cit1] van Koten G. (1989). Pure Appl. Chem..

[cit2] Paw W., Eisenberg R. (1997). Inorg. Chem..

[cit3] Chow P.-K., Cheng G., Tong G. S. M., To W.-P., Kwong W.-L., Low K.-H., Kwok C.-C., Ma C., Che C.-M. (2015). Angew. Chem., Int. Ed..

[cit4] Soto M. A., Kandel R., MacLachlan M. J. (2021). Eur. J. Inorg. Chem..

[cit5] Zucca A., Petretto G. L., Stoccoro S., Cinellu M. A., Manassero M., Manassero C., Minghetti G. (2009). Organometallics.

[cit6] Nikolaeva M. V., Puzyk M. V. (2013). Opt. Spectrosc..

[cit7] Craig C. A., Garces F. O., Watts R. J., Palmans R., Frank A. J. (1990). Coord. Chem. Rev..

[cit8] Cho J.-Y., Suponitsky K. Y., Li J., Timofeeva T. V., Barlow S., Marder S. R. (2005). J. Organomet. Chem..

[cit9] Septiadi D., Aliprandi A., Mauro M., De Cola L. (2014). RSC Adv..

[cit10] Colombo A., Fiorini F., Septiadi D., Dragonetti C., Nisic F., Valore A., Roberto D., Mauro M., De Cola L. (2015). Dalton Trans..

[cit11] Wang X., Wang X., Guo Z. (2015). Acc. Chem. Res..

[cit12] Xu H., Chen R., Sun Q., Lai W., Su Q., Huang W., Liu X. (2014). Chem. Soc. Rev..

[cit13] Fleetham T., Li G., Li J. (2017). Adv. Mater..

[cit14] Kato M. (2007). Bull. Chem. Soc. Jpn..

[cit15] Aliprandi A., Genovese D., Mauro M., De Cola L. (2015). Chem. Lett..

[cit16] Parrinello G., Stille J. K. (1987). J. Am. Chem. Soc..

[cit17] Markó I. E., Stérin S., Buisine O., Mignani G., Branlard P., Tinant B., Declercq J.-P. (2002). Science.

[cit18] Black D. S., Deacon G. B., Edwards G. L. (1994). Aust. J. Chem..

[cit19] Chassot L., Mueller E., Von Zelewsky A. (1984). Inorg. Chem..

[cit20] Mdleleni M. M., Bridgewater J. S., Watts R. J., Ford P. C. (1995). Inorg. Chem..

[cit21] Yamaguchi T., Yamazaki F., Ito T. (2001). J. Am. Chem. Soc..

[cit22] Newman C. P., Casey-Green K., Clarkson G. J., Cave G. W. V., Errington W., Rourke J. P. (2007). Dalton Trans..

[cit23] Whitfield S. R., Sanford M. S. (2008). Organometallics.

[cit24] Mamtora J., Crosby S. H., Newman C. P., Clarkson G. J., Rourke J. P. (2008). Organometallics.

[cit25] Jenkins D. M., Bernhard S. (2010). Inorg. Chem..

[cit26] Juliá F., Aullón G., Bautista D., González-Herrero P. (2014). Chem.–Eur. J..

[cit27] Juliá F., Bautista D., Fernández-Hernández J. M., González-Herrero P. (2014). Chem. Sci..

[cit28] Juliá F., Bautista D., González-Herrero P. (2016). Chem. Commun..

[cit29] Juliá F., González-Herrero P. (2016). Dalton Trans..

[cit30] Juliá F., García-Legaz M.-D., Bautista D., González-Herrero P. (2016). Inorg. Chem..

[cit31] Vezzu D. A. K., Deaton J. C., Jones J. S., Bartolotti L., Harris C. F., Marchetti A. P., Kondakova M., Pike R. D., Huo S. (2010). Inorg. Chem..

[cit32] Fukagawa H., Shimizu T., Hanashima H., Osada Y., Suzuki M., Fujikake H. (2012). Adv. Mater..

[cit33] López-López J. C., Bautista D., González-Herrero P. (2023). Inorg. Chem..

[cit34] Soto M. A., MacLachlan M. J. (2024). Chem. Sci..

[cit35] Soto M. A., Carta V., Andrews R. J., Chaudhry M. T., MacLachlan M. J. (2020). Angew. Chem., Int. Ed..

[cit36] Soto M. A., Carta V., Cano M. T., Andrews R. J., Patrick B. O., MacLachlan M. J. (2022). Inorg. Chem..

[cit37] Soto M. A., Chaudhry M. T., Matharu G. K., Lelj F., MacLachlan M. J. (2023). Angew. Chem., Int. Ed..

[cit38] Soto M. A., Carta V., Suzana I., Patrick B. O., Lelj F., MacLachlan M. J. (2023). Angew. Chem., Int. Ed..

[cit39] Yang H., Li H., Yue L., Chen X., Song D., Yang X., Sun Y., Zhou G., Wu Z. (2021). J. Mater. Chem. C.

[cit40] Kondrashov M., Provost D., Wendt O. F. (2016). Dalton Trans..

[cit41] Denisov S. A., Cudré Y., Verwilst P., Jonusauskas G., Marín-Suárez M., Fernández-Sánchez J. F., Baranoff E., McClenaghan N. D. (2014). Inorg. Chem..

[cit42] Luo C.-Z., Gandeepan P., Jayakumar J., Parthasarathy K., Chang Y.-W., Cheng C.-H. (2013). Chem.–Eur. J..

[cit43] Fernández-Salas J. A., Manzini S., Piola L., Slawin A. M. Z., Nolan S. P. (2014). Chem. Commun..

[cit44] Zhang G., Lv G., Pan C., Cheng J., Chen F. (2011). Synlett.

[cit45] Jin J., Wen Q., Lu P., Wang Y. (2012). Chem. Commun..

[cit46] Cerón-Camacho R., Hernández S., Le Lagadec R., Ryabov A. D. (2011). Chem. Commun..

[cit47] Omae I. (2017). J. Organomet. Chem..

[cit48] Kondrashov M., Raman S., Wendt O. F. (2014). Chem. Commun..

[cit49] Njogu R. E. N., Fodran P., Tian Y., Njenga L. W., Kariuki D. K., Yusuf A. O., Scheblykin I., Wendt O. F., Wallentin C.-J. (2019). Synlett.

[cit50] Tsvetkov N. P., Gonzalez-Rodriguez E., Hughes A., dos Passos Gomes G., White F. D., Kuriakose F., Alabugin I. V. (2018). Angew. Chem., Int. Ed..

[cit51] Wang X., Liu W.-G., Liu L.-T., Yang X.-D., Niu S., Tung C.-H., Wu L.-Z., Cong H. (2021). Org. Lett..

[cit52] Allison I., Lim H., Shukla A., Ahmad V., Hasan M., Deshmukh K., Wawrzinek R., McGregor S. K. M., Clegg J. K., Divya V. V., Govind C., Suresh C. H., Karunakaran V., Unni K. N. N., Ajayaghosh A., Namdas E. B., Lo S.-C. (2019). ACS Appl. Electron. Mater..

[cit53] Rausch A. F., Murphy L., Williams J. A. G., Yersin H. (2012). Inorg. Chem..

[cit54] Wu J., Xu B., Xu Y., Yue L., Chen J., Xie G., Zhao J. (2023). Inorg. Chem..

