# Supplementary material for: π-Extended ligands with dual-binding behavior: hindered rotation unlocks unexpected reactivity in cyclometalated Pt complexes
Source: Chem Sci. 2024 Sep 11;15(36):14644–50. doi: 10.1039/d4sc04799k (PMC11388036; doi:10.1039/d4sc04799k)
Supplement: SC-015-D4SC04799K-s001 [file SC-015-D4SC04799K-s001.pdf]

Supplementary Information (SI) for Chemical Science.
This journal is © The Royal Society of Chemistry 2024

Supporting information for

π-Extended Ligands with Dual-Binding Behavior: Hindered Rotation Unlocks Unexpected Reactivity in Cyclometalated Pt Complexes

Seiya Ota,[a] Miguel A. Soto,[a]* Brian O. Patrick,[a] Saeid Kamal,[a] Francesco Lelj,[b] and Mark J. MacLachlan[a][c][d]*

[a] Department of Chemistry, University of British Columbia, 2036 Main Mall, Vancouver, V6T 1Z1 Canada
[b] La.M.I.and LaSCAMM INSTM Sezione Basilicata, Dipartiento di Scienze, Università della Basilicata via dell'Ateneo Lucano 10, Potenza, 85100 Italy
[c] Stewart Blusson Quantum Matter Institute, University of British Columbia 2355 East Mall, Vancouver, BC, V6T 1Z4 Canada
[d] WPI Nano Life Science Institute, Kanazawa University, Kanazawa, 920-1192 Japan

Contents

Materials 4

Methods and characterization 4

Computational methods 6

Atropisomers identification 7

Energy barriers for rotamer exchange 11

Experimental determination (compounds **2a** and **3a**) 11

Theoretical estimate (compounds **1a**, **2a**, and **3a**) 12

First oxidation test on **2a** 20

First oxidation test on **3a** 21

Computed relative stability of isomers **2b**/**2b’** and **3b**/**3b’** 22

DFT-minimized structures of Pt^{II} complexes **2c’** and **3c’** 26

Photophysical properties 28

UV-vis spectra 28

Photoluminescence spectra 30

Lifetime measurements 33

Synthesis and characterization 38

Ligand **2** 40

Precursor 1-bromoanthracene 40

Precursor 2-(anthracen-1-yl)-4,4,5,5-tetramethyl-1,3,2-dioxaborolane 41

Ligand **3** 41

Complex **2a** 45

Compound **3a** 53

Complex **2b** 62

Complex **2b’** 65

Compound **3b** 68

Complex **3b’** 69

Complex **2c** 74

Complex **2c’** 78

Pt^{II} complex **3c** 83

Pt^{II} complex **3c’** 87

SCXRD analyses 93

Complex **2a** (mm579) 93

Complex **2b** (mm607) 95

Complex **2b’** (mm567) 96

Complex **2c** (mm585) 98
Complex **3a** (mm587) 100
Complex **3b** (mm589) 102
Complex **3b'** (mm574) 104
Complex **3c** (mm582) 106
References 109

Materials

All commercially available chemicals were purchased from Sigma-Aldrich, TCI, Cambridge Isotope Laboratories and Ambeed, and used without purification. Naphthalen-1-ylboronic acid and *t*-butylnitrite were purchased from TCI. 2-Bromopyridine, Pd(dppf)Cl_2 (DCM adduct) and bis(pinacolato)diboron were purchased from Cambridge Isotope Laboratories. $Pd(PPh_3)_4$, 1-aminoanthraquinone, sodium borohydride, and *t*-BuOK were purchased from Sigma-Aldrich. $K_2[PtCl_4]$ was purchased from Ambeed.

Dry tetrahydrofuran (THF) was collected from an Inert PureSolv MD5 purification system. Deuterated solvents (DCM-d_2, chloroform-*d*, and 1,1,2,2-tetrachloroethane-d_2) were purchased from Cambridge Isotope Laboratories and Sigma-Aldrich. Flash column chromatography was carried out using SiliCycle (230–400 mesh) silica gel as the stationary phase.

Methods and characterization

Nuclear magnetic resonance (NMR) experiments were recorded on Bruker AVIII HD 400 MHz, Bruker Avance 400 MHz, and Bruker Neo 600 MHz spectrometers; ^{1}H and $^{13}C\{1H\}$ NMR chemical shifts (δ) are given in parts per million (ppm) relative to TMS, using the residual solvent signal for calibration. *J* values are reported in Hz, and signal multiplicity is denoted as *s* (singlet), *d* (doublet), *t* (triplet), *dd* (double of doublets), *td* (triplet of doublets), *ddd* (doublet of doublets of doublets), and *m* (multiplet). Low-resolution mass spectra (LRMS) were collected on a Waters ZQ spectrometer equipped with an ESCI ion source. High-resolution mass spectra (HRMS) were recorded on an ESI-TOF Waters Micromass LCT spectrometer.

Ligand **1** was prepared from 2-bromopyridine and phenylboronic acid *via* a Suzuki coupling reaction based on literature.[1] Compound **1a** was subsequently obtained by cyclometallation with

K_2PtCl_4.[2] Further oxidation with $PhICl_2$ gave **1b**, and the reduction of this compound with *t*-BuOK gave **1c**.[3,4]

Photophysical measurements. All UV-vis data were collected on a Cary 5000 UV-vis-NIR spectrophotometer, using 10 mm pathlength quartz cuvettes. Photoluminescence spectra were obtained using an Edinburgh FLS1000 fluorimeter, equipped with a double excitation monochromator and powered by a Xe arc lamp as the excitation source. Solution sample measurements were performed using 10 mm quartz cuvette. A Thorlabs FELH0400 longpass filter was placed in the detection path to block scattered excitation.

Quantum yield measurements. Photoluminescent quantum yields (Φ) were estimated relative to quinine sulfate, Rhodamine B, and $[Ru(bpy_3)]Cl_2$. The Φ for these standards were considered as 0.546 (0.5 M H_2SO_4, λ_{exc} = 366 nm), 0.5 (EtOH, λ_{exc} = 450 nm), and 0.04 (H_2O, λ_{exc} = 450 nm), respectively, based on literature reports.[5] All Pt complexes samples were prepared inside a glove box using degassed DCM.

Lifetime measurements. Luminescence lifetime measurements were conducted utilizing a streak camera (Hamamatsu, C7700) in conjunction with a spectrograph (Princeton Instrument SP300). The excitation was provided by a picosecond laser (EKSPLA PL2241), with a 35 ps pulse duration and 355 nm excitation wavelength, operating at 10 Hz repetition rate. All samples were prepared in degassed DCM inside a glove box.

Preparation of doped PMMA films. A mixture of commercial PMMA (665 mg) and the corresponding Pt complexes (**2c** and **2c’**, 0.5 μmol) were dissolved in 5.0 mL of DCM, yielding a

clear solutions. These were then casted onto clean glass slides, and the solvent was left to evaporate at room temperature overnight, resulting in the formation of transparent PMMA films.

Computational methods

The Gaussian suite of programs G016.revC01[6] was used for all geometry and Hessian matrix calculations. In addition, geometries were computed with the range separated dispersion corrected ωB97xD[7] and M06[8] meta-hybrid xc functional. In selected cases, the double hybrid functional B2PLYPD3[9] was used. The basis set for all atoms was 6-31g(d),[10] D95(d),[11] and in selected cases cc-pVDZ, cc-pTVZ and cc-pQVZ basis sets were used. For Pt, the fully relativistic energy consistent pseudopotential was used with cc-pXVZPP (x=D, T, Q) in correspondence with the similar multiple zeta basis on non-Pt atoms.[12] For each example, the level of theory used is indicated as: (non-Pt atom basis set; Pt basis set, Pt pseudopotential/xc-functional/solvent). All structures were confirmed to be minima by computing the corresponding Hessian matrix at the same level of theory. Structures were refined and retested when the analytical computed Hessian generated a maximum displacement larger than the optimization threshold. All integrals have been computed using the "Ultrafine" integration grid. Solvent environment was considered by the self-consistent reaction field (SCRF) approach for continuum solvent model simulation (included in the Gaussian suite).[13]

Atropisomer identification

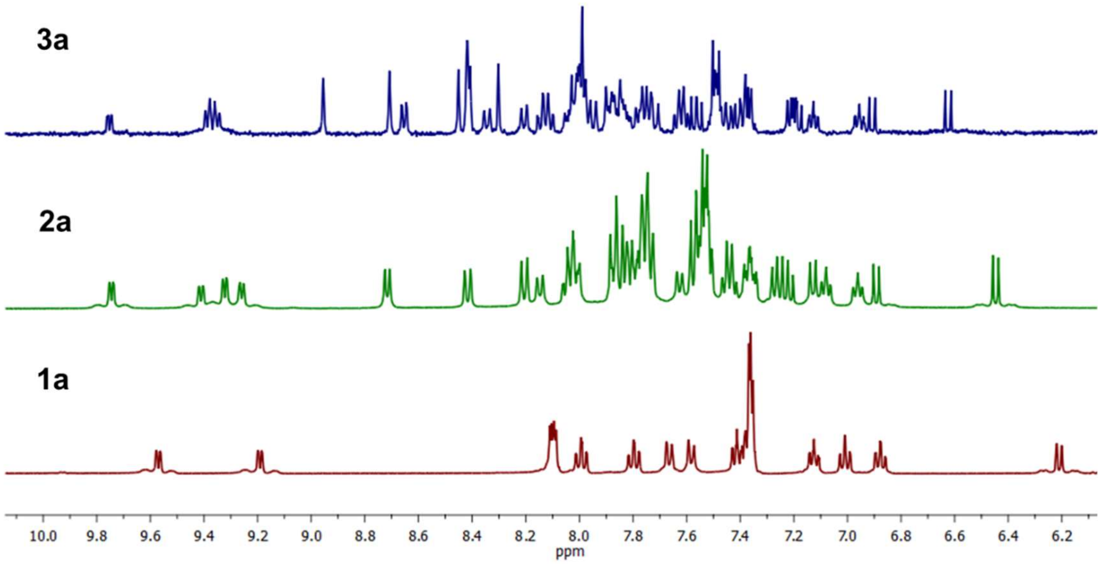

Figure S1. Partial ^{1}H NMR spectra (400 MHz, DCM-d_2) of **1a**, **2a** and **3a.**

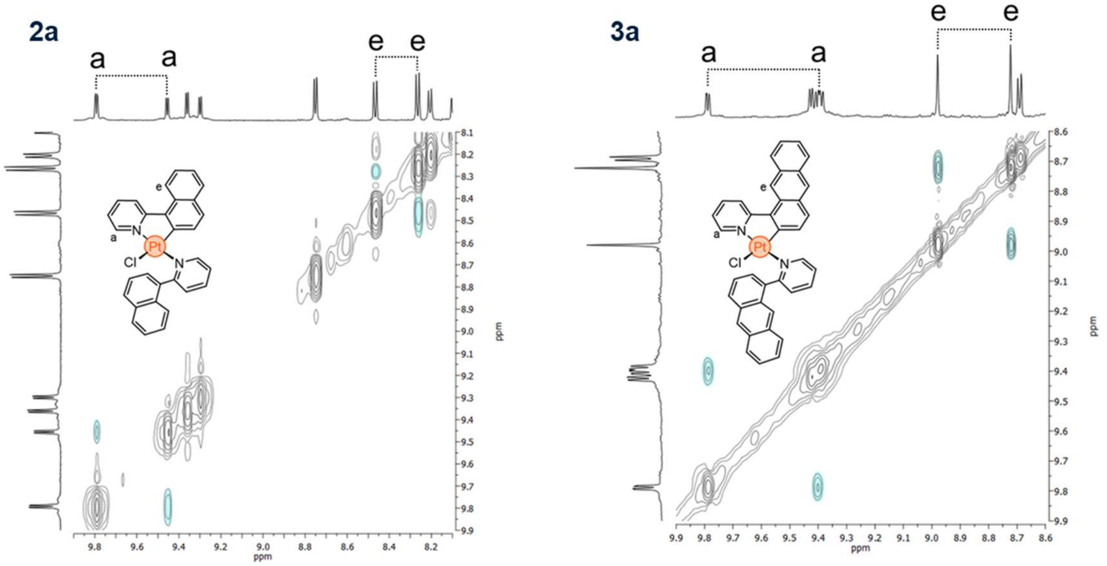

Figure S2. Partial ^{1}H–^{1}H EXSY NMR spectra (600 MHz, DCM-d_2) of **2a** and **3a.**

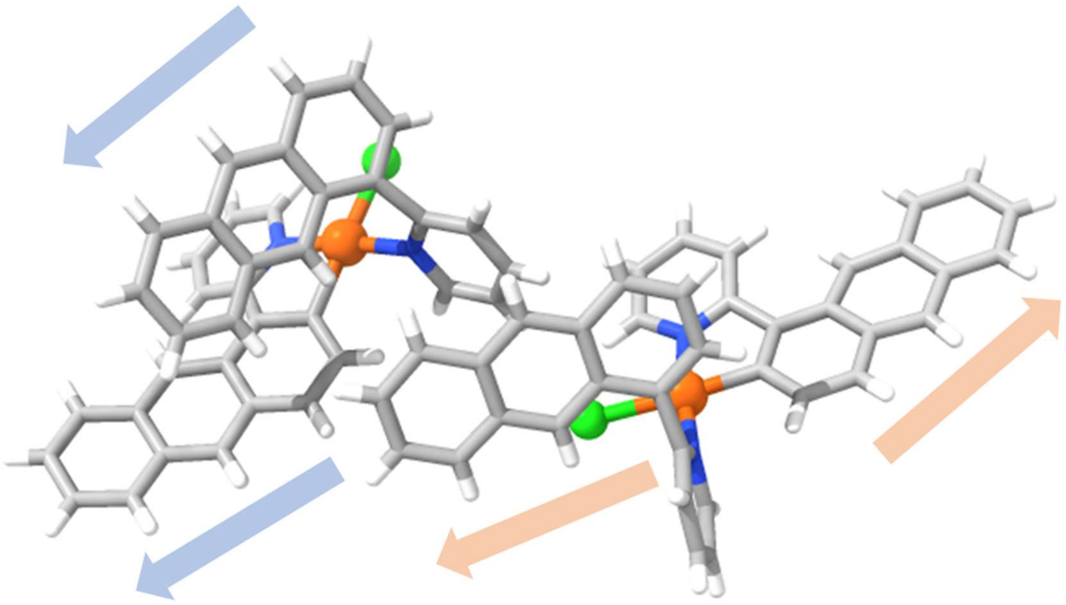

Figure S3. The two isomers of **3a** observed in the asymmetric unit. Arrows indicate the direction of each anthracenyl group.

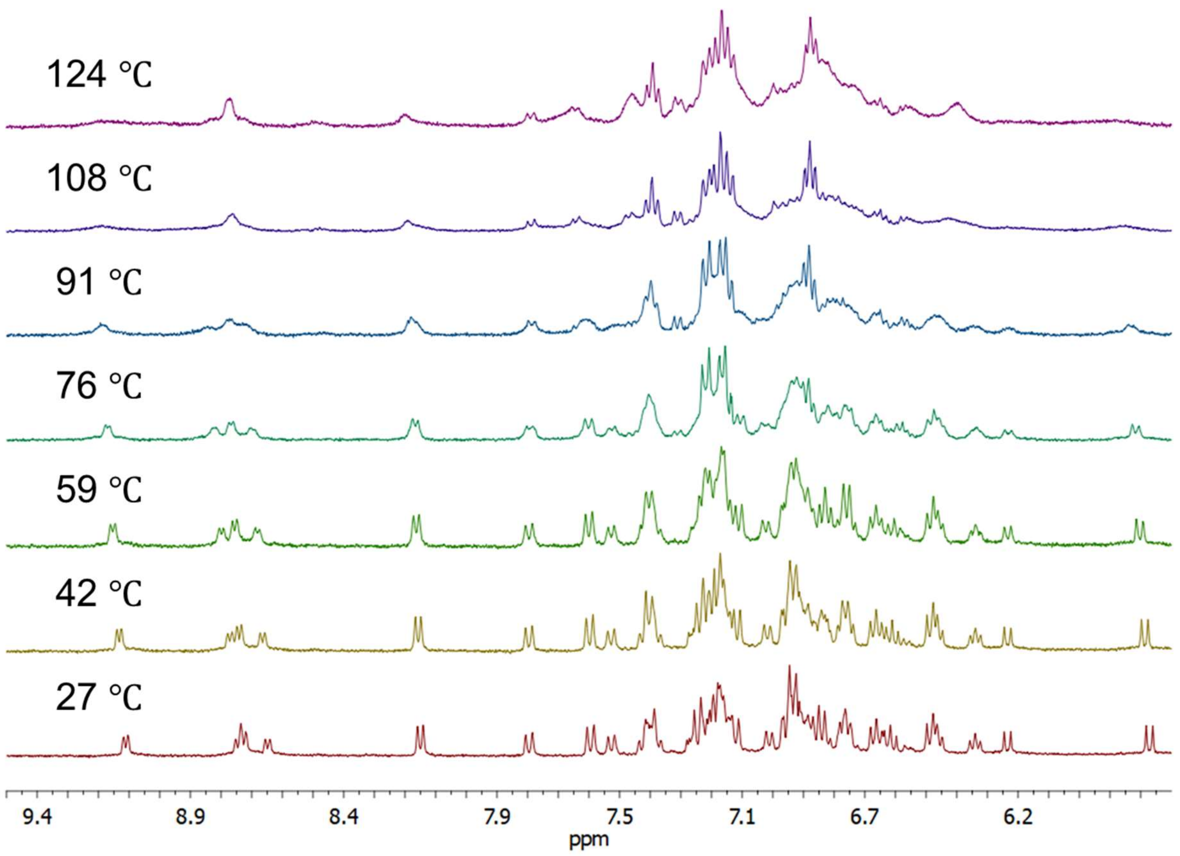

Figure S4. VT ^{1}H NMR spectra (400 MHz, tetrachloroethane-d_2) of **2a**, [**2a**] = 1.0 × 10^{-3} M. The temperature was increased by 15 °C every 20 min up to 124 °C.

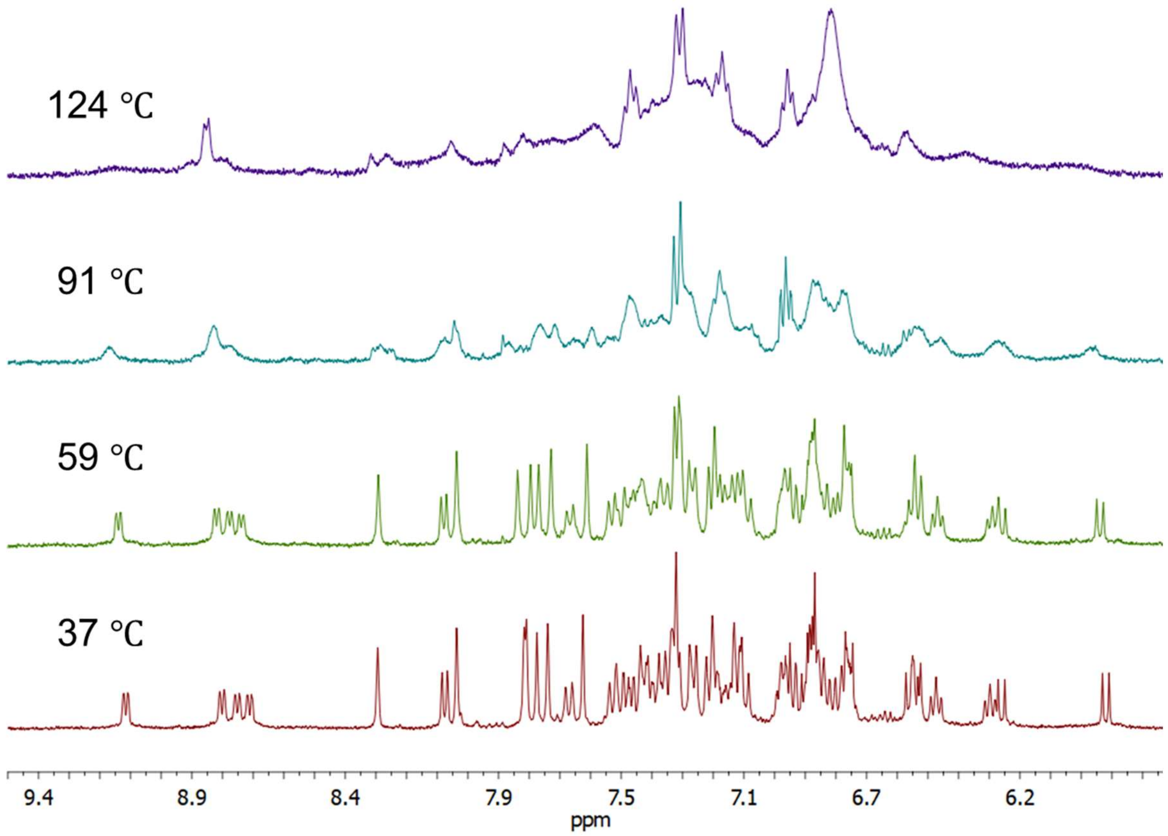

Figure S5. VT ^{1}H NMR spectra (400 MHz, tetrachloroethane-d_2) of **3a**, [**3a**] = 1.0 × 10^{-3} M. The temperature was increased by 30 °C every 20 min up to 124 °C.

Energy barriers for rotamer exchange

Experimental determination

To obtain a rotational barrier, a 2-D EXSY NMR experiment was carried out at 298 K. The recorded spectrum is shown in Figure S6. The normalized intensities of the cross peaks and the diagonal, as well as the calculated exchange rates (*k*) and free activation energies ($\Delta G^{\ddagger}$) are shown in Table S1.

The exchange rates were measured based on the cross-peak-to-diagonal-peak intensity ratio at a single 2D EXSY NMR spectrum. The rate of exchange between two non-coupled spins of the diastereotopic α protons of the pyridine units (denoted spin A and B) were estimated using the following equation:[14]

$$k = \frac{1}{t_m} ln \frac{r+1}{r-1}, \quad (1)$$

where

$$r = \frac{I_{AA}+I_{BB}}{I_{AB}+I_{BA}}, \quad (2)$$

and t_m is the mixing time.

The free energy of activation ($\Delta G^{\ddagger}$) was obtained by the Eyring equation (3).

$$k = \frac{K_B T}{h} e - \frac{\Delta G^{\ddagger}}{RT} \quad (3)$$

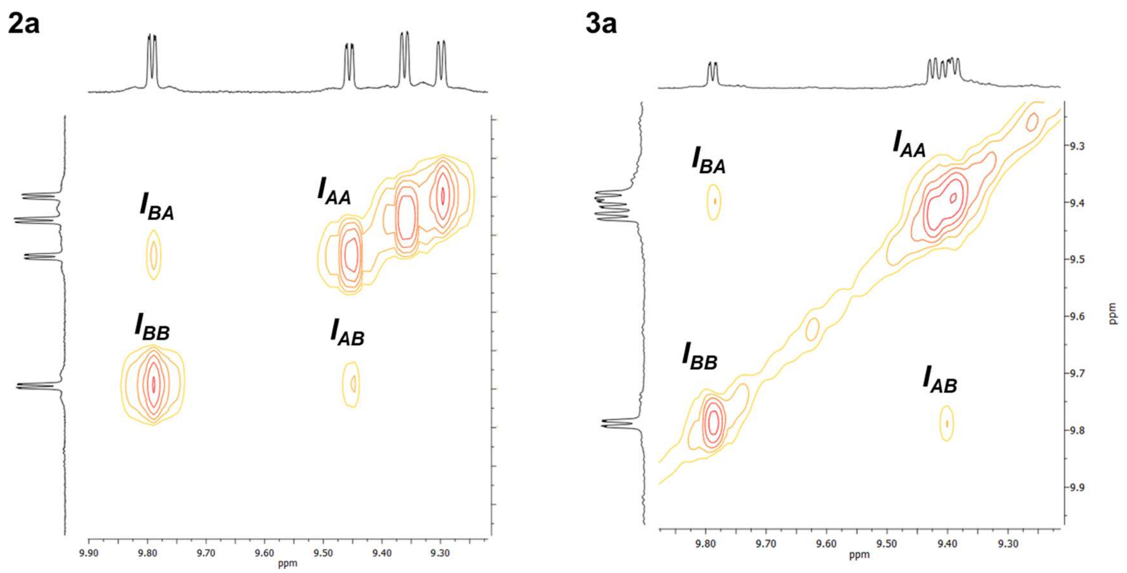

Figure S6. Partial EXSY ^1H-^1H NMR spectrum (600 MHz, DCM-d_2, t_m= 0.8 s) of **2a** and **3a** collected at 298 K.

Table S1. Parameters from 2D EXSY NMR spectra and the calculated *k* and ΔG‡ for **2a** and **3a** (DCM-d_2, 298 K).

	T(K)	I_{AA}	I_{BB}	I_{AB}	I_{BA}	*k* (s^{-1})	Δ*G*‡ (kcal mol^{-1})
2a	298	1.00	0.77	0.07	0.07	0.21	18.4
3a	298	1.00	0.48	0.02	0.02	0.07	19.1

Theoretical estimate

Compounds **1a**, **2a** and **3a** have two single bonds that could allow free rotation. One is the Pt–N bond and the second one is a C–C bond formed between the pyridine (*C2*) and the aryl groups (*C1'*).

In the analyses, we indicate with *A* the ring (naphthalenyl or anthracenyl) directly bound to the pyridine unit, and *B* the further aromatic part of the aryl moiety.

Pt–N torsion

The most extensive set of calculations with different basis set and xc-functionals has been undertaken for the naphthalenyl derivative **2a**. Based on our computations, at the D95(d);cc-pVDZPP,ECP60MDF/wb97XD/DCM level of theory, there are different minima very close in energy resulting from rotation along the Pt–N bond (see examples in Figure S7). All the stable conformations show angles below 90° (*C2* pyridine ring with respect to the average platinum coordination plane). The lowest energy conformer has the *A*-ring facing the pyridine unit of the 5-membered metallacycle. The *B*-ring faces the metalated naphthalenyl motif.

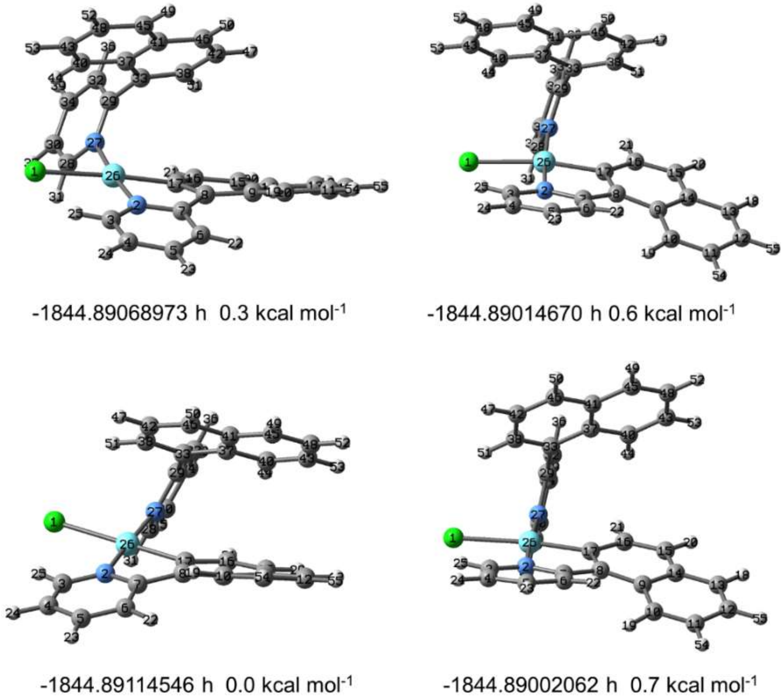

Figure S7. Different conformations of complex **2a** resulting from rotation about the Pt–N bond. All structures were computed at the D95(d);cc-pVDZPP,ECP60MDF/wb97XD/DCM level of theory. Relative energies are given for each conformation.

Rotation about the Pt–N bond shows two energy barriers, one involving the interaction of the aryl moiety with the coordinated Cl^- ion and the second one involves interaction with the aryl moiety belonging to the 5-membered metallacycle. At the same level of theory, these barriers are 29.1 and 25.8 kcal mol^{-1}, respectively. It is worth noting that the larger barrier involves the rotation on the side of coordinated Cl^-. These results are preserved at the cc-pVTZ/cc-pVTZPP;ECP60MDF/wB97XD/DCM level of theory (i.e., using the correlated consistent triple zeta plus polarization basis set an all atoms). The barriers in this case are 29.1 and 25.5 kcal mol^{-1}. Using the same basis set and the meta-hybrid M06 xc-functional (cc-pVTZ;cc-pVTZPP,ECP60MDF/M06/DCM), the results, though smaller, follow the same trend being 25.8 and 24.3 kcal mol^{-1}.

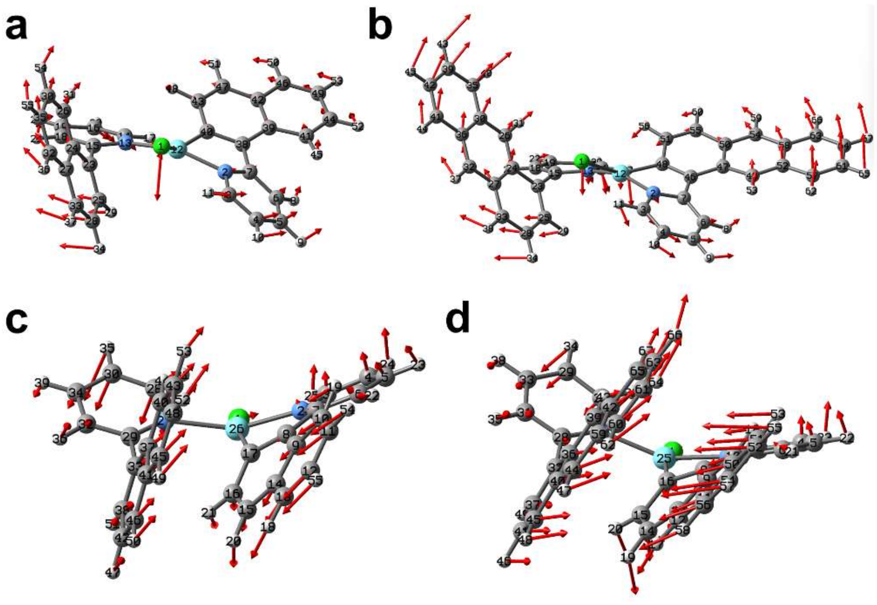

Figure S8. Transition state structures and the corresponding transition vectors for the Pt–N torsions on the coordinated chloro side for (a) naphthalenyl and (b) anthracenyl, and the aryl side for (c) naphthalenyl and (d) anthracenyl. All structures computed at the D95d;ECP60MDF,ccpVDZPP/wb97xd/DCM level of theory.

Similar results were found in the case of anthracenyl (**3a**) substituents. In this case, the barriers are 32.9 kcal mol^{-1} and 25.9 kcal mol^{-1} for the Cl^- and anthracenyl sides, respectively, at the D95(d);ECP60MDF/wB97XD/DCM level of theory.

A large barrier for the Pt–N torsion has been further verified in the case of **1a**. The results indicate barriers of 28.8 and 29.4 kcal mol^{-1}, respectively, at the D95(d);ECP60MDF/wB97XD/DCM level, and confirmed with the larger basis set with both wB97XD (28.7 and 29.0 kcal mol^{-1}), and M06 functionals (26.1 and 27.1 kcal mol^{-1}). In the case of **1a**, the order of the barrier is reversed, but in the same order of magnitude.

It is interesting to note that the barriers are almost insensitive to aryl substitution and are close to 30 kcal mol^{-1}. This is not surprising once the geometries of the transition state (TS) are inspected. On the Cl^- side of the compounds the short contact of the *A*-ring of the aryl substituent is the most relevant feature in their structure. The remaining part of the aryl unit can be bent away from the Cl^- ion reducing the repulsion effect of the larger substituent. The geometry of the aryl-aryl barriers shows that the two aryl moieties tend to slip one over the other adjusting the rotation around the C–C bond and getting in all cases comparable distances between their average planes.

Overall, these results strongly suggest that atropoisomerism does not emerge from rotation in the Pt–N.

C–C rotation barrier

For the rotation about the C–C bond in **2a** and **3a**, two barriers should exist as well. These involve the interaction of the atoms in the Pt coordination plane with *A*- or *B*-rings and the two hydrogen atoms bound to the carbon atoms closest to the rotation axis. These interactions are the main contributors to the energy barriers characterizing the transition states TS(*A*) and TS(*B*),

respectively. For **1a**, only one large TS exists because the C_2 symmetry of the phenyl ring. A second TS exists between the two minima corresponding to two different angles of the Pt–N bond torsion, as observed for **2a** (see above), which is very small in this case.

The barrier for the full C–C rotation in **1a** is only 8.8 kcal mol^{-1} at the d95(d);cc-pVDZPP,ECP60MDF/wB97xd/DCM level of theory. When using the same basis set and the M06 xc-functional, the barrier is a little lower (6.9 kcal mol^{-1}). At the more advanced cc-pTVZ;cc-pVTZPP,ECP60MDF/M06/DCM level of theory, the same barrier is computed at 7.7 kcal mol^{-1}, excluding any possibility to observe atropisomerism.

In case of **2a**, at the 6-31g(d);cc-pVDZPP,ECP60MDF/wb97XD/vac level of theory, the two barriers associated with TS(*A*) and TS(*B*) are 22.2 kcal mol^{-1} and 30.1 kcal mol^{-1}, respectively. The same TS structures, at the D95(d);cc-pVDZPP,ECP60MDF/wb97XD/DCM level of theory, have very similar energies at 22.5 and 30.1 kcal mol^{-1}.

On the other hand, at the D95(d);cc-pVDZPP,ECP60MDF/M06/DCM level of theory, the lowest barrier has and energy of 18.9 kcal mol^{-1}. Increasing the basis set at the cc-pVTZPP on Pt and cc-pVTZ on all the remaining atoms, the barrier is 19.3 kcal mol^{-1} with very similar TS-vector and structure. This energy converges to a value of 19.2 kcal mol^{-1}, with the larger quadruple zeta basis set cc-pVQZPP and cc-pVQZ, in fair agreement with the experimental value. The same trend is observed for **3a.** At the cc-pVTZ;cc-pVTZPP,ECP60MDF/M06/DCM level of theory the lowest barrier is 20.4 kcal mol^{-1}.

It is worth noting that the torsional potential of the free naphthalenylpyridine ligand at the same level of theory has a TS(A) barrier of 8.2 kcal mol^{-1} on account of the interaction between the two hydrogen atoms bound to *C3* position on the pyridine and *C8* position on the naphthalenyl groups.

This suggests that the interaction of the *A*-ring with the atoms in the Pt square planar coordination plane contributes almost equivalently to the energy barrier.

In conclusion, the comparative computational results of the energy barriers of the Pt–N and C–C torsions strongly suggest that the atropoisomerism is due to the rotation around the C–C bond of the arylpyridines and not torsion around the Pt–N bond.

Table S2. Cartesian coordinates of minima and TS's for the atropoisomerization process at the cc pVTZ;cc pVTZPP,CP60MDF/M06/DCM level of theory.

1a min	2a min	3a min
Cl -2.3547439454 1.6665952844 -0.2014522851<br>N 0.9849393851 1.7084965979 -0.7624469622<br>C 0.5822736202 2.8097481909 -1.402682285<br>C 1.4614224739 3.6263261128 -2.07757398<br>C 2.8039134179 3.2877710889 -2.0902360874<br>C 3.219786386 2.1519850569 -1.4291967145<br>C 2.2955041917 1.3618168095 -0.7613606209<br>H 4.2627495781 1.8694178241 -1.42740305<br>H 3.5216944911 3.9055334562 -2.6127005748<br>H 1.0933825316 4.5071538659 -2.5821349542<br>H -0.4798056122 3.0142462259 -1.3556674038<br>Pt -0.2605035152 0.4316815177 0.2258549967<br>N -1.3670601841 -0.9385037725 1.301341498<br>C -1.6720642093 -0.5887746529 2.5602472091<br>C -1.6868929301 -2.1726395202 0.8663867198<br>C -2.2680282222 -1.4496293256 3.4510045336<br>H -1.4045654303 0.4190180328 2.8460778403<br>C -2.2853261656 -3.0848010436 1.7276813304<br>C -2.571078862 -2.7312983551 3.02842369<br>H -2.4822183368 -1.1146843401 4.4548683568<br>H -2.5386210411 -4.065330382 1.349688019<br>H -3.0366147226 -3.440264943 3.6991023916<br>C -1.4019312223 -2.5648612798 -0.5250407927<br>C -0.7289352949 -3.7559089059 -0.7761648479<br>C -1.8412603084 -1.7907901266 -1.5957072168<br>C -0.4775851979 -4.1581442784 -2.0759606562<br>C -1.6014861291 -2.2038085498 -2.8937423342<br>H -2.3813354845 -0.871146653 -1.4047022207<br>C -0.9153874048 -3.3831653598 -3.1367011066<br>H -1.9551965971 -1.6018863296 -3.7203775072<br>H -0.7240919303 -3.6992637096 -4.1538557179<br>C 2.5914968813 0.1472278887 -0.0204914085<br>C 3.8746021476 -0.3700627552 0.1286073845<br>C 1.4784468377 -0.4868901201 0.564981613<br>C 4.076462038 -1.5203946707 0.865828912<br>C 1.7110904744 -1.6391662784 1.3105627433<br>C 2.9913992166 -2.1500052186 1.458487502<br>H 0.8877906261 -2.1559284922 1.7928272011<br>H 3.1448947398 -3.0484441605 2.0440437924<br>H 5.0735569029 -1.9234722194 0.98281669<br>H 4.7229538215 0.1255526588 -0.3284273889<br>H -0.380679115 -4.3584316688 0.054757239<br>H 0.0618481109 -5.0779020467 -2.2593936807	Cl 1.1818228775 18.242198252 7.3342857956<br>N 1.3201010686 15.1632061428 5.9401006247<br>C 1.5981223846 15.9455856919 4.8955069278<br>C 1.7195426885 15.4497764904 3.6175344962<br>C 1.5763280151 14.0867019617 3.4315778033<br>C 1.2849146087 13.2777680711 4.5094078135<br>C 1.1248962539 13.8247576903 5.779803393<br>C 0.9117385137 13.1052002431 7.0279636956<br>C 0.5329850628 11.7325392729 7.1583725229<br>C -0.0716467667 10.9597059834 6.1413743195<br>C -0.4333826104 9.6563672666 6.3504971701<br>C -0.2103330189 9.0371146159 7.5888698022<br>C 0.3279692805 9.7659773879 8.6099949008<br>C 0.6827831113 11.1177825117 8.43306179<br>C 1.1485413532 11.8826199227 9.5224844314<br>C 1.3455210732 13.2255354465 9.4026230753<br>C 1.1823965969 13.8719214783 8.1627000104<br>H 0.4720274293 9.3239029171 9.5896451908<br>H -0.3150732344 11.4136101986 5.1923786374<br>H 1.290114687 11.3876558674 10.4774264942<br>H 1.6339431767 13.8019733461 10.2741237556<br>H 1.2219833777 12.2108376068 4.3724242774<br>H 1.7020114555 13.6490126417 2.4503976151<br>H 1.9431688072 16.1183476584 2.7998492297<br>H 1.7225445884 16.9966215208 5.1229809371<br>Pt 1.2139504537 15.8319440504 7.8527265675<br>N 1.0087174575 16.3691784804 9.8453830008<br>C 2.0320116838 17.0071984942 10.432356624<br>C -0.1141218038 16.1330883134 10.5556825599<br>C 1.9973571414 17.4233990636 11.7424680478<br>H 2.8983225422 17.1775837752 9.8086939748<br>C -0.2055031661 16.5413784459 11.8789438517<br>C -1.2471788264 15.460092442 9.8862342434<br>C 0.8526564953 17.1876343791 12.481812847<br>H 2.8535169133 17.9277849617 12.1649680204<br>H -1.1254174744 16.3555052668 12.4161933628<br>C -1.7218636952 14.1933849845 10.3272059879<br>C -1.8241524723 16.0705009838 8.800752242<br>H 0.7820260791 17.510831128 13.5112224649<br>C -1.198607678 13.5053812477 11.4448766931<br>C -2.7491105298 13.5609370607 9.5746881573<br>C -2.863609464 15.4557744882 8.0878781386<br>C -1.6629177118 12.2704178097 11.7962428095<br>H -0.4045830105 13.9568058801 12.0256707197<br>C -3.1959798354 12.2776251333 9.9552637916<br>C -3.3034167968 14.219192729 8.4582900072<br>H -3.3009906812 15.9640495196 7.2392489193<br>C -2.668677475 11.6428744602 11.0413019068<br>H -3.9721761769 11.807433546 9.3627865244<br>H -4.0907802666 13.722186496 7.9037972064<br>H -1.4625041699 17.0419952041 8.4861030006<br>H -3.020091249 10.6592479789 11.3238522585<br>H -1.2436103072 11.7640927099 12.6561359583<br>H -0.912924954 9.1033862544 5.5529107021<br>H -0.4896958002 8.0025455664 7.737521662	Cl 1.3289414316 18.5864320298 7.5390075862<br>N 1.4378794137 15.5539164865 6.0601160356<br>C 1.6956663608 16.3573402808 5.0154702818<br>C 1.7601850241 15.8846511404 3.7121275349<br>C 1.5722228336 14.5178804788 3.498057262<br>C 1.3001662496 13.6865193187 4.5793412399<br>C 1.2077973463 14.217556398 5.8739372707<br>H 1.2041019365 12.6200142856 4.4235239069<br>H 1.64751524 14.098692641 2.4986076852<br>H 1.9723484348 16.5713766374 2.8999988331<br>H 1.8530227226 17.4038350133 5.2573095173<br>Pt 1.3291105428 16.1676699511 7.9829621967<br>N 0.9798032038 16.5844963517 9.9710876499<br>C 1.9604323225 17.0433245784 10.7703863523<br>C -0.2589349029 16.3820285645 10.4851020124<br>C 1.7648260624 17.3008918094 12.1209130545<br>H 2.925065718 17.1972492538 10.2991338754<br>C -0.5177898573 16.6199590052 11.8348180807<br>C 0.4999238577 17.080928547 12.6671485299<br>H 2.5917047306 17.6655331698 12.7207218279<br>H -1.5204965495 16.4489655894 12.2137045621<br>H 0.3073552162 17.2711284319 13.718931137<br>C -1.3272367142 15.9173519686 9.5529006387<br>C -1.8004793065 14.5551453998 9.5920997464<br>C -1.7996671593 16.7881171288 8.6046681227<br>C -1.4001593239 13.6356016232 10.5679449613<br>C -2.6948447288 14.1171281706 8.5538910882<br>C -2.7318573708 16.3613719432 7.6116903015<br>H -1.4273938784 17.8087607209 8.5781690793<br>C -1.8243142177 12.3019521724 10.5365747811<br>H -0.724069669 13.9457938655 11.3606001152<br>C -3.1085235538 12.7800551245 8.5155517119<br>C -3.1463058889 15.05875858 7.5725203134<br>H -3.0807185052 17.076530893 6.872070726<br>C -1.4014356763 11.3557701097 11.5263359563<br>C -2.6845238506 11.857140651 9.4785858139<br>H -3.7732695378 12.4527762492 7.7177082601<br>H -3.8296670866 14.7182565007 6.7975832845<br>C -1.8102922248 10.0504260286 11.4653897614<br>H -0.7447774128 11.693415363 12.3257313226<br>C -3.0871694393 10.4817738205 9.4433478795<br>C -2.6653322742 9.60613157 10.4066720584<br>H -1.4823485823 9.3388050471 12.2190546686<br>H -3.7263104316 10.1432809155 8.6305171249<br>H -2.9698815646 8.5633564947 10.3686254703<br>C 1.0392802863 13.4677125245 7.124981453<br>C 0.6542102204 12.0770120266 7.2455326121<br>C 1.3475147536 14.2179671191 8.2572513825<br>C -0.0532430189 11.3604312297 6.2725012917<br>C 0.9139224571 11.423188264 8.5027809226<br>C 1.607026088 13.5342389033 9.4907715357<br>C -0.4547758823 10.0294597919 6.4749830403<br>H -0.3778888718 11.8482754456 5.3587141151<br>C 0.5543850469 10.0875762052 8.6901172383<br>C 1.4672798498 12.1802259965 9.5866426794<br>H 1.9390258739 14.100164113 10.3580362991<br>C -1.2093367382 9.3132087377 5.4888626862<br>C -0.1213332642 9.365065819 7.6990142797<br>H 0.7585082852 9.6151075503 9.649276569<br>H 1.6965127842 11.6637909893 10.5167566514<br>C -1.594142582 8.0157739814 5.7071823762<br>H -1.4707192256 9.8161345594 4.55980612<br>C -0.5357176744 8.0083932918 7.893500247<br>C -1.2502138185 7.3505971404 6.9271083303<br>H -2.1646877911 7.4810984975 4.9517512443<br>H -0.2813781186 7.5126138832 8.8282208491<br>H -1.5649865999 6.3215576274 7.0811564583
**1a TS**	**2a TS**	**3a TS**
Cl -2.6312099389 0.8556799162 -0.3371836392<br>N 0.5560252281 1.8861734131 -0.791328881<br>C -0.1393036826 2.8604017944 -1.3851418405<br>C 0.4757084724 3.9396816358 -1.9781434183<br>C 1.8582664101 4.0110902576 -1.9540624986<br>C 2.5756480486 3.0058034112 -1.3419671279<br>C 1.9108648027 1.9374460791 -0.7580005451<br>H 3.6552282842 3.0418889547 -1.3122889131<br>H 2.373348426 4.8457547861 -2.4098714536<br>H -0.1242450912 4.7053035127 -2.4468938658<br>H -1.2152929348 2.7425601444 -1.3669236057	Cl 0.4049680049 18.1763577846 7.3187977883<br>N 1.0426810857 15.3128534797 5.6424206128<br>C 1.1184648998 16.2424816194 4.6876893925<br>C 1.4018915653 15.9299310742 3.3783389456<br>C 1.6558715028 14.6074493268 3.0656802371<br>C 1.5710481179 13.6464281559 4.0501770447<br>C 1.2215106394 13.9955466167 5.3517893799<br>C 1.1473183493 13.1197566338 6.5134063809<br>C 1.1348258189 11.6883252218 6.483213343<br>C 0.8368296514 10.9013279007 5.3476749552<br>C 0.8492537656 9.5339277152 5.3985527424	Cl 0.4410902811 18.2107294477 7.3263022219<br>N 1.0706998185 15.3516392959 5.6414650074<br>C 1.1623171712 16.2834979534 4.6898650567<br>C 1.4462991227 15.9714560312 3.3803017027<br>C 1.6832571113 14.6462326993 3.0640704794<br>C 1.5814544562 13.6828238354 4.0446471906<br>C 1.2344226467 14.0317075607 5.3476364586<br>C 1.14647714 13.1548213154 6.50418699<br>C 1.1212394454 11.7144619579 6.4657197058<br>C 0.81845536 10.9466886849 5.3456504529<br>C 0.8134468763 9.5526028396 5.3720367585

Pt	-0.2822666929	0.2692416819	0.1214302567
N	-1.0079101672	-1.3421526418	1.2406711872
C	-1.1338169182	-0.9426800457	2.5214960788
C	-1.4170596674	-2.5861955613	0.8920317146
C	-1.6796729618	-1.7101280694	3.5169992979
H	-0.7719512929	0.0516294367	2.7422510486
C	-1.9949899312	-3.3911163201	1.8790726989
C	-2.1331081788	-2.9695304495	3.1780992697
H	-1.7472680505	-1.3192018396	4.5209263918
H	-2.3499586702	-4.3749488124	1.6263852412
H	-2.5855755831	-3.6198131632	3.9139723975
C	-1.2578498151	-3.1375606167	-0.4816260428
C	-0.7195749101	-2.4184251572	-1.5474619411
C	-1.6445988959	-4.4574114318	-0.7530411961
C	-0.5798581552	-2.9725457987	-2.8069944694
C	-1.5085531408	-5.0128780746	-2.0089840421
H	-2.0596792404	-5.0904174776	0.0153409761
C	-0.9750883442	-4.2734822046	-3.0504298224
H	-1.8226786748	-6.0355879765	-2.1703729877
H	-0.8690473221	-4.7077926262	-4.0356831827
C	2.5353796208	0.8104764929	-0.0865665126
C	3.9117364017	0.6533919814	0.0430490127
C	1.6439725575	-0.1492022704	0.4294625955
C	4.423793775	-0.4590814874	0.6821415424
C	2.1841593103	-1.2640391468	1.0626574119
C	3.5567062986	-1.4169938231	1.1879981974
H	1.5322062741	-2.0315034074	1.4651721812
H	3.9546689741	-2.2936985994	1.6845063696
H	5.4937350967	-0.5830101456	0.7831395694
H	4.5900157405	1.3976825313	-0.3570997421
H	-0.3979528692	-1.3958583956	-1.4043091049
H	-0.1581555894	-2.3719784704	-3.6022606079

C	1.1592253443	8.8603013204	6.5881951902
C	1.3925088743	9.5856131695	7.7204007491
C	1.3589129522	10.9938728176	7.7038631583
C	1.505030148	11.7253891874	8.9004193216
C	1.3573377622	13.0795461157	8.9201187642
C	1.1487418754	13.8047709446	7.7292126894
H	1.5875435546	9.0901881797	8.6630810207
H	0.5339459334	11.3729305408	4.427062939
H	1.7004092749	11.1807912621	9.8161168931
H	1.4280451566	13.600272782	9.8656676663
H	1.8182801909	12.6261184643	3.8156456565
H	1.930561373	14.3225568677	2.0602494077
H	1.4486005581	16.7125744072	2.6377239359
H	0.9532662033	17.2596365347	5.0147502722
Pt	0.8239616435	15.7671440979	7.6014392137
N	0.7769794723	16.08719188	9.6902249649
C	1.960552901	16.6135235466	10.0395270926
C	-0.0465216495	15.5819842771	10.6525512431
C	2.482999015	16.5567195719	11.308295098
H	2.522548739	17.0670541391	9.2364957032
C	0.5032878024	15.3989798643	11.9242229542
C	-1.4331987042	15.1708166687	10.3139838604
C	1.7551315291	15.8662129145	12.2543405912
H	3.4478072429	16.990700354	11.5167176946
H	-0.0369020809	14.8475449192	12.6660585024
C	-2.3459797272	14.502649785	11.2178940776
C	-1.8974284101	15.4043879877	9.0369881099
H	2.1457237772	15.6972775952	13.2472688358
C	-2.3189793863	14.5531428075	12.6321058824
C	-3.4595220378	13.8120615678	10.6511486277
C	-3.0512428695	14.8127763273	8.5143037462
C	-3.201992717	13.8510762011	13.4065159415
H	-1.652499442	15.2253560471	13.1419224887
C	-4.3228208152	13.054754104	11.4704577818
C	-3.7685948707	13.9518253206	9.2836693287
H	-3.3318731274	15.0158839024	7.491099555
C	-4.187014407	13.043566403	12.8260631571
H	-5.1264722198	12.5110864157	10.9910134363
H	-4.6210370142	13.4167017084	8.8865234484
H	-1.3396419272	16.0564422179	8.3842071575
H	-4.8609697688	12.4701910539	13.4459355772
H	-3.1490261896	13.9461650192	14.4820211226
H	0.5993195603	8.9679089938	4.5118431765
H	1.1799997674	7.7801518902	6.6115501578

C	1.1237614743	8.8677351714	6.5836940965
C	1.366734441	9.6254704068	7.7224241452
C	1.3461568598	11.0124654932	7.6981490077
C	1.4936597259	11.7515717853	8.8997939266
C	1.3492868439	13.0996732061	8.9170158238
C	1.1471594214	13.831525711	7.7181530711
H	1.5499845933	9.1216119943	8.6661125804
H	0.5220894442	11.417857433	4.4192022321
H	1.6879485707	11.2048255783	9.8158766012
H	1.4246299082	13.6243860173	9.8613975601
H	1.8143733446	12.6581701011	3.808244901
H	1.9572611503	14.3605860762	2.0573963418
H	1.5057012476	16.7565168895	2.6415937812
H	1.0075489576	17.3023260756	5.0209998873
Pt	0.8455216305	15.798587692	7.60202857
N	0.7883082769	16.1087389548	9.6897975614
C	1.9638696702	16.6467214753	10.0505622088
C	-0.0406465602	15.5974522242	10.6457712471
C	2.4729892057	16.5994938726	11.3252350129
H	2.5301509318	17.1024220702	9.2502816
C	0.4965470111	15.4278431665	11.9252308655
C	-1.4135803681	15.1610806625	10.2966916939
C	1.7405436891	15.9072549768	12.2670410108
H	3.4329378649	17.0426531425	11.5420441317
H	-0.0506166785	14.8745956327	12.6628565677
C	-2.3269982854	14.4813458257	11.2080886787
C	-1.8698581596	15.3647135607	9.0184530727
H	2.1217603915	15.7467641915	13.2662386727
C	-2.3282555866	14.5708692612	12.5971840142
C	-3.4130765978	13.7327573453	10.6261040134
C	-3.0030052415	14.7262760303	8.4819061735
C	-3.2126392302	13.8569815913	13.4059400114
H	-1.6831886794	15.2669658762	13.1093262129
C	-4.2646287397	12.9823173845	11.4268529657
C	-3.6985545223	13.8489080057	9.2425950725
H	-3.2674309973	14.9058845733	7.44884565
C	-4.17622707	12.9944430822	12.8110461803
H	-5.0563894644	12.4137394833	10.9503681837
H	-4.5240125591	13.2770876203	8.8362780418
H	-1.3166301037	16.0159467613	8.358228905
C	1.1321307292	7.4491711617	6.591680569
C	0.841491622	6.746673511	5.4652053917
H	1.3720018838	6.9396379055	7.5178082147
C	0.5191963813	7.4246032521	4.2642493832
H	0.8504177857	5.664673399	5.4796887722
C	0.5028575074	8.783319099	4.2205923791
H	0.2854583974	6.8505823055	3.3769850998
H	0.2582722168	9.303751604	3.301751537
C	-3.1809941453	13.9653643098	14.8201446302
C	-4.0313862864	13.2377642819	15.5918835312
H	-2.4561647131	14.6317640745	15.2731943688
C	-4.9741028337	12.3633907851	14.99793482
H	-3.9919200032	13.3221463212	16.6700258819
C	-5.0473205043	12.2485717973	13.6461031358
H	-5.6409416909	11.7921806186	15.6303307012
H	-5.7742289412	11.5929302469	13.1813273173

First oxidation test on 2a

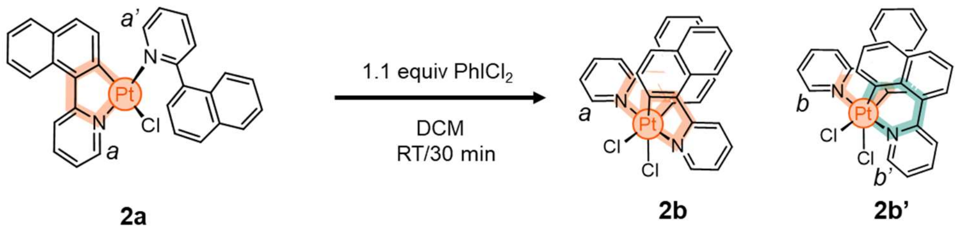

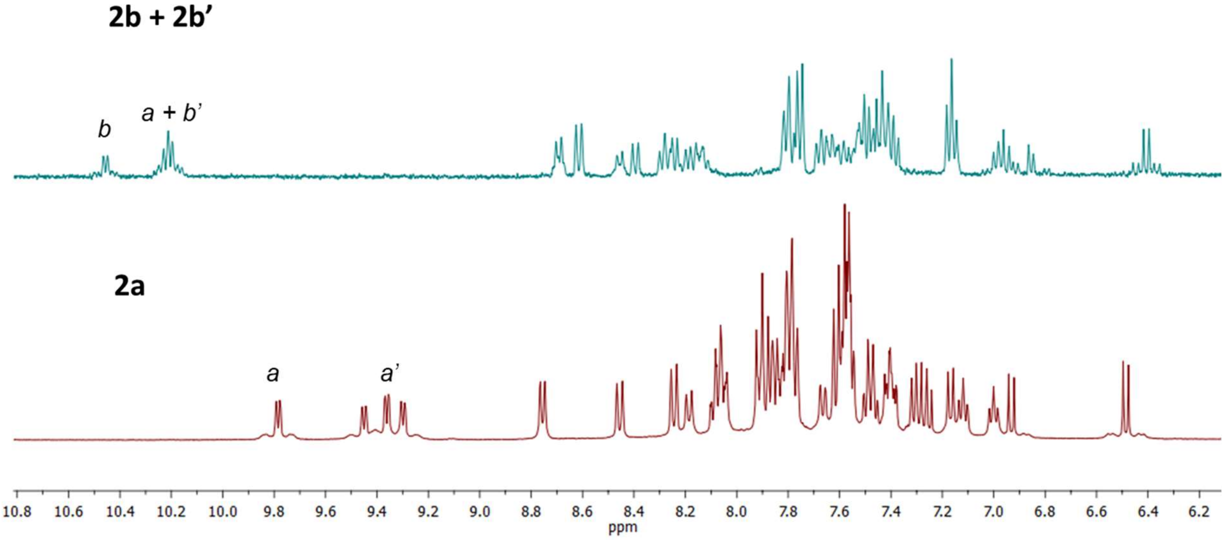

Figure S9. ^{1}H NMR spectra (400 MHz, DCM-d_2) of **2a** and the crude reaction product obtained from its oxidation with 1.1 equiv of $PhICl_2$ at room temperature for 30 min.

First oxidation test on 3a

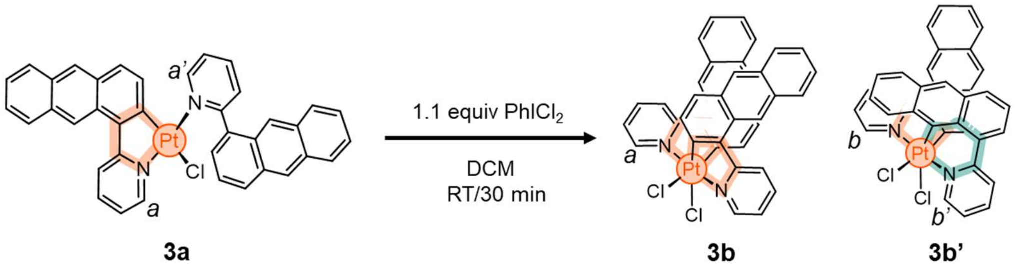

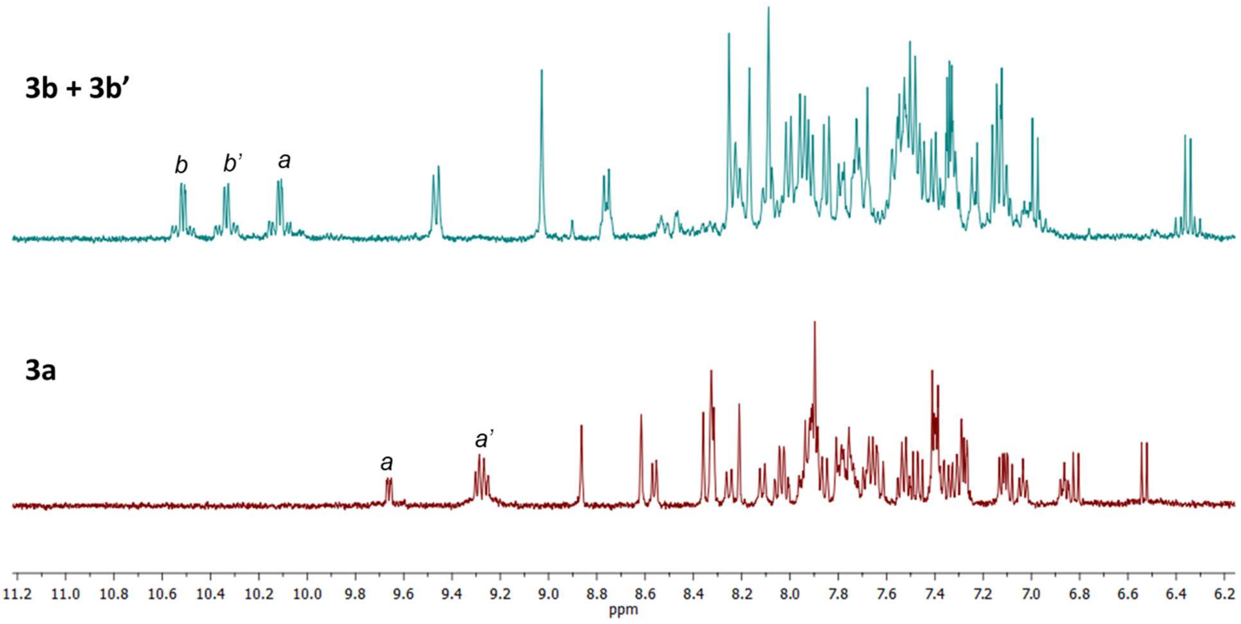

Figure S10. ^{1}H NMR spectra (400 MHz, DCM-d_2) of **3a** and the reaction crude product resulting from its oxidation with 1.1 equiv of $PhICl_2$ at room temperature for 30 min.

Computed relative stability of isomers 2b/2b’ and 3b/3b’

Table S3. Energy difference (kcal mol^{-1}) compared to the most stable isomer computed at the 6 31g(d);cc pVDZPP,ECP60MDF/xc-functional/DCM level of theory with the indicated xc functional.

xc-functional	2b	2b’
wB97XD	0.0	4.1
B2PLYPD3	0.0	3.4
M06	0.0	3.0
	**3b**	**3b’**
wB97XD	0.0	8.1

Table S4. Cartesian coordinate at the 6 31g(d);cc pVDZPP,ECP60MDF/wB97XD/DCM level of theory.

2b	3b
Pt 7.4724021852 7.7102073995 16.0784238581	Pt 0.0000000088 0.0000000167 1.5959426427
Cl 7.0486015061 9.4344615304 17.7931166968	Cl -1.5380735592 -0.8641910382 3.3202574496
N 9.4840463052 7.6074979331 16.3329440338	N 1.1896291626 -1.6418980697 1.4936568677
C 7.8998580008 6.2797546061 14.7432577115	C 1.2656434272 0.6000297045 0.1642425969
C 11.4825605844 8.0972664682 17.5353454167	C 1.6787176275 -3.9214649141 1.9878490788
H 11.9772436918 8.6792237669 18.3047032222	H 1.4581571106 -4.8060600294 2.5745122937
C 11.3742449049 4.2307458579 11.9630083564	C 5.6532611277 -0.0555488144 -1.9153848259
C 10.1364920445 8.3045961913 17.2767308899	C 0.9506385555 -2.7603395294 2.1964719415
H 9.53941878 9.018707994 17.8345604463	H 0.1396566583 -2.7032259812 2.9151216891
C 10.3804674157 3.4113377798 11.3353553798	C 5.4312205834 1.0993024192 -2.7338968889
C 12.143385186 7.0991437567 16.8185846366	C 2.6454629793 -3.9187482121 0.9818916197
H 13.1815474138 6.8614244405 17.0308336921	H 3.1963273966 -4.8233905978 0.7419213325
C 6.9182136461 5.5549971642 14.0076436499	C 1.1334906306 1.8213665166 -0.5577226739
H 5.860573191 5.7217957539 14.1846819757	H 0.2858500905 2.4767270685 -0.3846949739
C 10.1216710338 6.6898839314 15.5516589937	C 2.190325999 -1.5770404735 0.5699192583
C 7.3052144604 4.6164470718 13.0941565504	C 2.0606415922 2.1601469539 -1.5015390833
H 6.5586692028 4.0321151149 12.5607934889	H 1.940626206 3.0712645114 -2.0835506623
C 12.7279437877 4.1492077542 11.4977956216	C 6.9133399642 -0.7333223458 -2.0082495535
H 13.4836888366 4.7763142259 11.9660372345	H 7.0891463382 -1.6057842562 -1.3824558303
C 10.770900955 2.5227029083 10.2804112021	C 6.4624661937 1.5287127823 -3.6322747505
H 10.0124881009 1.9007417697 9.8098138463	H 6.2870374742 2.4047251126 -4.2529680056
C 9.6922118943 5.1777858742 13.4776608681	C 3.4066527644 0.1493931645 -0.9502964228
C 9.2506336718 6.032998898 14.5646886661	C 2.3152233639 -0.2672140428 -0.0882209243
C 11.4622431329 6.3955611755 15.8306044367	C 2.8982558688 -2.7486208454 0.2733552752
H 11.9613054691 5.588931415 15.3122561751	H 3.6115348117 -2.7629031536 -0.5387254045
C 9.0501006068 3.5428125152 11.7494106811	C 4.2246178571 1.7941837164 -2.5925563137
H 8.2728880404 2.9738728982 11.2421179796	H 4.0636733623 2.7084608009 -3.1612052124
C 11.0096590137 5.0919823347 13.0100204648	C 4.6447979065 -0.4984321428 -1.0449656021
H 11.7818610776 5.7410641723 13.4063750605	H 4.8839609183 -1.3330071959 -0.3963359457
C 8.6871283299 4.4189300159 12.7775354558	C 3.2308390418 1.36209302 -1.7081604163
C 12.0760026958 2.4617942262 9.8699647821	C 7.6493985305 0.8494031413 -3.7040347954
H 12.3682640886 1.7867781389 9.0698162417	H 8.4287533591 1.1777802889 -4.3867271012

C	13.0678677377	3.290350712	10.4861405347	C	7.8784286395	-0.2966307223	-2.8768923838
H	14.0975027176	3.2315612821	10.1428369182	H	8.8288834235	-0.8196348632	-2.9445810535
Cl	7.8962045101	9.4344481358	14.3637187357	Cl	1.5380734949	0.8641911485	3.32025745
N	5.4607581386	7.6074981294	15.8239045433	N	-1.1896291696	1.6418981025	1.493656886
C	7.0449450406	6.2797632508	17.4135997401	C	-1.2656433947	-0.6000296817	0.1642425938
C	3.4622440505	8.0972645557	14.6215021188	C	-1.6787177067	3.9214649333	1.9878490995
H	2.9675613171	8.6792198016	13.8521425107	H	-1.4581572115	4.8060600559	2.5745123113
C	3.5705525149	4.2307497749	20.1938393282	C	-5.6532611093	0.0555487882	-1.9153848021
C	4.8083128881	8.3045938045	14.8801154605	C	-0.9506385903	2.7603395737	2.1964719527
H	5.4053866337	9.0187033001	14.322283479	H	-0.1396566844	2.7032260557	2.9151216904
C	4.5643294282	3.4113435006	20.8214957302	C	-5.4312205497	-1.0993024328	-2.7338968792
C	2.8014186568	7.0991449129	15.3382665991	C	-2.6454630747	3.9187481992	0.9818916572
H	1.7632559806	6.8614262844	15.1260189754	H	-3.19632753	4.8233905643	0.7419213789
C	8.0265883908	5.5550080992	18.1492175127	C	-1.1334905809	-1.8213664872	-0.5577226811
H	9.0842290855	5.7218065708	17.9721804996	H	-0.2858500278	-2.4767270262	-0.3846949861
C	4.8231326819	6.689887708	16.6051929608	C	-2.1903260165	1.5770404773	0.5699192891
C	7.6395863386	4.616459003	19.0627052981	C	-2.0606415418	-2.1601469354	-1.501539088
H	8.3861309152	4.0321285205	19.5960709219	H	-1.9406261428	-3.0712644875	-2.083550673
C	2.2168518282	4.1492076595	20.6590460526	C	-6.9133399548	0.7333223043	-2.0082495207
H	1.4611070204	4.7763119854	20.1908011957	H	-7.0891463399	1.6057842039	-1.3824557856
C	4.1738935184	2.522706315	21.8764371445	C	-6.4624661544	-1.528712797	-3.6322747468
H	4.9323058418	1.9007463207	22.3470368643	H	-6.2870374239	-2.4047251171	-4.2529680132
C	5.2525893031	5.1777935778	18.6791935786	C	-3.406652742	-0.14939317	-0.9502964067
C	5.694169151	6.0330057714	17.5921659216	C	-2.315223347	0.2672140489	-0.0882209102
C	3.4825603284	6.3955650381	16.3262489491	C	-2.8982559317	2.748620824	0.2733553174
H	2.9834972465	5.5889379856	16.8446007826	H	-3.6115348869	2.7629031068	-0.5387253522
C	5.8946975726	3.542821274	20.4074456105	C	-4.2246178149	-1.7941837169	-2.5925563123
H	6.6719093049	2.9738825949	20.9147406354	H	-4.0636733099	-2.7084607939	-3.1612052201
C	3.9351404974	5.0919872319	19.1468288571	C	-4.6447978936	0.4984321205	-1.0449655746
H	3.1629382901	5.7410664018	18.7504700505	H	-4.8839609152	1.3330071621	-0.3963359068
C	6.2576719102	4.4189398575	19.3793224571	C	-3.2308390034	-1.3620930166	-1.7081604123
C	2.8687901315	2.4617938012	22.2868777237	C	-7.6493984995	-0.8494031696	-3.7040347839
H	2.5765268438	1.7867756614	23.0870238446	H	-8.4287533232	-1.1777803177	-4.3867270949
C	1.8769257011	3.2903485851	21.6706986161	C	-7.8784286238	0.2966306803	-2.8768923574
H	0.8472892302	3.2315555094	22.0139971337	H	-8.8288834144	0.8196348101	-2.9445810208
**2b’**				**3b’**			
Pt	3.3292405702	0.766844395	2.7081234341	Pt	0.4379767637	14.8785247036	8.6342521004
Cl	4.0313493912	-0.4195875466	0.6477457893	Cl	-1.0059555058	16.7037160314	9.508538828
Cl	4.2429646152	-0.9876068981	4.1941125645	Cl	2.21042423	16.4777165675	8.0158985621
N	5.130114847	1.7336117333	2.9144801336	N	1.1332814193	14.7120049925	10.5614679735
N	1.519633755	-0.1075482987	2.4271381029	N	-0.1484684671	15.0038770495	6.6954503785
C	0.6032197105	0.651389611	1.7656096587	C	-0.7767409884	16.0762368194	6.1902064783
C	6.2252183649	1.0343483056	2.5428975638	C	-1.0883185796	16.1724765691	4.8443991595
C	2.4607335598	2.1511702824	1.5491187246	C	-0.6974735347	15.1305113374	4.0017627598
C	3.1315050191	3.2330226781	0.9329905528	C	-0.0149172431	14.0396092747	4.5277806322
C	1.0883761106	1.9888134466	1.3921874257	C	0.2546768648	13.9750191896	5.9005111724
C	5.2621477001	2.9766811669	3.4371384444	C	1.1159984324	13.0017072089	6.5828630575
C	2.5975720064	1.7775774003	4.3032591127	C	1.6057554352	11.7557876511	6.032293644
C	1.5997983523	1.2196799404	5.0757891608	C	0.9998805885	11.0694209677	4.9739612656
C	2.0742984273	3.9651638963	5.2847031349	C	1.509343612	9.8531698344	4.4926832652
C	4.0957463192	3.768098294	3.9083130347	C	2.6820302416	9.2870373832	5.0918844573
C	6.5491497166	3.5129770253	3.6018156633	C	3.2000828853	8.0522320767	4.5801501598
C	1.269482673	-1.3847169142	2.7564471558	C	2.5808430938	7.4158038227	3.537310713
C	4.2128739944	5.1482452768	3.9279224573	C	1.4020135712	7.9734890978	2.9465894095
C	0.0667674385	-1.9963023024	2.4422053366	C	0.8813731812	9.1525257564	3.4111378992
C	0.3007324411	3.0484999088	0.8194718386	C	3.2603203873	9.9432312866	6.1857185178
C	0.834849917	2.0046346088	5.9752300115	C	2.7355916203	11.1413539336	6.6798211484
C	2.2553606947	5.374947622	5.310998264	C	3.2838095741	11.7423528965	7.8592657804
C	1.0279166922	3.3627678722	6.0353912071	C	2.6803876247	12.8175508435	8.4474677699
C	0.2587784624	5.1942533273	-0.397044474	C	1.5519441372	13.4222139656	7.8259705448
C	-0.8773457652	-1.261709634	1.7231820488	C	1.588888223	15.8455364635	11.1394531265
C	-1.1197165177	3.1295776544	0.894744461	C	2.0103850138	15.89573796	12.4558657646

C	3.2709159423	5.9620247985	4.5947461757	C	1.9251230212	14.7345572739	13.2230401942
C	7.508885678	1.5374762793	2.6518007743	C	1.4508849134	13.5749593701	12.626406213
C	2.4136117333	4.1750289665	0.2386010457	C	1.0808494372	13.562749214	11.2727202964
C	2.9378335412	3.151019897	4.4887548192	C	0.6214055811	12.2934209033	10.6616347368
C	-0.6041948292	0.0570585926	1.3779047812	C	1.1717875335	11.1265043397	11.1449250771
C	7.6748442533	2.80842661	3.1994192414	C	0.6866417525	9.8494051022	10.7567421088
C	0.9917107113	4.1371148485	0.2087620407	C	-0.4096421164	9.7691322318	9.9456283771
C	-1.1166669009	5.2137568354	-0.3567538198	C	-1.0164211886	10.9514792019	9.4144545715
C	-1.8062877468	4.1807283773	0.3249204422	C	-2.1344765152	10.8441188053	8.5865691903
H	0.8075642709	6.0020160315	-0.8764244463	C	-2.7503151414	11.984887489	8.0735454476
H	-1.6718746638	6.0285237331	-0.8135494592	C	-3.957632819	11.8726863648	7.3054406825
H	-2.8884741423	4.2256276079	0.4159449609	C	-4.5798561049	12.9815785522	6.8048315748
H	-1.6804574031	2.3986451218	1.4658159854	C	-4.0304585882	14.2746082239	7.0671625586
H	-1.3066799715	0.6032956404	0.7638518293	C	-2.864789342	14.4124506561	7.7702840286
H	-1.8145699651	-1.7166458861	1.4163737193	C	-2.1496673039	13.277864997	8.2856865186
H	-0.101822442	-3.0279996922	2.7295702585	C	-0.9222867499	13.3747989349	8.9885040007
H	2.0675202851	-1.9031243828	3.2778997116	C	-0.4462593877	12.2452811607	9.6877294232
H	1.3525088866	0.1669542292	4.9824101972	H	4.0919861305	7.627941245	5.0363628389
H	0.0580488035	1.5280974112	6.567610423	H	2.9781194179	6.4801589046	3.152472772
H	0.3927051821	3.9917091971	6.6547484639	H	0.9222476742	7.4516462455	2.1225535634
H	1.5672300165	5.9813718948	5.8957250489	H	-0.0133659932	9.5761070745	2.9597782214
H	3.3879819418	7.0416835249	4.5774899714	H	4.1204933424	9.4979884057	6.6827819888
H	5.0475491853	5.6327599044	3.4301848265	H	0.0743922576	11.433367749	4.5395660628
H	6.6653341427	4.474655721	4.0854515647	H	4.1671964421	11.2969008588	8.3111498529
H	8.6639954267	3.2364701501	3.3324445986	H	3.0824009071	13.2310772828	9.36698374
H	8.3480632719	0.9305762961	2.3309092393	H	0.3578478446	13.271902848	3.8636848634
H	6.0431153338	0.0458881551	2.1419075714	H	-0.8988288349	15.1775834688	2.9355473079
H	4.2134826392	3.3067557007	0.9755576438	H	-1.6045577821	17.0510945373	4.4745441333
H	2.9290743817	4.9905656946	-0.2640963971	H	-1.0250425008	16.859215693	6.8990025739
				H	2.208501043	14.7341832271	14.2714136902
				H	2.3705445812	16.8335875515	12.8637146524
				H	1.609971652	16.7248977647	10.5098948073
				H	1.3326134853	12.6741270061	13.2159718401
				H	2.0051391663	11.1669769564	11.840124425
				H	1.1655881264	8.9540898423	11.1420797829
				H	-0.8380823067	8.8046953869	9.6825089786
				H	-2.5374503686	9.8601642212	8.354917486
				H	-4.3747623822	10.8809520711	7.1436693696
				H	-5.4987951107	12.8895286612	6.2315214962
				H	-4.5544704138	15.1607231607	6.7175625789
				H	-2.50175971	15.4039796542	8.0108240061

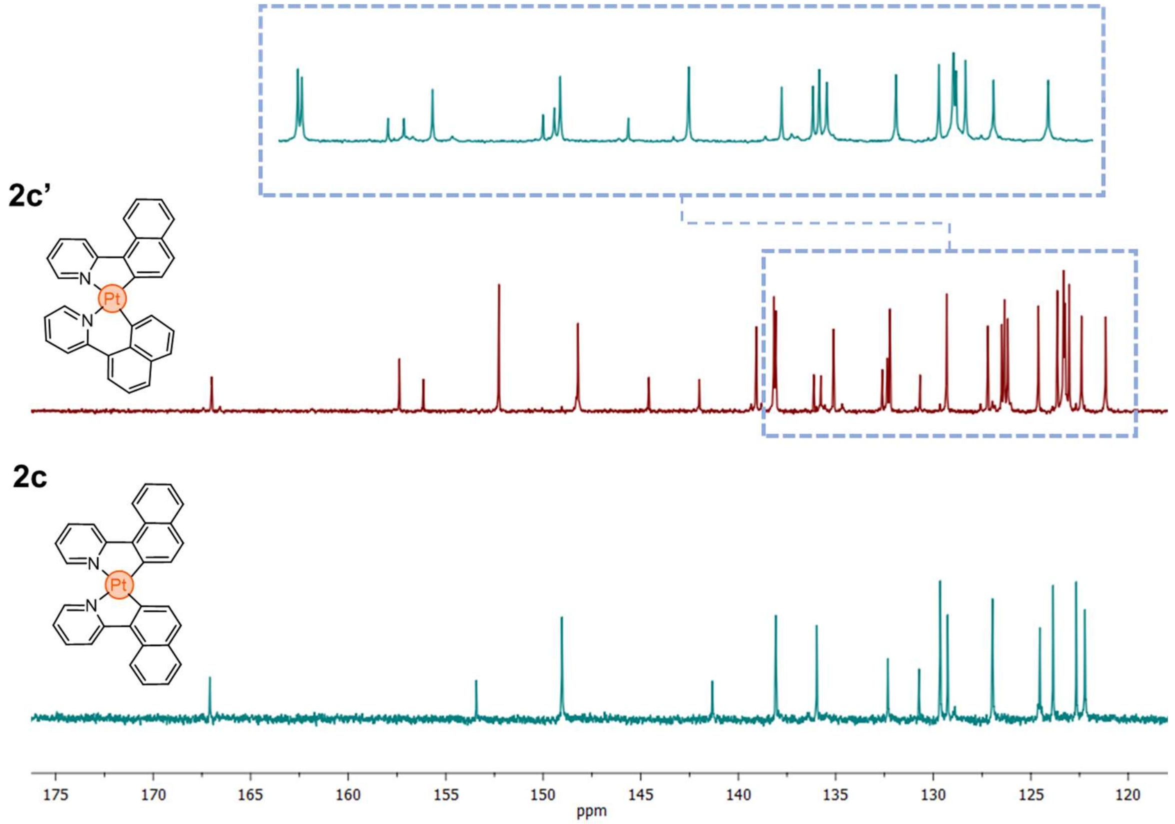

Figure S11. Comparison of the $^{13}C\{^1H\}$ NMR (101 MHz, DCM-d_2) of **2c** and **2c'**.

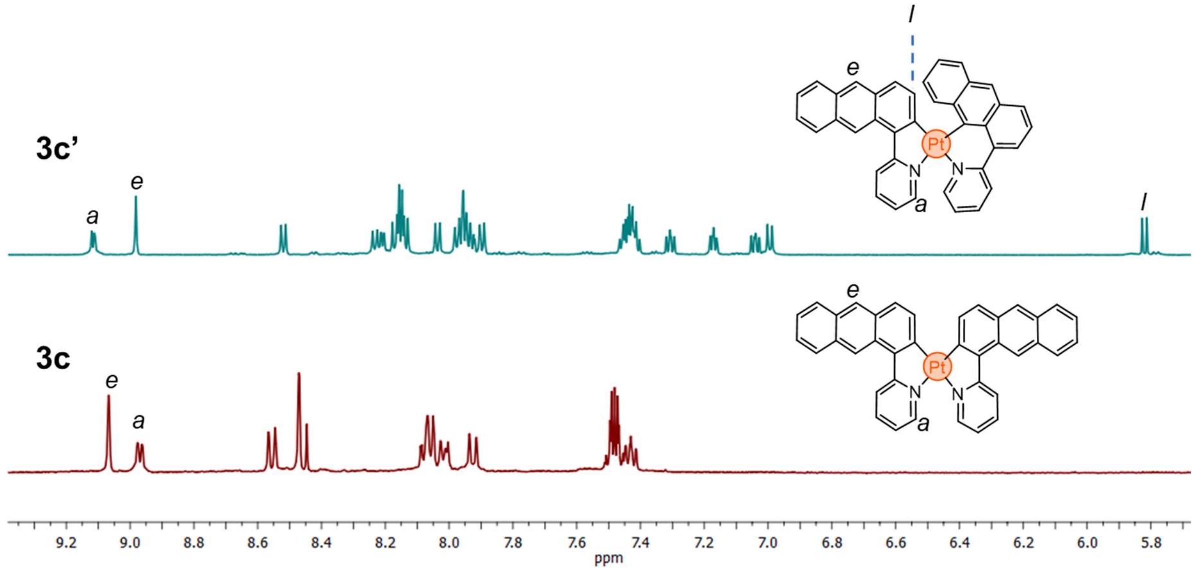

Figure S12. Comparison of the ^1H NMR spectra of **3c** (400 MHz, DCM-d_2) and **3c'** (600 MHz, DCM-d_2).

DFT-minimized structures of Pt^{II} complexes 2c’ and 3c’

Table S5. Cartesian coordinates of **2c’** and **3c’** computed at the 6-31g(d);cc pVDZPP,ECP6MDF/wB97xD/DCM level of theory

2c’				3c’			
Pt	0.83429400	0.75583100	-1.02132300	Pt	-0.1242464584	0.3609733767	0.1416804639
N	0.04169800	2.60384200	-0.32159100	N	-0.5707633506	2.4321039979	-0.1839451048
N	2.87245500	1.32265400	-1.01507200	N	1.2684086834	0.0786682678	-1.4446437499
C	-1.13637500	0.37317600	-1.06947100	C	-1.4076869599	0.7426396444	1.6460179998
C	-3.95565500	0.16006400	-1.48565400	C	-3.4326088714	1.3554652512	3.5658755161
C	-3.35709900	1.44428100	-1.33373000	C	-3.2476261223	2.2135544326	2.4223168057
C	-4.16702500	2.57820400	-1.62418600	C	-4.2241780943	3.1866123951	2.1885724908
H	-3.72174000	3.56588600	-1.65354400	H	-4.2044137359	3.7762388373	1.2737633385
C	-1.31253300	2.69278900	-0.40657500	C	-1.5460720949	2.9260791147	0.6327197665
C	-1.78593300	-0.88783800	-1.20518500	C	-1.629711901	-0.0735868895	2.7983628802
H	-1.18995300	-1.79218500	-1.16486200	H	-1.0205803175	-0.9647240189	2.9376860703
C	-1.96256700	1.50522100	-0.98820400	C	-2.1173567545	1.9475514047	1.5581659307
C	-3.14223600	-0.99563000	-1.35470600	C	-2.5550420512	0.2488998886	3.7458278867
H	-3.61222200	-1.97477700	-1.41708500	H	-2.6746684921	-0.3645889492	4.6400530569
C	3.20659000	2.51861900	-1.53755100	C	0.9839891332	0.5451392969	-2.6741071949
H	2.39436400	3.07701900	-1.98931400	H	-0.0212301641	0.943226801	-2.804661944
C	-1.96682800	3.76762700	0.20860500	C	-1.7867952329	4.3074238683	0.6388139841
H	-3.04674500	3.80392100	0.23090700	H	-2.4920505984	4.7307517944	1.344944336
C	0.73933700	3.55391200	0.31550100	C	0.1141461452	3.253596059	-0.988178631
H	1.81123500	3.40384300	0.37165000	H	0.8920542108	2.7914937149	-1.5940045407
C	-1.23733700	4.75913800	0.84700800	C	-1.0822764324	5.1479195178	-0.2075842178
H	-1.75203100	5.58787700	1.32237300	H	-1.2752806697	6.2186267541	-0.1891123877
C	0.14902100	4.66263600	0.89655300	C	-0.1162662882	4.6165726005	-1.0552049503
H	0.75976700	5.40459900	1.39673000	H	0.4665265889	5.2380506769	-1.728818158
C	3.82850900	0.53795800	-0.47443500	C	2.4740100272	-0.4820439232	-1.1964310841
C	5.15250300	0.99917000	-0.42330900	C	-5.2996607949	3.3984482761	3.0615552422
H	5.92926300	0.35526800	-0.03201100	C	3.4335069954	-0.5222959562	-2.219401281
C	-5.33591600	0.05392400	-1.79453400	H	4.3881266684	-1.0075987757	-2.0409104388
H	-5.77248100	-0.93813600	-1.88105400	C	-4.4848188562	1.5802293039	4.4511570233
C	5.48389000	2.24908600	-0.91788000	H	-4.5963180475	0.9219703473	5.3151571628
H	6.51025400	2.59798500	-0.87440700	C	-7.3222420261	4.5902510472	3.6852858377
C	4.49057900	3.02633600	-1.50555000	H	-8.0678045781	5.3575991638	3.4834299662
H	4.70088300	3.99661900	-1.93905600	C	-5.4215249086	2.5944879735	4.2416860056
C	4.35103500	-2.87193700	1.02247300	C	-7.4334873883	3.8004912181	4.8620212496
C	3.60561700	-3.65210500	0.17891400	H	-8.2621824679	3.9726059802	5.5467081216
C	2.72495700	-3.06360300	-0.76803600	C	-6.2885862022	4.3925440236	2.8114189143
C	2.56462100	-1.64508300	-0.80474100	H	-6.1995253026	4.9987920769	1.9092748911
C	3.52081500	-0.85897300	-0.06736200	C	3.1541626536	0.0009512198	-3.4695136943
C	4.34519500	-1.47172600	0.85588200	H	3.904268684	-0.0373187648	-4.256326728
C	1.99491500	-3.86991400	-1.67923800	C	1.8923513561	0.5343105262	-3.7148809051
H	2.14351100	-4.94658300	-1.66614600	H	1.6113864642	0.9239221458	-4.6890023724
H	4.99672400	-3.32115100	1.77030500	C	-6.5094113959	2.8288544718	5.1295706464
H	3.66971900	-4.73636400	0.22728300	H	-6.5901210515	2.212456625	6.0254292847
H	5.03314600	-0.87678300	1.44867200	C	4.391572051	-2.2736292787	1.4973264897
C	0.85908100	-1.89889600	-2.47937200	C	3.4350127623	-3.0982033527	2.0153441113
H	0.06888000	-1.49607000	-3.10726800	C	2.0659427332	-2.9549809284	1.645537456
C	1.13327000	-3.28269400	-2.56861600	C	1.6729923922	-1.8706455208	0.7794917295
H	0.60305500	-3.88778600	-3.29943600	C	2.7344987828	-1.1659434706	0.0895157312
C	1.48294800	-1.06907000	-1.56032200	C	4.0417286179	-1.3486174534	0.4852006052
C	-6.10166100	1.17227200	-2.00796000	C	1.0978455796	-3.8282382875	2.1396957975
H	-7.15548600	1.08130400	-2.25278000	C	0.3042892499	-1.5511265956	0.6313047743
C	-5.49613900	2.44630900	-1.94923600	C	-0.6460073829	-2.5243766106	1.0311361019
H	-6.08090000	3.33156600	-2.18188800	C	-0.2439683559	-3.6831548905	1.7839573088

	C -1.230916033 -4.6292256539 2.1887512628<br>H -0.9071673583 -5.497515071 2.7642379696<br>C -2.5456701279 -4.4620787483 1.8558154456<br>C -2.9509531414 -3.3278280536 1.1032897846<br>C -2.0312831042 -2.3911742312 0.7179115965<br>H 1.3985375107 -4.6446312368 2.7989420519<br>H 5.4320665537 -2.3673535414 1.8016025358<br>H 3.697465873 -3.8814354688 2.7273020693<br>H 4.8395476893 -0.7984070188 -0.011035563<br>H -3.2870001244 -5.1983152024 2.1633480423<br>H -3.9993582422 -3.2072693108 0.8349578199<br>H -2.3359268748 -1.517653909 0.1409993955

Photophysical properties

UV-vis spectra

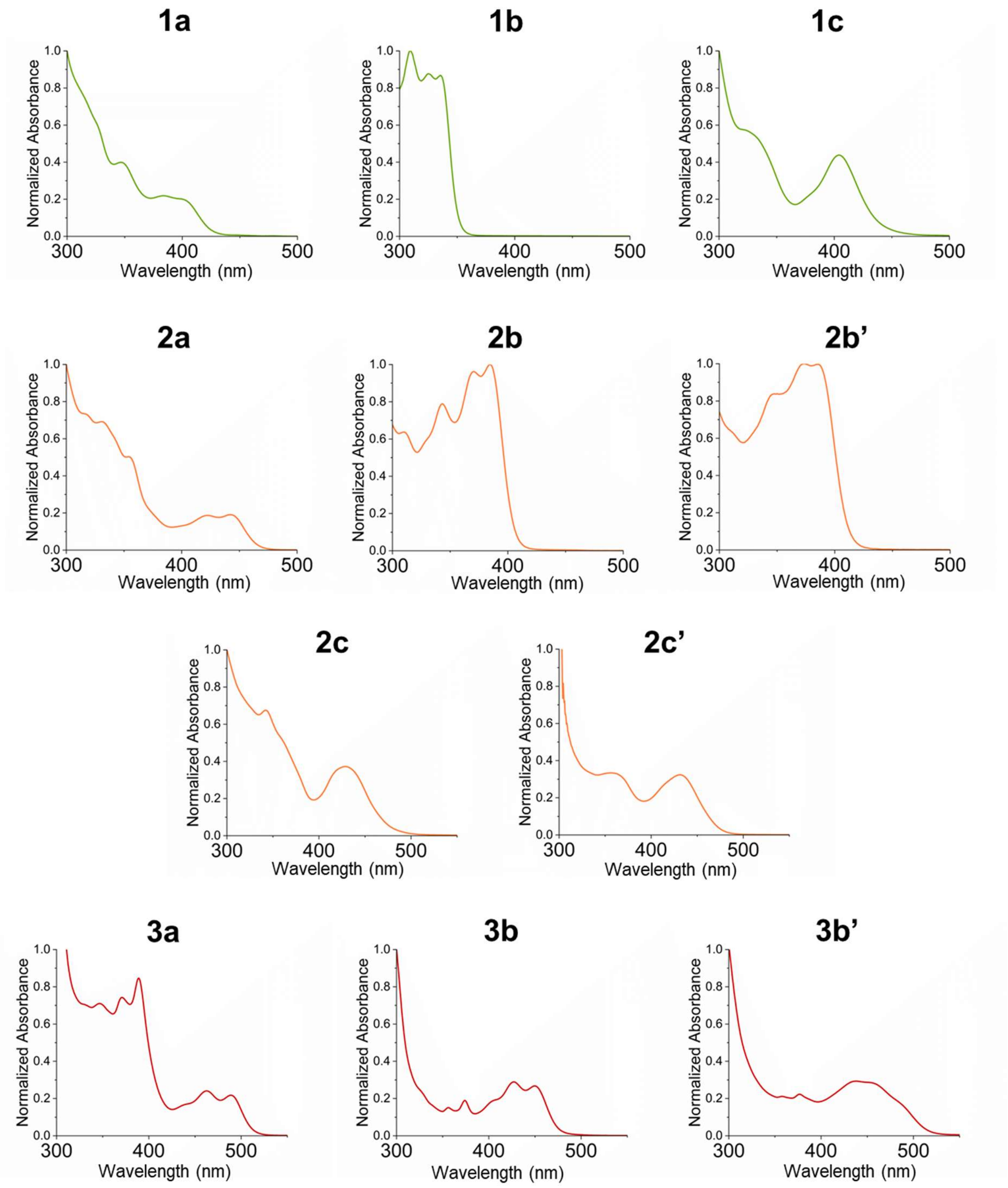

Figure S13 . UV-vis spectra of all complexes in DCM (concentration = 1.0 × 10^{-5} M).

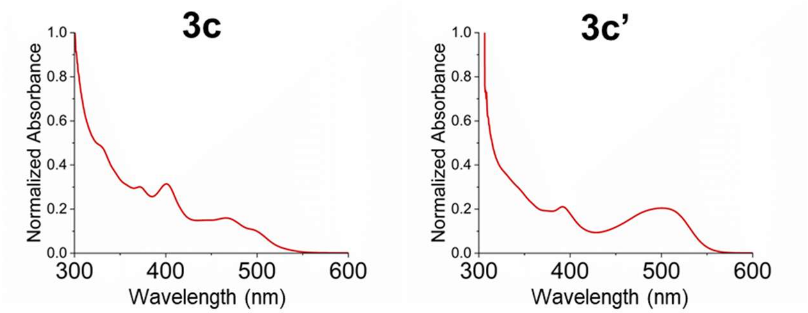

Figure S13 (continued). UV-vis spectra of all complexes in DCM (concentration = 1.0×10^{-5} M).

Photoluminescence spectra

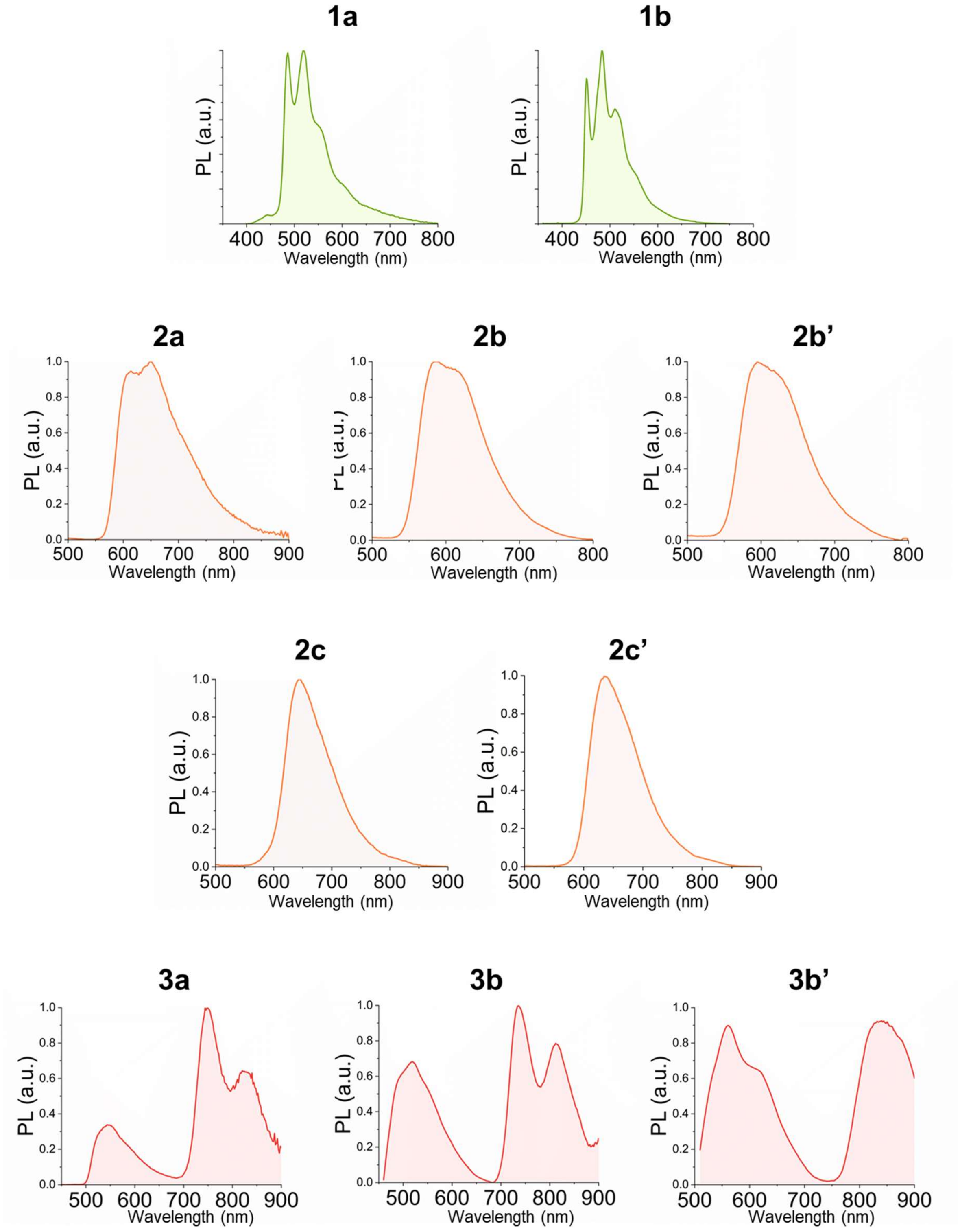

Figure S14. Photoluminescence spectra in DCM (concentration = 1.0 × 10^{-6} M). All solutions were prepared in degassed DCM. Complex **1c** is not emissive in solution.[15]

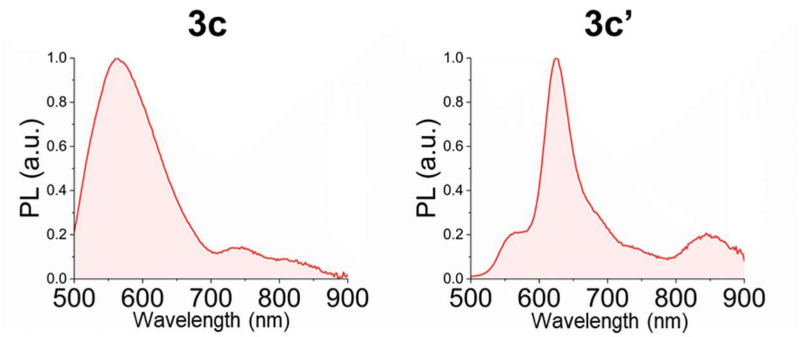

Figure S14 (continued). Photoluminescence spectra in DCM (concentration = 1.0 × 10^{-6} M). All solutions were prepared in degassed DCM. Complex **1c** is not emissive in solution.[15]

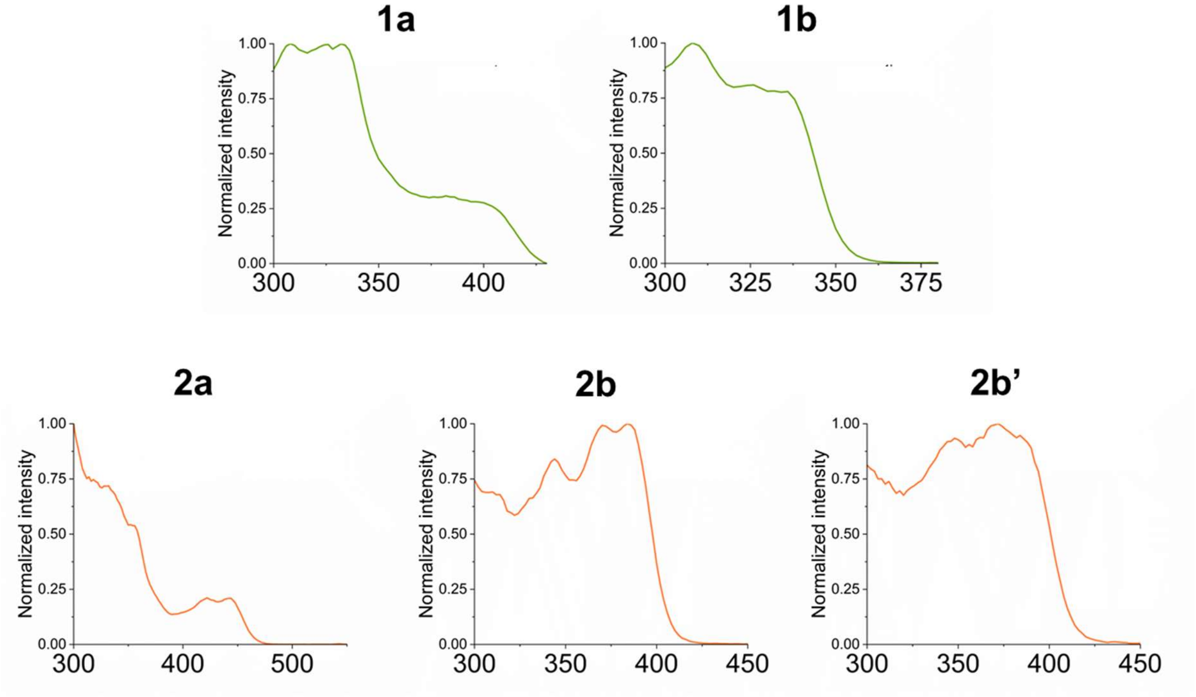

Figure S15. Representative excitation spectra in DCM (concentration = 1.0 × 10^{-6} M). All solutions were prepared in degassed DCM. Emission wavelength (**1a** = 520 nm, **1b** = 450 nm, **2a** = 650 nm, **2b** = 630 nm, **2b'** = 630 nm).

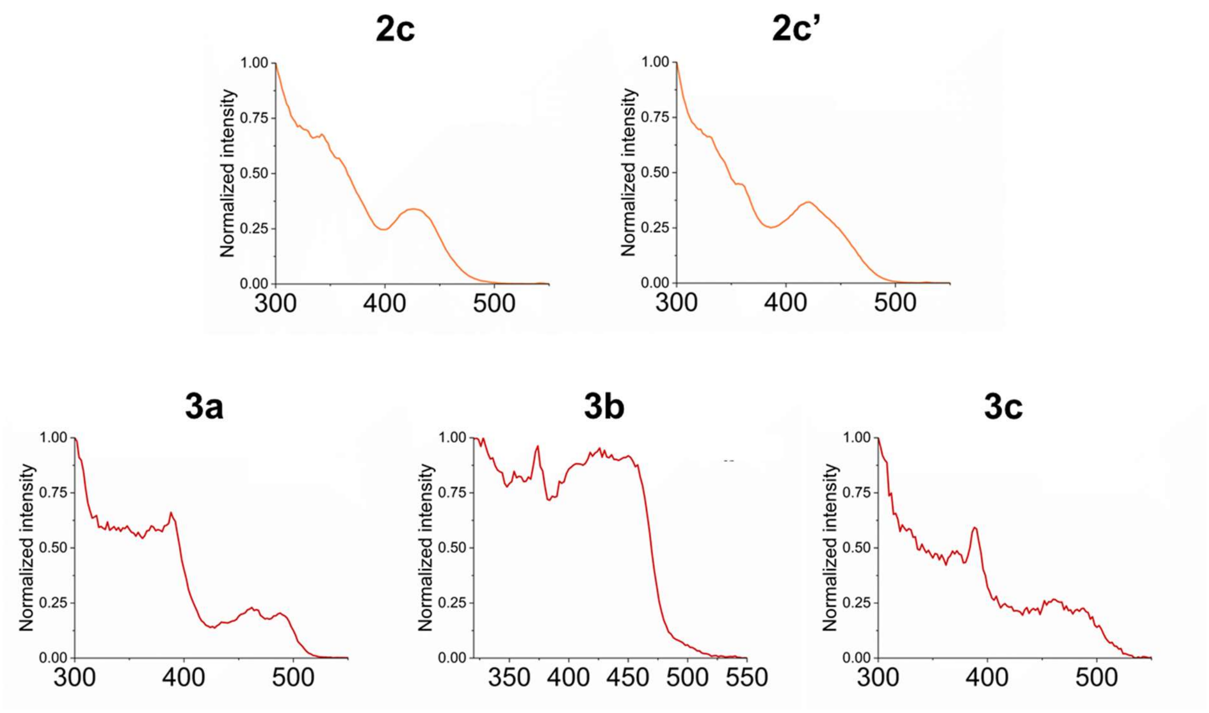

Figure S15 (continued). Representative excitation spectra in DCM (concentration = 1.0 × 10^{-6} M). All solutions were prepared in degassed DCM. Emission wavelength (**2c** = 650 nm, **2c'** = 630 nm, **3a** = 750 nm, **3b** = 750 nm, **3c** = 730 nm).

Lifetime measurements

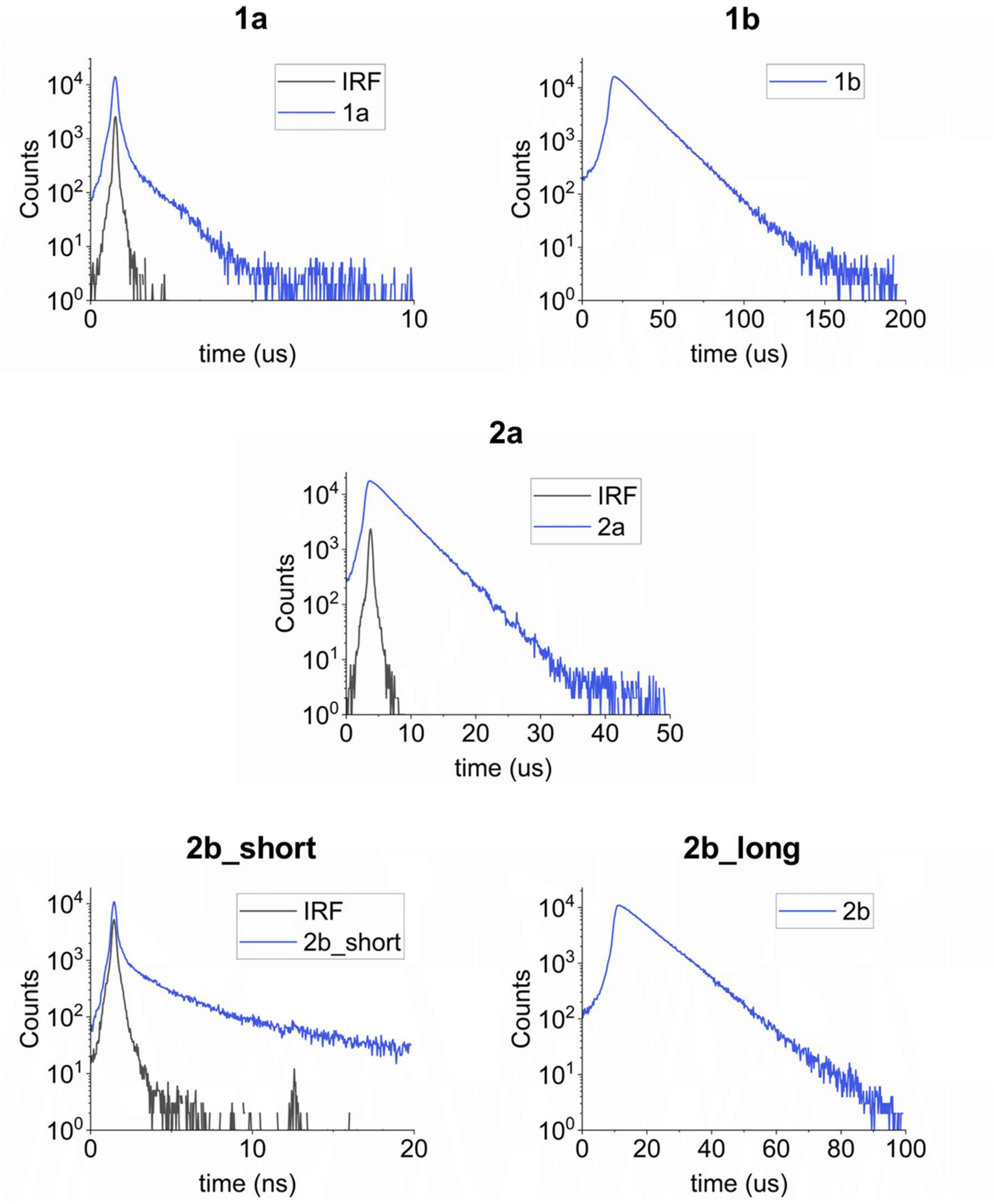

Figure S16. Photoluminescence decays in DCM under inert conditions (Measured using streak camera, λ_{ex} = 355 nm). [Short]: ns time scale fluorescence, [Long]: μs time scale phosphorescence.

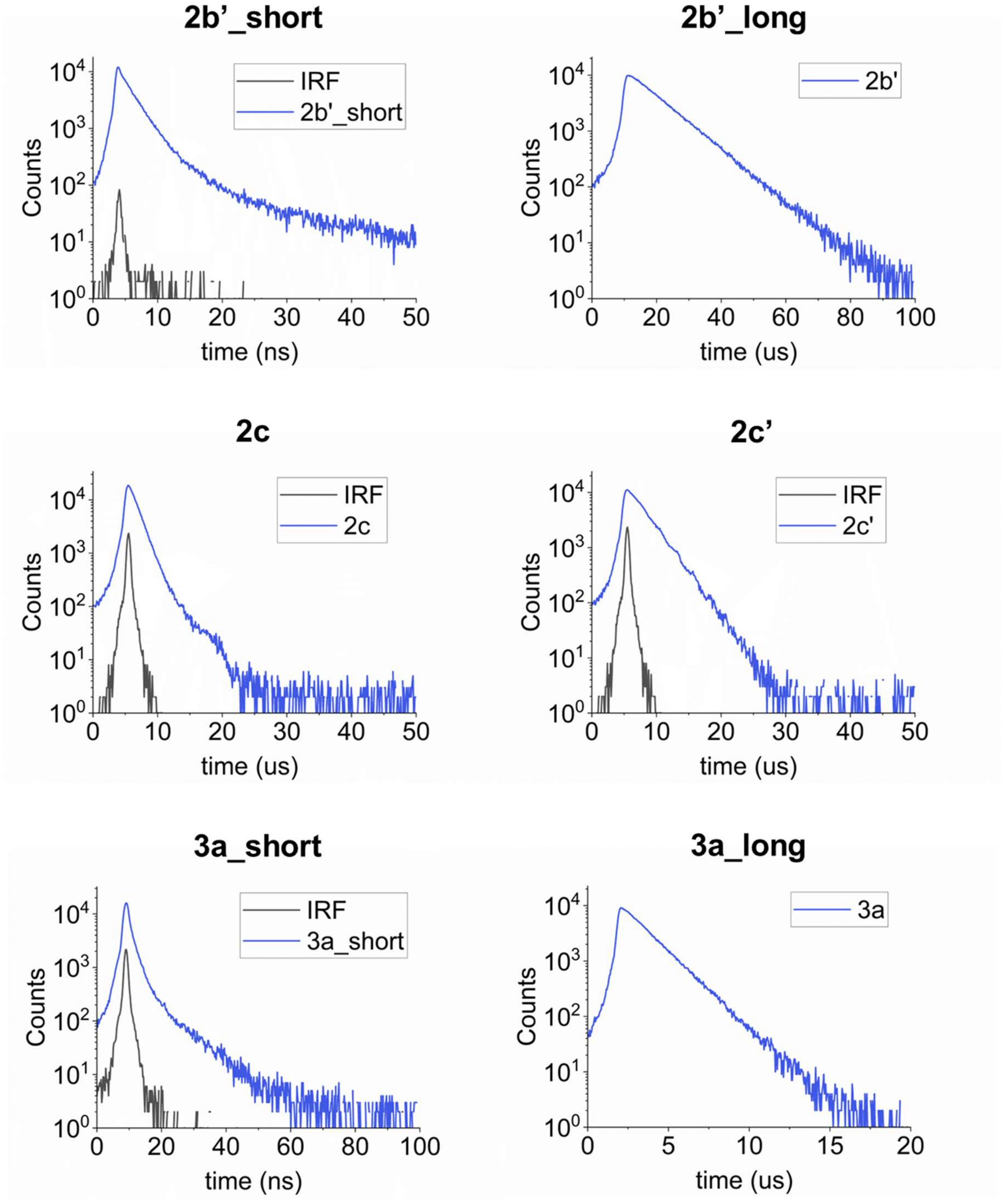

Figure S16 (continued). Photoluminescence decays in DCM under inert conditions (Measured using streak camera, λ_{ex} = 355 nm). [Short]: ns time scale fluorescence, [Long]: μs time scale phosphorescence. IRF = instrument response function.

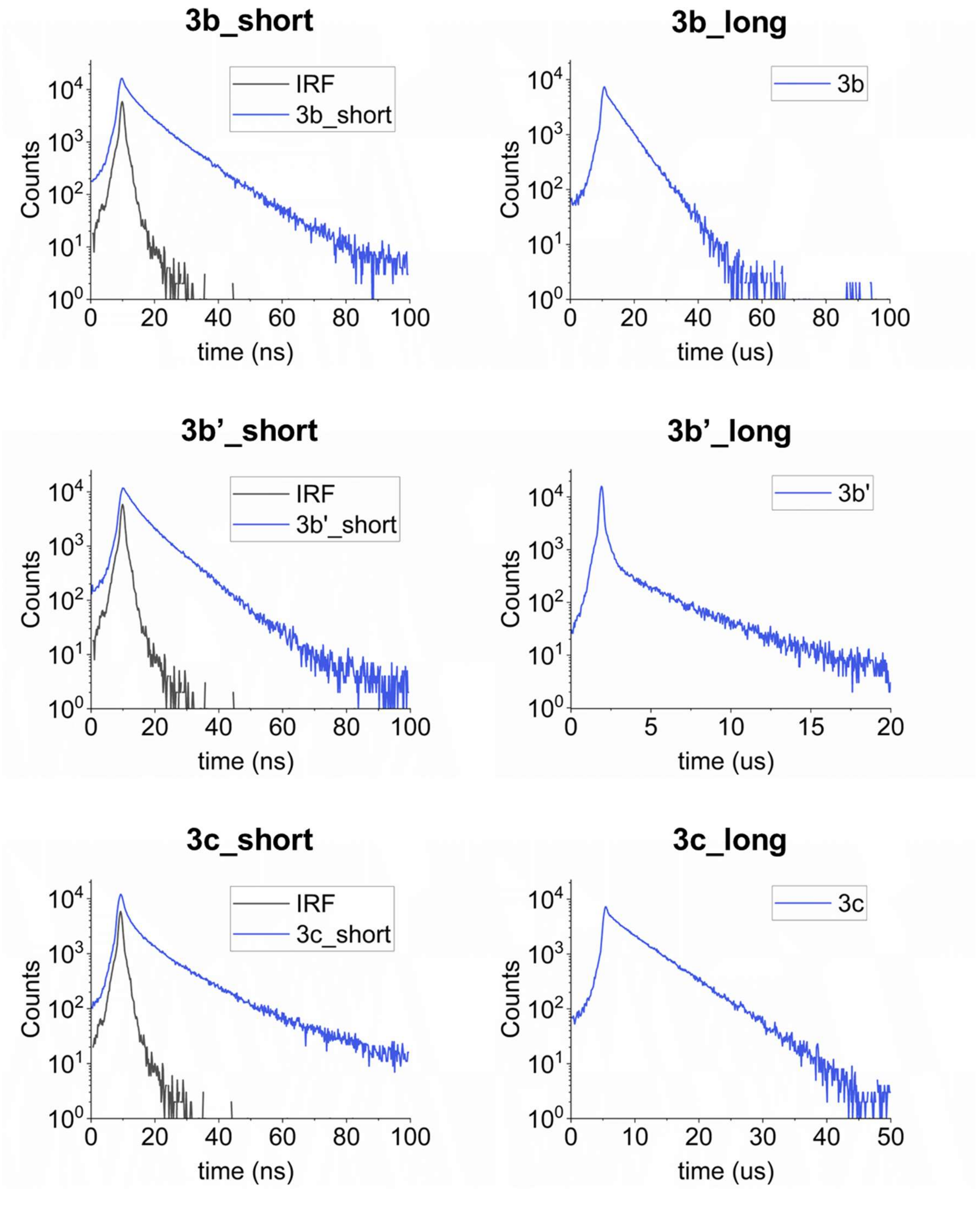

Figure S16 (continued). Photoluminescence decays in DCM under inert conditions (Measured using streak camera, λ_{ex} = 355 nm). [Short]: ns time scale fluorescence, [Long]: μs time scale phosphorescence. IRF = instrument response function.

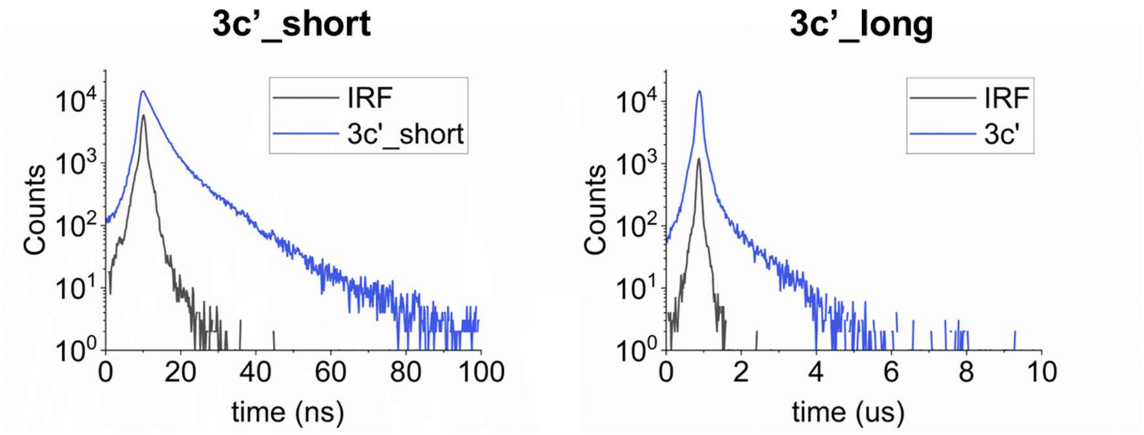

Figure S16 (continued). Photoluminescence decays in DCM under inert conditions (Measured using streak camera, λ_{ex} = 355 nm). [Short]: ns time scale fluorescence, [Long]: μs time scale phosphorescence. IRF = instrument response function.

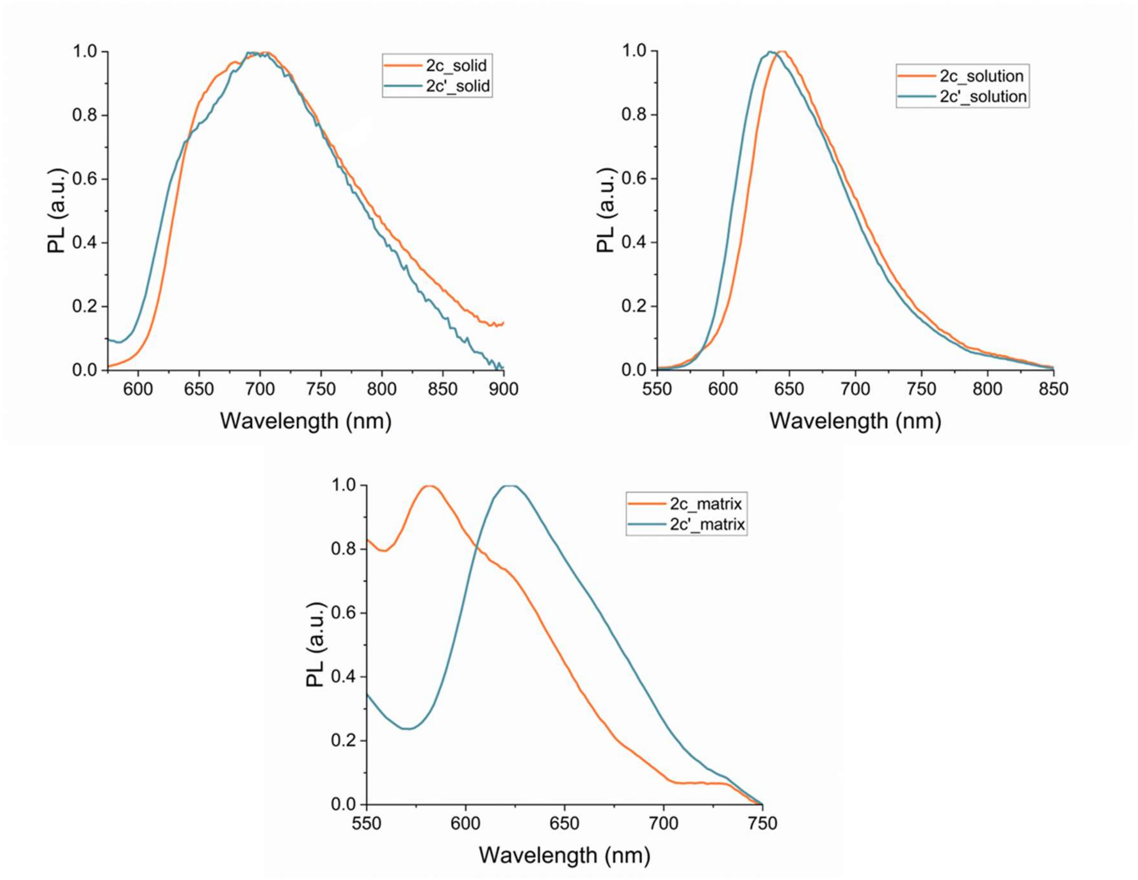

Figure S17. Photoluminescence spectra of **2c** and **2c**’ in the solid state, solution, and a glassy matrix.

Table S6. Summary of the photophysical properties for the analyzed Pt complexes.

Compound	Lowest-energy absorption maxima (nm)	Emission wavelength (nm)	Life time τ		Relative quantum yield (%)	
			Short component (ns)	Long component (μs)	λ_{ex}	Φ
**1a**	384, 405	486, 520, 556, 606	-	0.11 ± 0.02	366	0.16 ± 0.01
**1b**	325, 336	451, 484, 513, 556	-	15.03 ± 0.06	350	1.62 ± 0.01
**1c***	404	-	-	-	-	-
**2a**	421, 443	612, 650, 718	0.44 ± 0.03	3.75 ± 0.01	366	2.34 ± 0.05
**2b**	371, 385	587, 621	0.170 ± 0.003, 2.53 ± 0.25	9.60 ± 0.03	366	1.65 ± 0.09
**2b'**	374,386	595, 629	2.27 ± 0.01	9.58 ± 0.03	366	0.67 ± 0.05
**2c**	429	645	-	1.48 ± 0.02	450	0.69 ± 0.07
**2c'**	431	635	-	2.97 ± 0.02	436	1.35 ± 0.19
**3a**	462 ,489	748, 826	1.38 ± 0.04	1.52 ± 0.01	450	0.107 ± 0.002
**3b**	426 ,450	736, 814	5.93 ± 0.41	4.55 ± 0.08	450	0.092 ± 0.003
**3b'**	436, 457 ,489	840	5.76 ± 0.28	1.33 ± 0.04	450	0.019 ± 0.001
**3c**	467 ,500	742, 830	4.91 ± 0.60	4.87 ± 0.02	450	0.033 ± 0.001
**3c'**	502	848	4.14 ± 0.21	0.09 ± 0.01	450	0.036 ± 0.001

* **1c** is non-emissive in solution

Synthesis and characterization

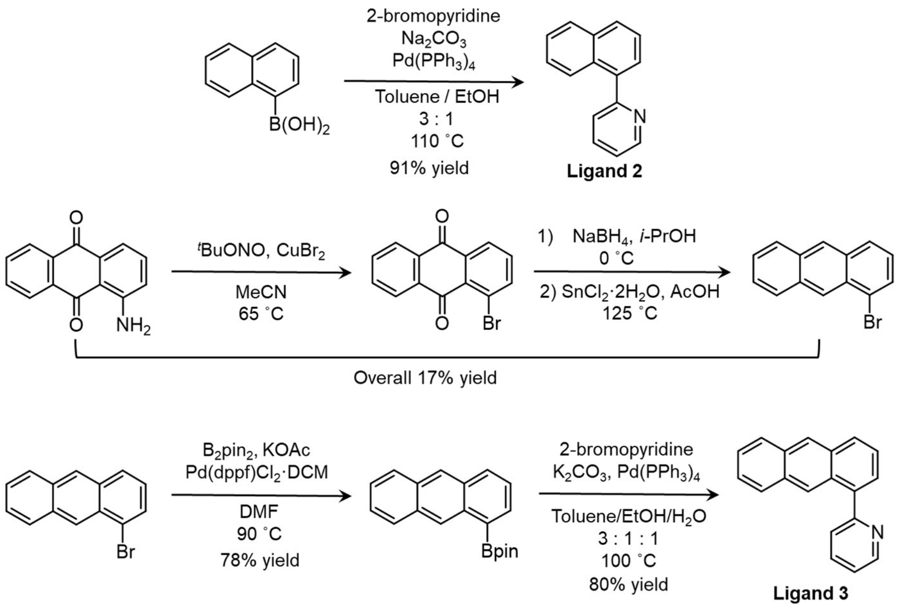

Scheme S1. Synthesis of Ligand **2** and **3**

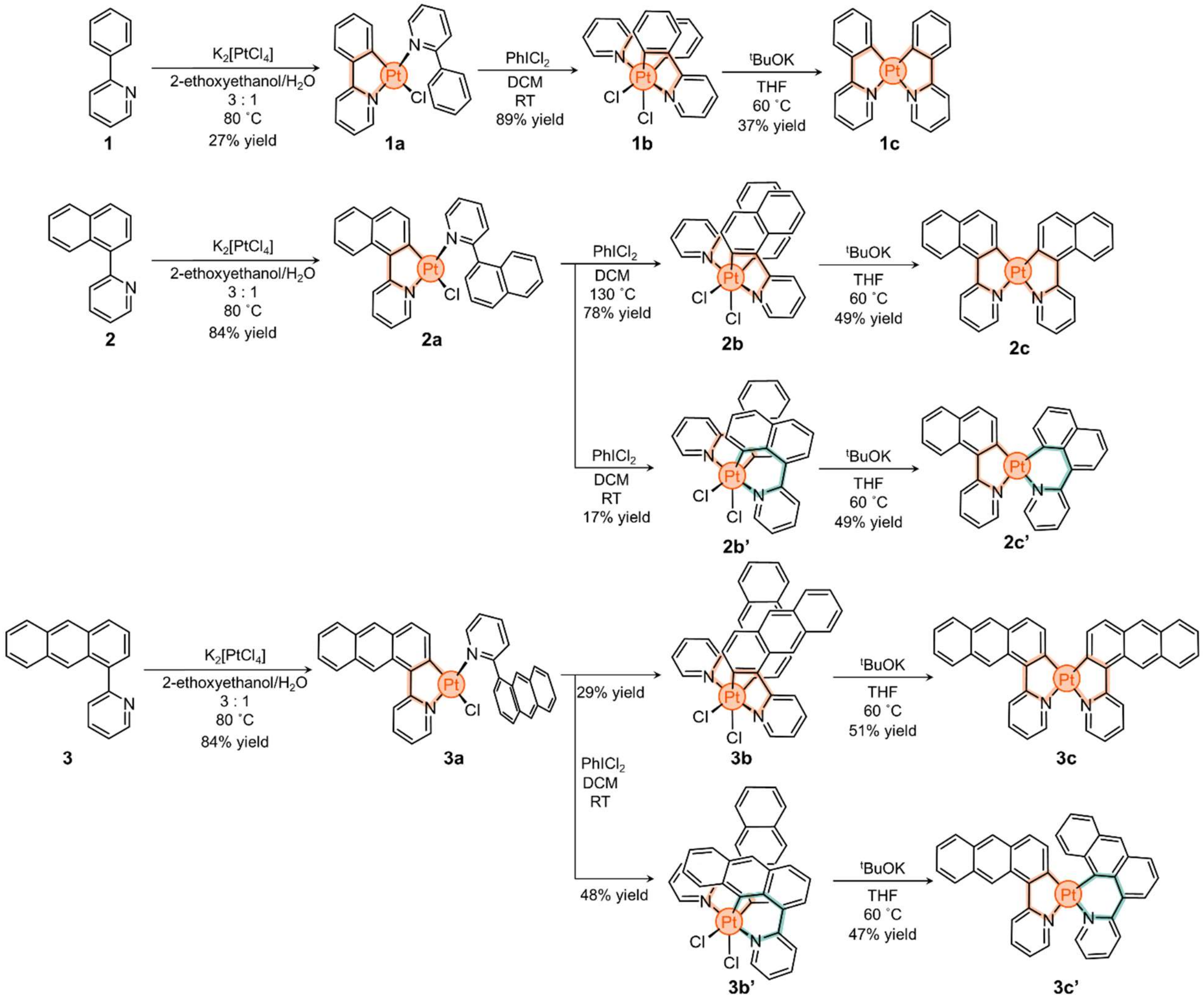

Scheme S2. Synthesis of Pt^{II} and Pt^{IV} complexes.

Ligand 2

Prepared according to a reported procedure[17] using naphthalen-1-ylboronic acid (149 mg, 0.87 mmol), 2-bromopyridine (77 μL, 8.06 mmol), and $Pd(PPh_3)_4$ (37 mg, 0.03 mmol, 4 mol%) at 110 ˚C. The compound was isolated in 91% yield (163 mg, 0.79 mmol) by flash column chromatography (SiO_2) using EtOAc/hexane (1/5, v/v) as eluent (R_f = 0.33). The ^{1}H NMR spectrum of the compound was in agreement with literature values. ^{1}H NMR (400 MHz, chloroform-*d*) δ (ppm): 8.08 (*dd*, *J* = 8.0, 1.6 Hz, 1H), 7.92 (*dd*, *J* = 8.8, 1.3 Hz, 2H), 7.85 (*td*, *J* = 7.8, 1.8 Hz, 1H), 7.63-7.45 (*m*, 5H), 7.35 (*ddd*, *J* = 7.6, 4.9, 1.2 Hz, 1H).

Precursor 1-bromoanthracene

This procedure was adapted from a reported methodology.[18] Precursor 1-aminoanthracene-9,10-dione (3.1 g, 13.7 mmol) was added in 20 portions into a solution of copper(II) bromide (3.6 g, 16.1 mmol) and *t*-butylnitrite (2.8 mL, 23.6 mmol, 1.5 equiv.) prepared in MeCN (330 mL). The reaction mixture was heated at 65 °C for 1 h, cooled to room temperature after this period, and treated with 1 M HCl (300 mL). The generated solid was filtered and washed with H_2O (250 mL) and MeOH (250 mL). The collected solid was dried under reduced pressure to give 3.3 g of crude product. The entire sample was dissolved in *i*-PrOH (40 mL) and cooled with an ice-water bath before $NaBH_4$ was added (1.1 g, 26.7 mmol, 2.3 equiv.) in one portion. After 3 h of stirring in an ice-water bath, the reaction mixture was quenched with water (15 mL) and stirred for 3 h, followed by concentration under vacuum to ca. 30 mL. The residue was extracted with DCM (50 mL × 3), dried over Na_2SO_4, and concentrated under vacuum. The solid residue was dissolved in glacial acetic acid (115 mL) and $SnCl_2{\cdot}2H_2O$ (6.4 g, 22.1 mmol, 2.5 equiv.) was then added into the solution in one portion. The reaction mixture was heated at 125 °C for 2 h, cooled to room temperature, diluted with water (100 mL), and extracted with toluene (50 mL × 3). The compound was isolated by column chromatography (SiO_2, R_f = 0.56) using hexane as the eluent to give 469 mg (1.82 mmol, 17% yield) of the target compound as a yellowish solid. ^{1}H NMR data were in

agreement with literature reports. ^{1}H NMR (400 MHz, chloroform-*d*) δ (ppm): 8.82 (*s*, 1H), 8.44 (s, 1H), 8.13-8.00 (*m*, 2H), 7.98 (*d*, *J* = 9.0 Hz, 1H), 7.79 (*d*, *J* = 7.1 Hz, 1H), 7.55-7.50 (*m*, 2H), 7.36 (*t*, *J* = 7.6 Hz, 1H).

Precursor 2-(anthracen-1-yl)-4,4,5,5-tetramethyl-1,3,2-dioxaborolane

This procedure was adapted from a previous report.[19] 1-Bromoanthracene (150 mg, 0.58 mmol), KOAc (190 mg, 1.94 mmol), bis(pinacolato)diboron (192 mg, 0.76 mmol), Pd(dppf)Cl_2·DCM (24 mg, 0.03 mmol), and anhydrous, degassed DMF (5 mL) were added into a Schlenk flask. The reaction mixture was heated to 90 °C for 18 h under nitrogen. After cooling the system to room temperature, the reaction was quenched with brine (ca. 30 mL). The organic materials were extracted with toluene (3 × 10 mL) and the organic layer was washed with brine (3 × 30 mL), dried over anhydrous Na_2SO_4, and evaporated. Column chromatography (SiO_2, DCM, R_f = 0.43) afforded the desired compound as pale-yellow powder (138 mg, 0.45 mmol, 78% yield). ^{1}H NMR was in agreement with literature values. ^{1}H NMR (400 MHz, chloroform-*d*) δ (ppm): 9.34 (*s*, 1H), 8.41 (*s*, 1H), 8.13-8.05 (*m*, 3H), 8.02-7.95 (*m*, 1H), 7.79 (*d*, *J* = 7.1 Hz, 1H), 7.50-7.42 (*m*, 3H).

Ligand 3

2-(Anthracen-1-yl)-4,4,5,5-tetramethyl-1,3,2-dioxaborolane (129 mg, 0.43 mmol), 2-bromopyridine (53 μL, 0.55 mmol), K_2CO_3 (88 mg, 0.64 mmol), Pd(PPh_3)$_4$ (25 mg, 0.02 mmol), degassed toluene (6 mL), degassed EtOH (2 mL), and degassed H_2O (2 mL) were added into a Schlenk flask. The mixture was heated to 100 °C for 21 h under N_2. After cooling the system to room temperature, the reaction was quenched with brine (ca. 10 mL) and the organic phase was extracted with toluene (3 × 10 mL), dried over anhydrous Na_2SO_4, and evaporated. The crude product was purified by column chromatography (SiO_2, R_f = 0.25) using AcOEt/DCM (1/9, v/v) as the eluent to afford **3** as a yellow solid (86 mg, 0.34 mmol, 80% yield). ^{1}H NMR (400 MHz, chloroform-*d*) δ (ppm): 8.87 (*d*, *J* = 4.4 Hz, 1H), 8.67 (*s*, 1H), 8.50 (*s*, 1H), 8.07 (*d*, *J* = 8.3 Hz, 1H),

8.01 (*d*, *J* = 8.3 Hz, 1H), 7.92 (*d*, *J* = 8.3 Hz, 1H), 7.87 (*td*, *J* = 7.8, 1.6 Hz, 1H), 7.67 (*d*, *J* = 7.8 Hz, 1H), 7.62-7.36 (m, 5H); ^{13}C {^{1}H} NMR (101 MHz, chloroform-*d*) δ (ppm): 159.9, 149.8, 138.6, 136.7, 132.25, 132.22, 131.6, 129.7, 129.4, 128.9, 128.0, 127.2, 126.9, 125.8, 125.5, 125.3, 124.9, 124.8, 122.3. HRMS: [**3** + H]$^+$, *m*/*z* = 256.1123 Da (exp), 256.1126 Da (calc).

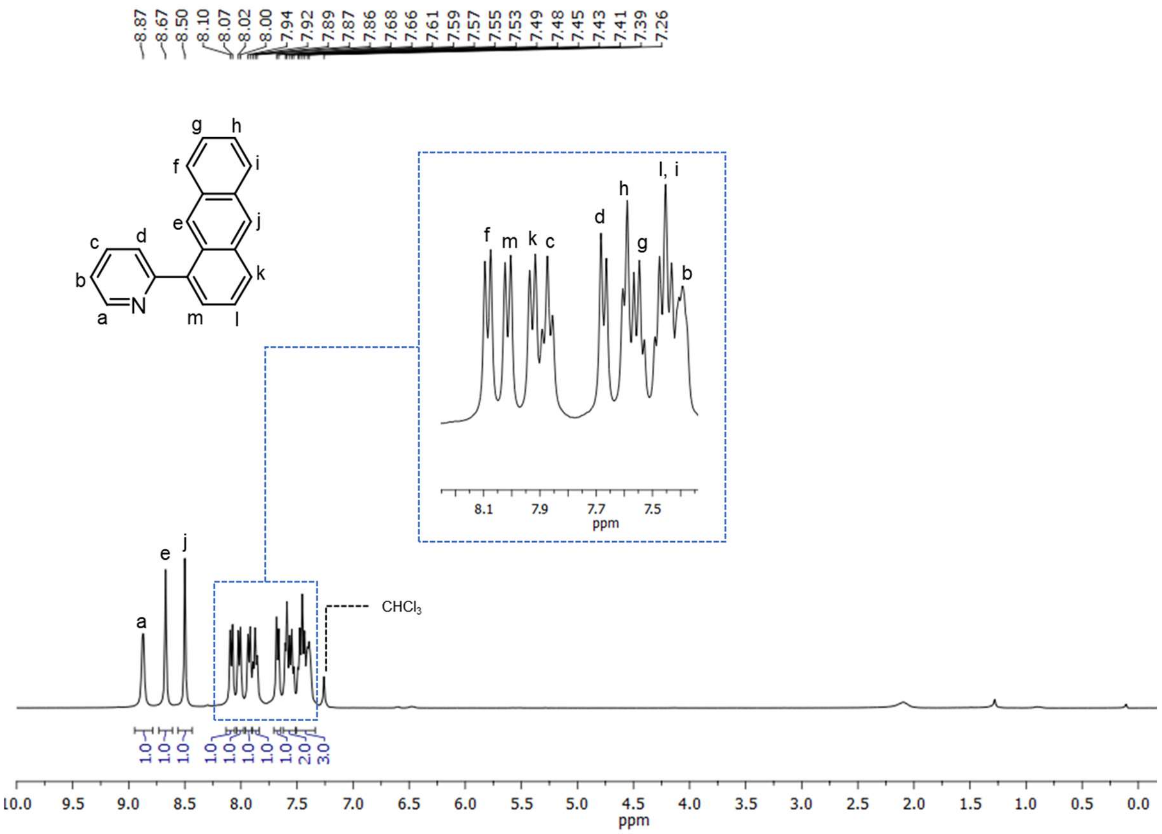

Figure S18. ^{1}H NMR spectrum (400 MHz, chloroform-*d*) of **3**.

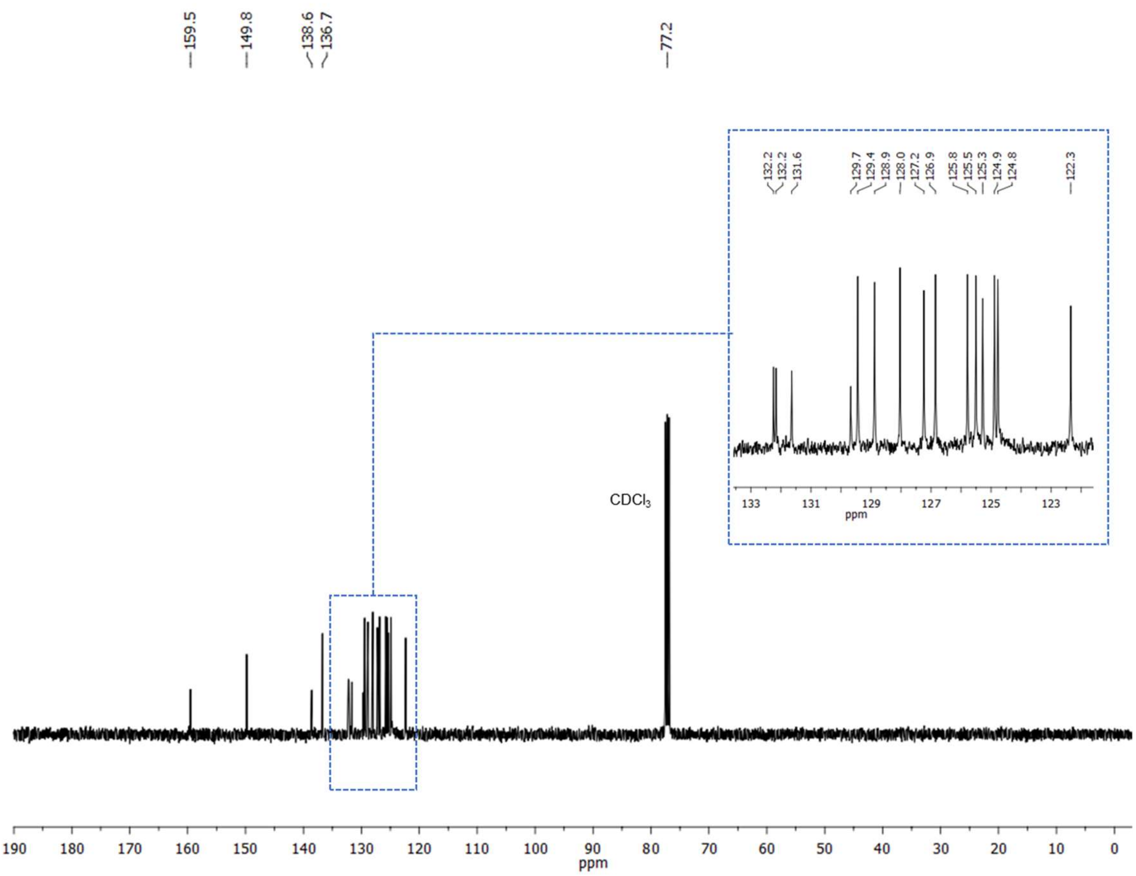

Figure S19. $^{13}C\{^1H\}$ NMR spectrum (101 MHz, chloroform-*d*) of **3**.

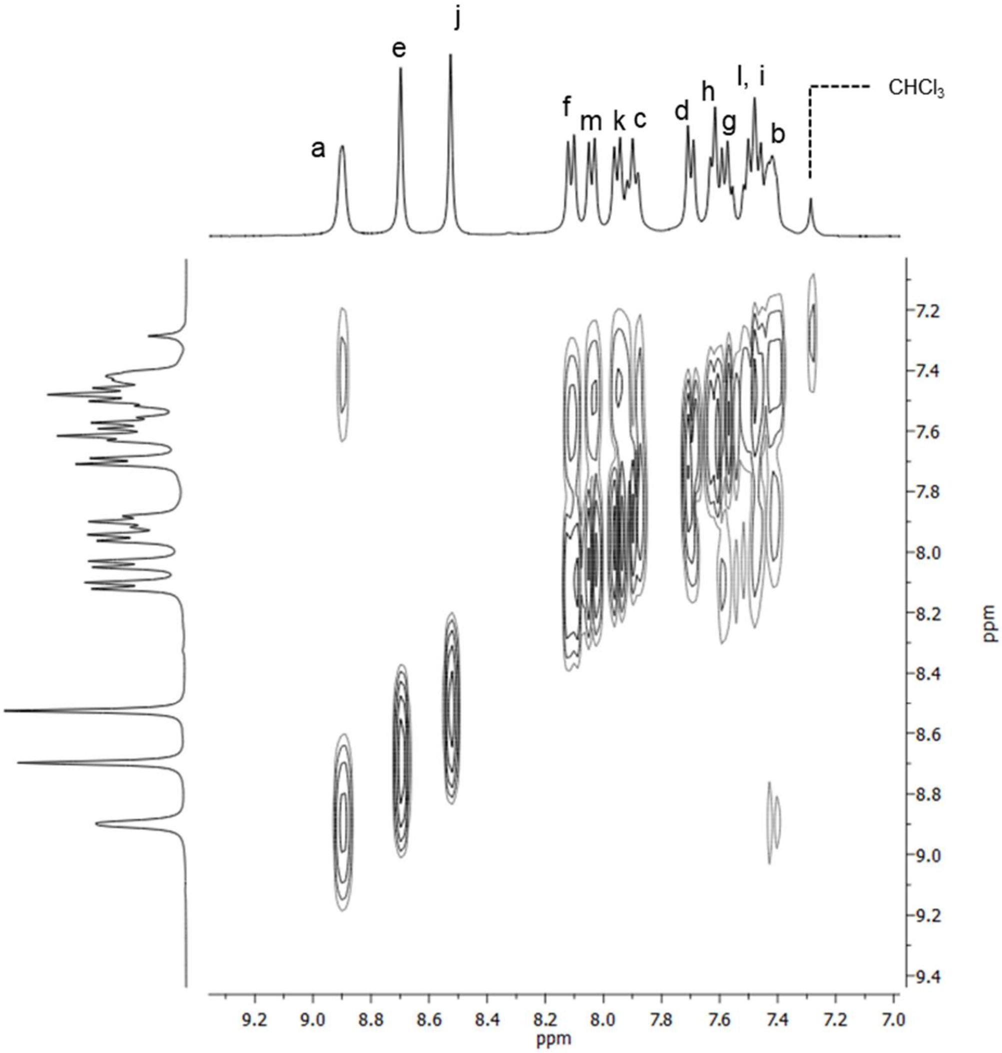

Figure S20. ^{1}H-^{1}H COSY NMR spectrum (400 MHz, chloroform-*d*) of **3**.

Complex 2a

Ligand **2** (54 mg, 0.11 mmol) and $K_2[PtCl_4]$ (40 mg, 0.27 mmol) were suspended in a 3/1 (v/v) mixture of 2-ethoxyethanol (1.5 mL) and H_2O (0.5 mL) and heated at 80 °C for 22 h under a N_2 atmosphere. The reaction was allowed to cool down to room temperature and concentrated under reduced pressure. The resulting orange-brown precipitate was collected by vacuum filtration and washed with H_2O (30 mL) and hexanes (30 mL). Further purification by column chromatography (SiO_2, DCM, R_f = 0.54) gave compound **2a** as a yellow powder (59 mg, 0.09 mmol, 84% yield). ^{1}H NMR (400 MHz, DCM-d_2) δ (ppm): 9.75 (*dd*, *J* = 5.8, 1.1 Hz, 9.42 (*dd*, *J* = 5.8, 1.1 Hz), *9.32* (*dd*, *J* = 5.8, 0.7 Hz), 9.26 (*dd*, *J* = 5.8, 0.7 Hz), 8.72 (*dd*, *J* = 7.2, 0.8), 8.42 (*d*, *J* = 8.6 Hz), 8.21 (*d*, *J* = 8.6 Hz), 8.15 (*d*, *J* = 8.3 Hz), 8.08 – 7.98 (*m*), 7.92 – 7.70 (*m*), 7.53 (*d*, *J* = 7.9 Hz), 7.51 – 7.09 (*m*), 7.04 (*d*, *J* = 8.4 Hz), 6.99 (*td*, *J* = 6.6, 1.2 Hz), 6.87 (*td*, *J* = 6.6, 1.2 Hz), 6.80 (*d*, *J* = 8.4 Hz), 6.35 (*d*, *J* = 8.4 Hz) ; ^{13}C {^{1}H} NMR (101 MHz, DCM-d_2) δ (ppm): 167.89, 167.87, 161.4, 160.2, 154.7, 154.2, 151.8, 151.6, 146.2, 144.0, 138.36, 138.32, 137.8, 137.4, 137.2, 137.0, 136.9, 136.5, 133.4, 133.3, 131.8, 131.5, 131.3, 131.2, 130.5, 130.2, 130.07, 130.03, 130.01, 129.8, 129.66, 129.63, 129.6, 129.5, 129.39, 129.33, 129.0, 128.4, 128.0, 127.8, 127.2, 126.9, 126.7, 126.3, 126.2, 125.7, 124.8, 124.6, 124.37, 124.31, 123.5, 123.1, 122.07, 122.05, 121.5, 121.2, 120.75, 120.72. HRMS: [**2a** - Cl]$^+$, *m*/*z* = 604.1354 Da (exp), 604.1353 Da (calc).

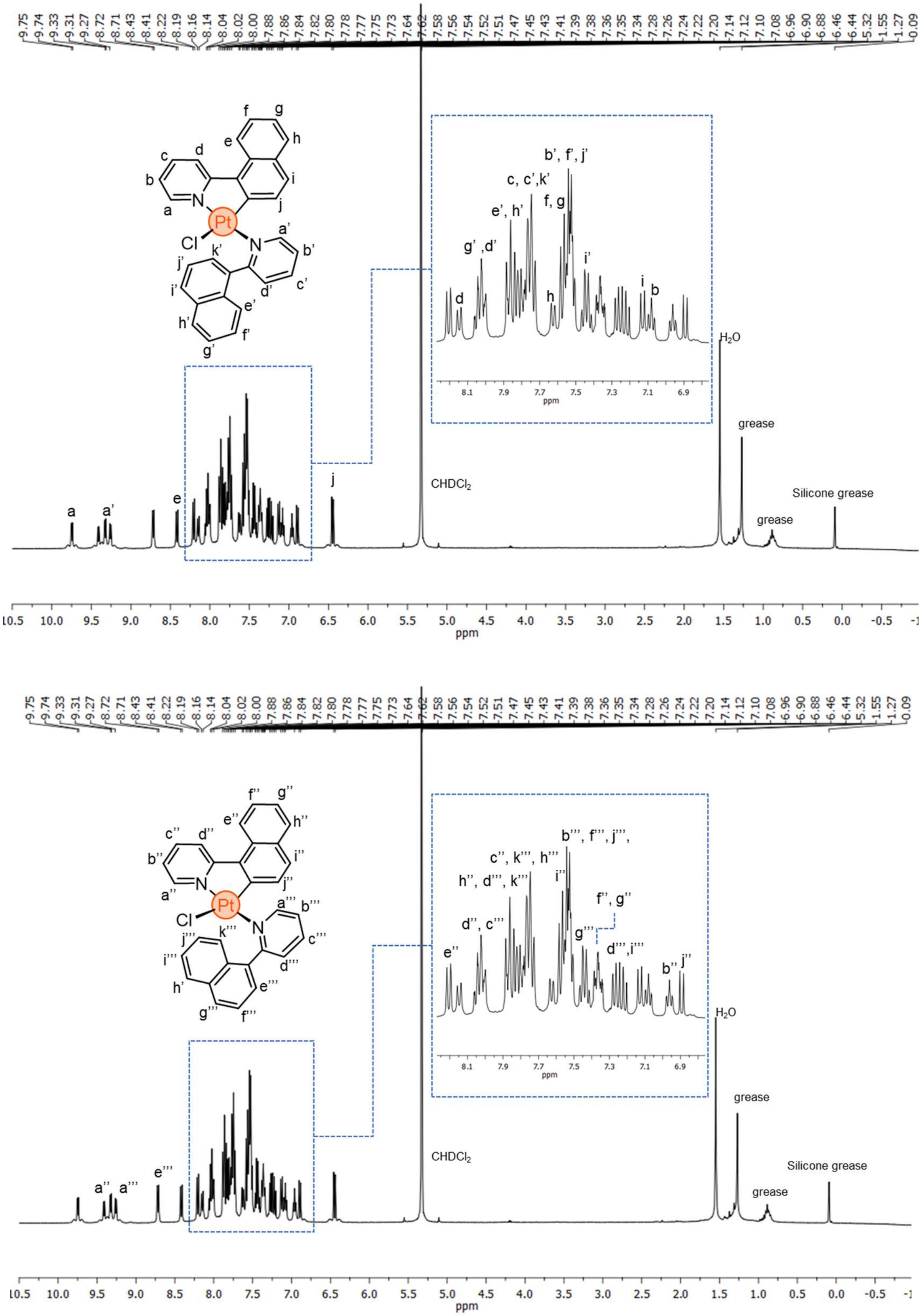

Figure S21. ^{1}H NMR spectrum (400 MHz, DCM-d_2) of **2a** (signals have been assigned to each rotamer).

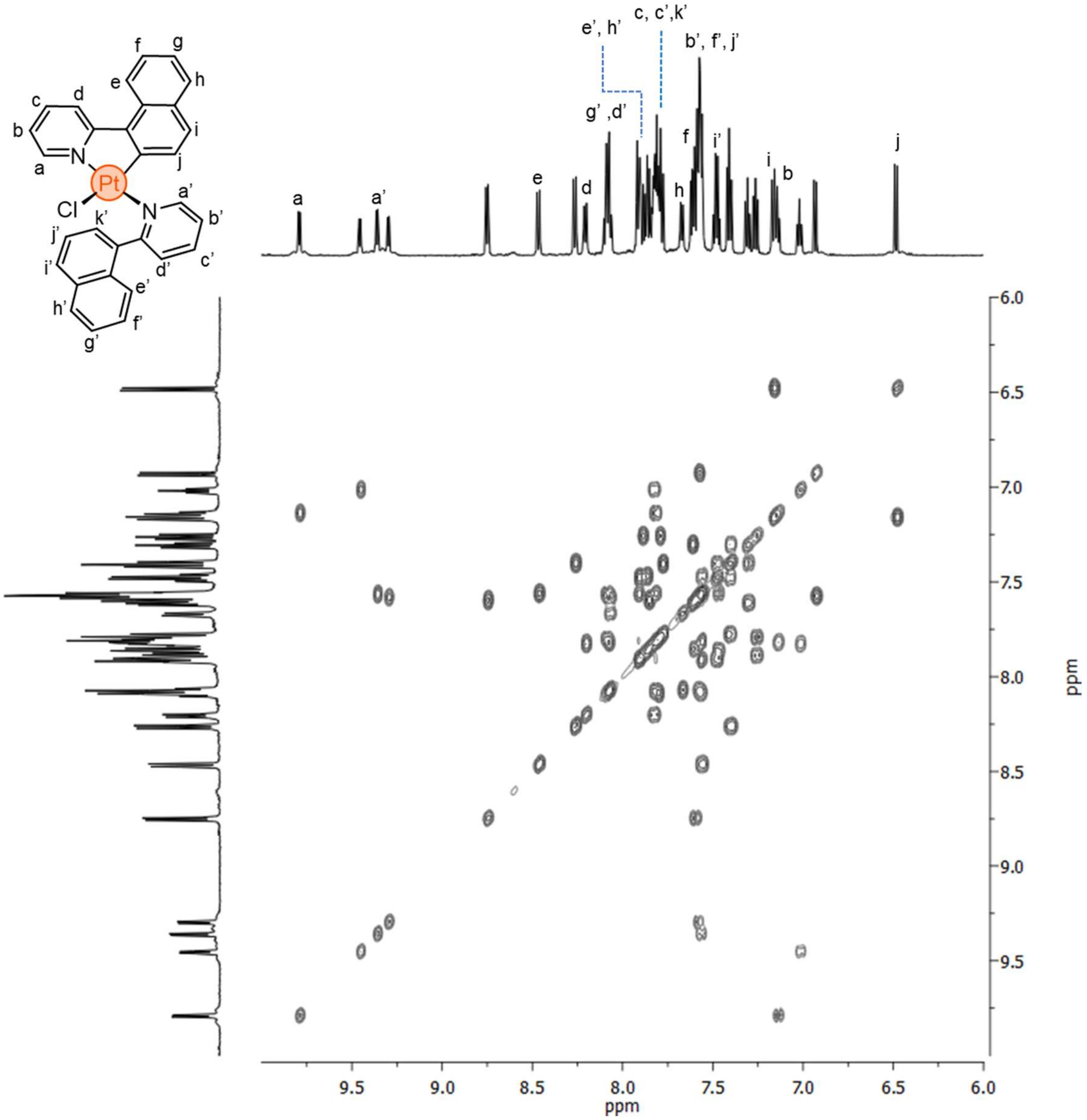

Figure S22. ^{1}H–^{1}H COSY NMR spectrum (400 MHz, DCM-d_2) of **2a** (signals have been assigned to each rotamer).

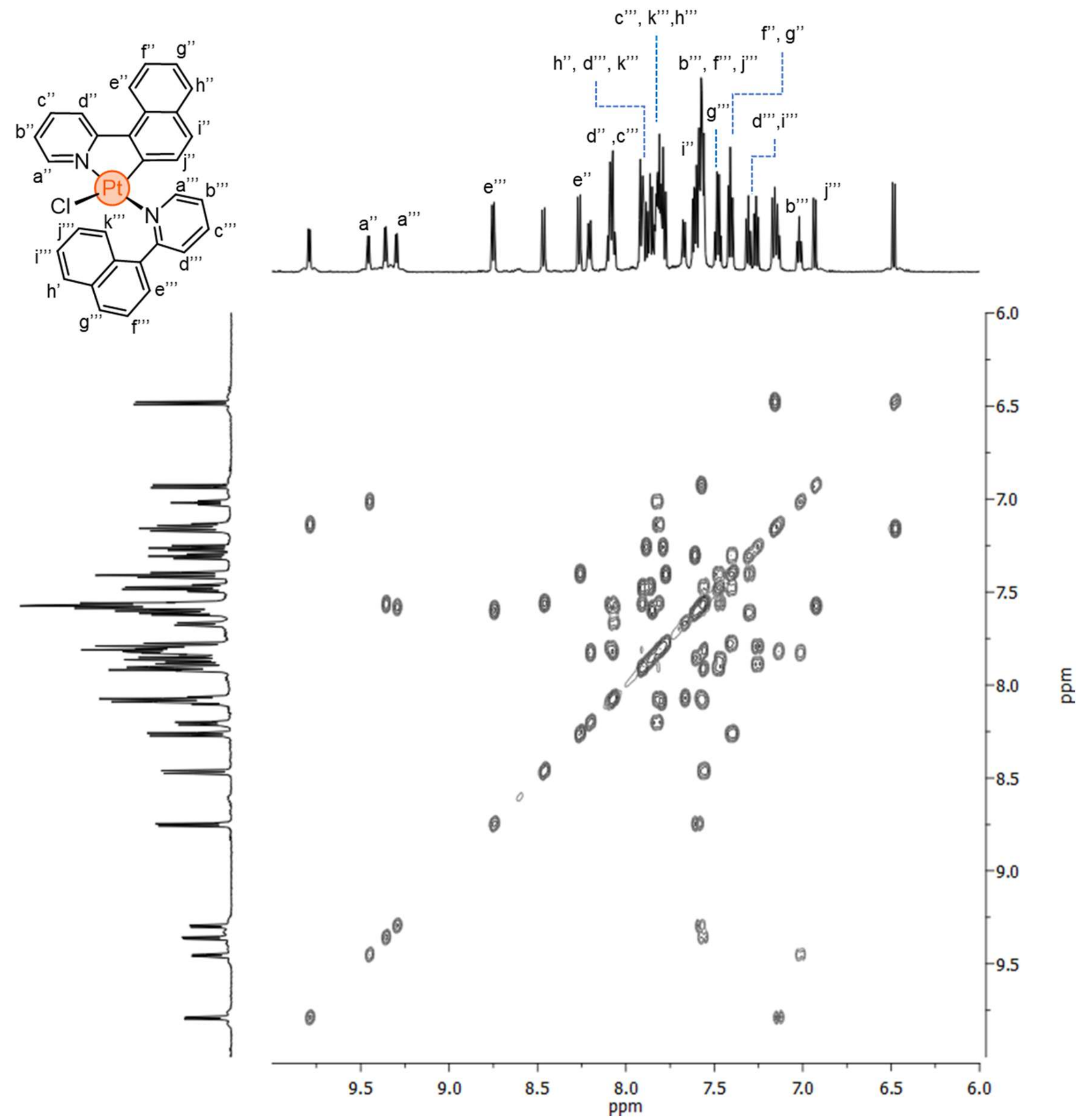

Figure S22 (continued). ^{1}H–^{1}H COSY NMR spectrum (400 MHz, DCM-d_2) of **2a** (signals have been assigned to each rotamer).

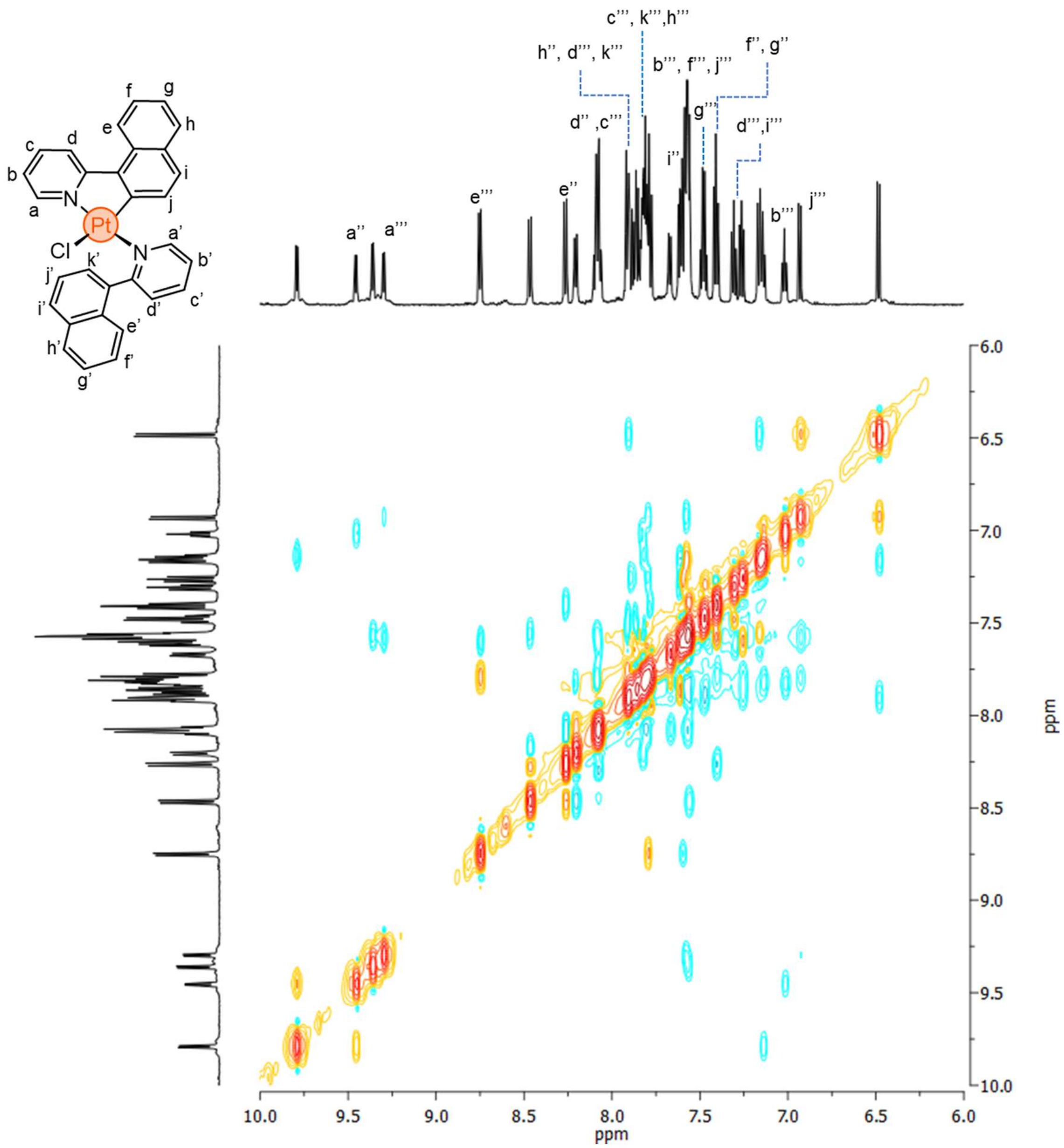

Figure S23. ^{1}H–^{1}H NOESY (blue) and EXSY (orange) NMR spectrum (400 MHz, DCM-d_2) of **2a** (signals have been assigned to each rotamer).

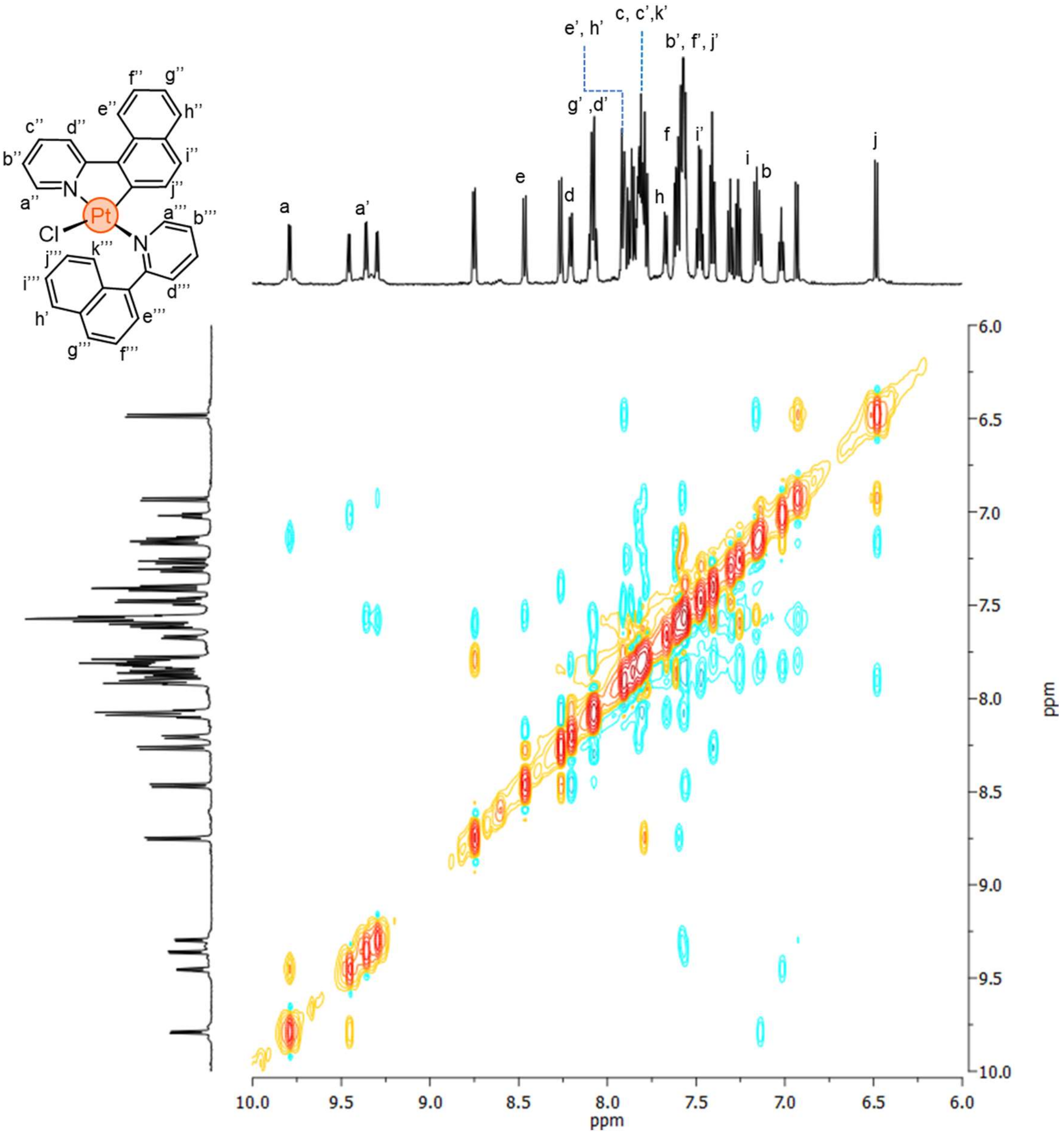

Figure S23 (continued). ^{1}H–^{1}H NOESY (blue) and EXSY (orange) NMR spectrum (400 MHz, DCM-d_2) of **2a** (signals have been assigned to each rotamer).

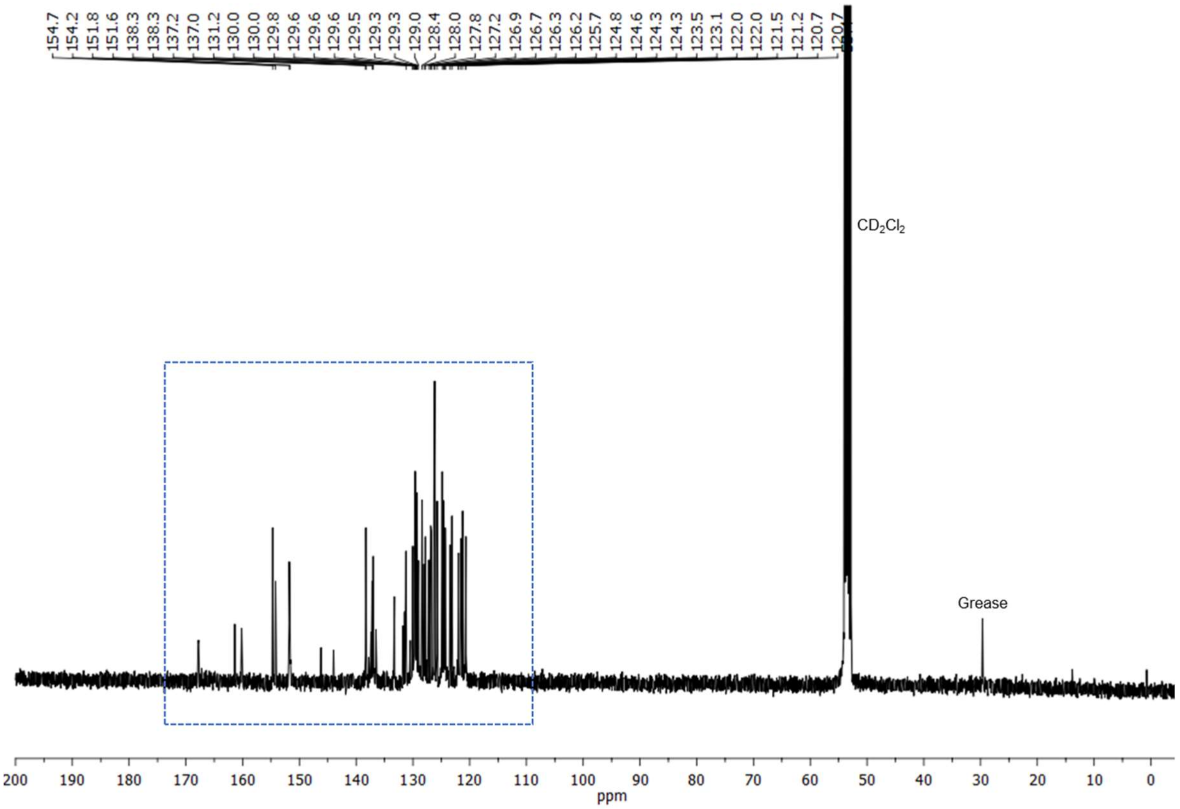

Figure S24. $^{13}C\{^1H\}$ NMR spectrum (101 MHz, DCM-d_2) of **2a**.

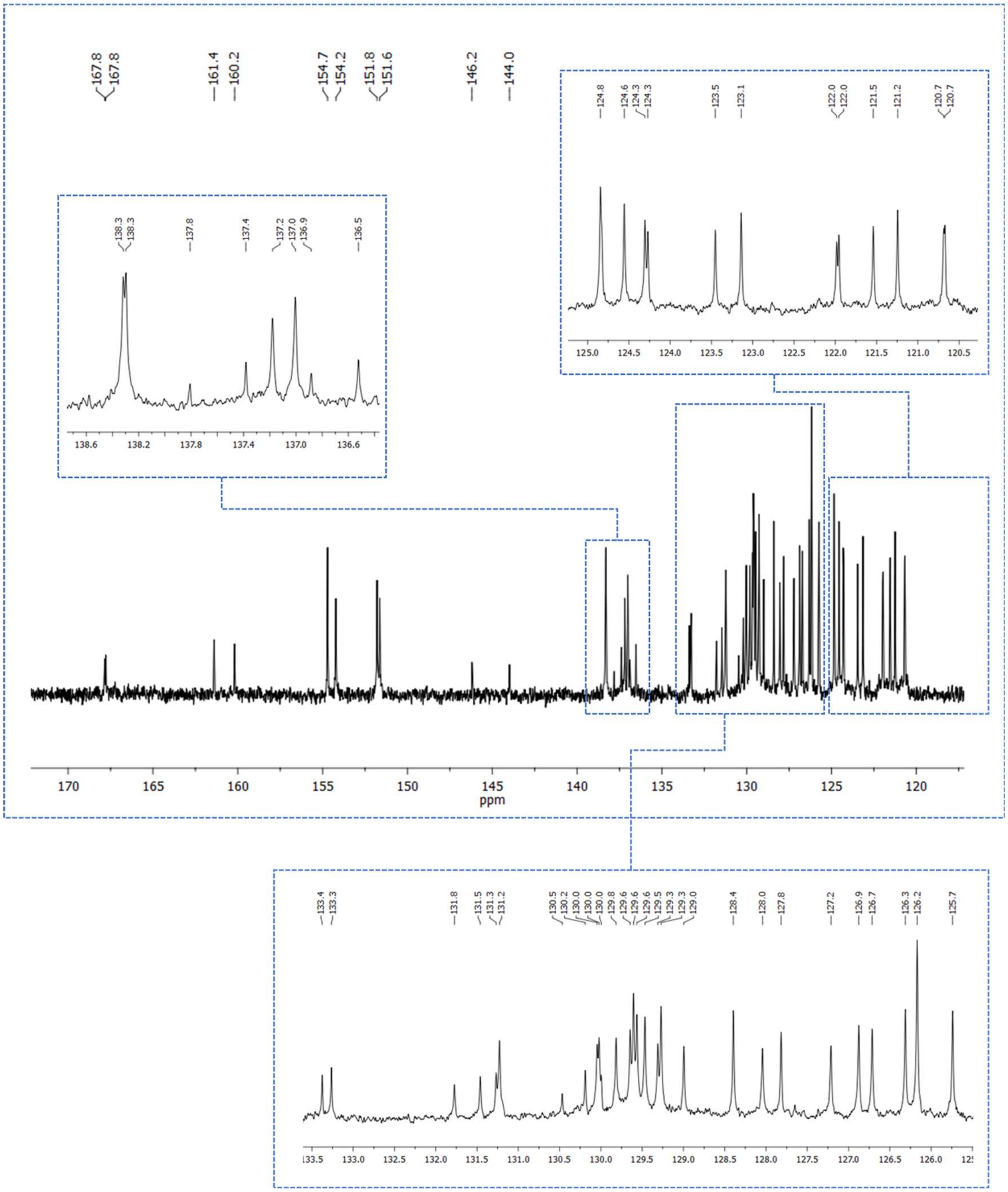

Figure S25. Zoomed-in $^{13}C\{^{1}H\}$ NMR spectrum (101 MHz, DCM-d_2) of **2a**.

Compound 3a

Ligand **3** (47 mg, 0.18 mmol) and $K_2[PtCl_4]$ (31 mg, 0.07 mmol) were suspended in a 3/1 (v/v) mixture of 2-ethoxyethanol (1.5 mL) and H_2O (0.5 mL), and heated at 80 °C for 16 h under a N_2 atmosphere. The reaction was allowed to cool down to room temperature and concentrated under reduced pressure. The resulting orange-brown precipitate was collected by vacuum filtration and washed with H_2O (10 mL × 3) and hexanes (10 mL × 3). Purification by column chromatography (SiO_2, DCM, R_f = 0.53) yielded **3a** as a red-brown powder (44 mg, 0.06 mmol, 80% yield). ^{1}H NMR (400 MHz, DCM-d_2) δ (ppm): 9.66 (*dd*, *J* = 5.9, 1.3 Hz, 9.32 – 9.23 (*m*), 8.86 (*s*), 8.62 (*s*), 8.56 (*dd*, *J* = 7.0, 1.0 Hz), 8.36 (*s*), 8.33 (*s*), 8.32 (*d*, *J* = 4.4 Hz), 8.25 (*d*, *J* = 8.5 Hz), 8.21 (*s*), 8.11 (*d*, *J* = 8.3 Hz), 8.08-7.99 (*m*), 7.98 – 7.83 (*m*), 7.82 – 7.59 (*m*), 7.56 – 7.43 (*m*), 7.43 – 7.24 (*m*), 7.15 – 7.06 (*m*), 7.03 (*t*, *J* = 6.7 Hz), 6.86 (*t*, *J* = 6.7 Hz), 6.81 (*d*, *J* = 8.6 Hz), 6.53 (*d*, *J* = 8.7 Hz) ; ^{13}C {^{1}H} NMR (100101 MHz, CD_2Cl_2) δ 168.43, 168.41, 168.35, 168.32, 162.0, 160.9, 155.1, 154.7, 152.2, 152.08, 152.03, 148.5, 146.6, 138.8, 138.0, 137.9, 137.7, 136.8, 136.0, 134.9, 134.8, 133.0, 132.6, 132.1, 132.03, 132.01, 131.9, 131.8, 131.7, 131.5, 131.1, 130.9, 130.8, 130.69, 130.67, 130.63, 130.5, 130.4, 130.35, 130.33, 130.1, 129.9, 129.8, 129.5, 129.2, 129.0, 128.6, 128.53, 128.50, 128.4, 128.3, 128.28, 128.25, 128.1, 128.0, 127.9, 127.8, 127.3, 126.7, 126.6, 126.5, 126.4, 126.23, 126.21, 126.0, 125.4, 125.3, 125.1, 124.9, 124.5, 124.22, 124.20, 122.0, 120.7, 119.8, 119.3. HRMS: [**3a** - Cl]$^+$, *m*/*z* = 705.1674 Da (exp), 705.1744 Da (calc).

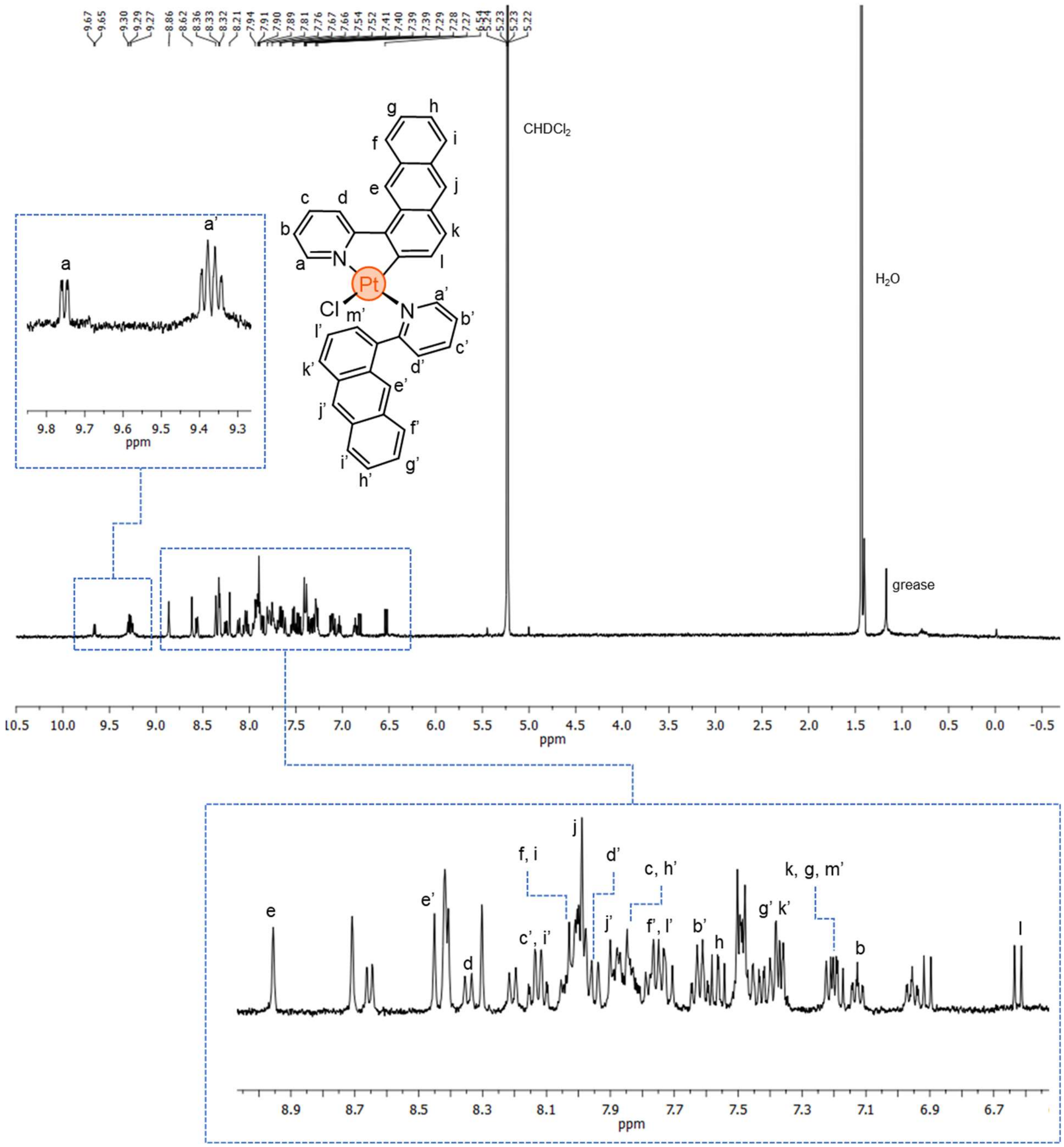

Figure S26. ^{1}H NMR spectrum (400 MHz, DCM-d_2) of **3a** (signals have been assigned to each rotamer).

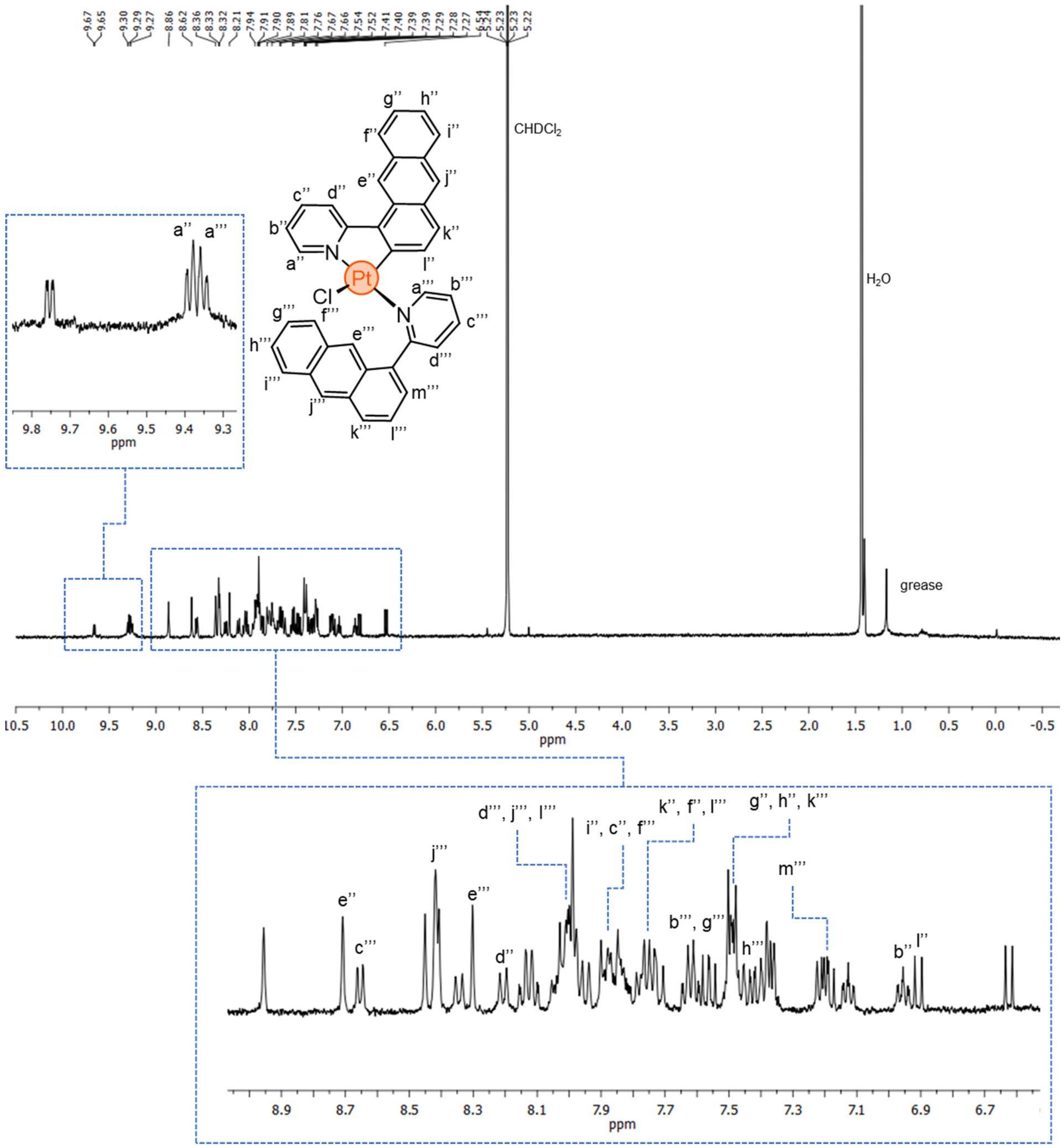

Figure S26 (continued). ^{1}H NMR spectrum (400 MHz, DCM-d_2) of **3a** (signals have been assigned to each rotamer).

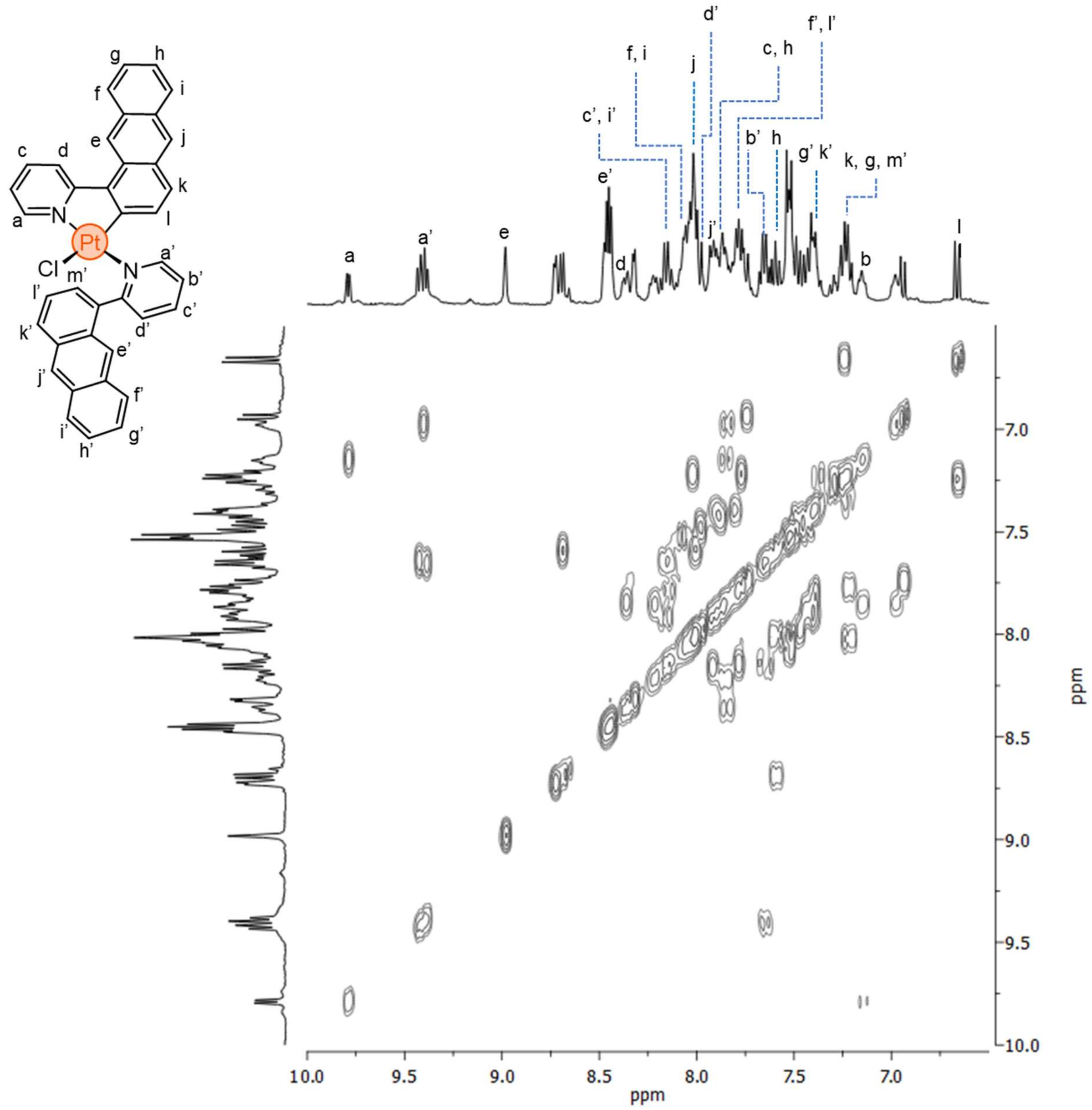

Figure S27. ^1H–^1H COSY NMR spectrum (400 MHz, DCM-d_2) of **3a** (signals have been assigned to each rotamer).

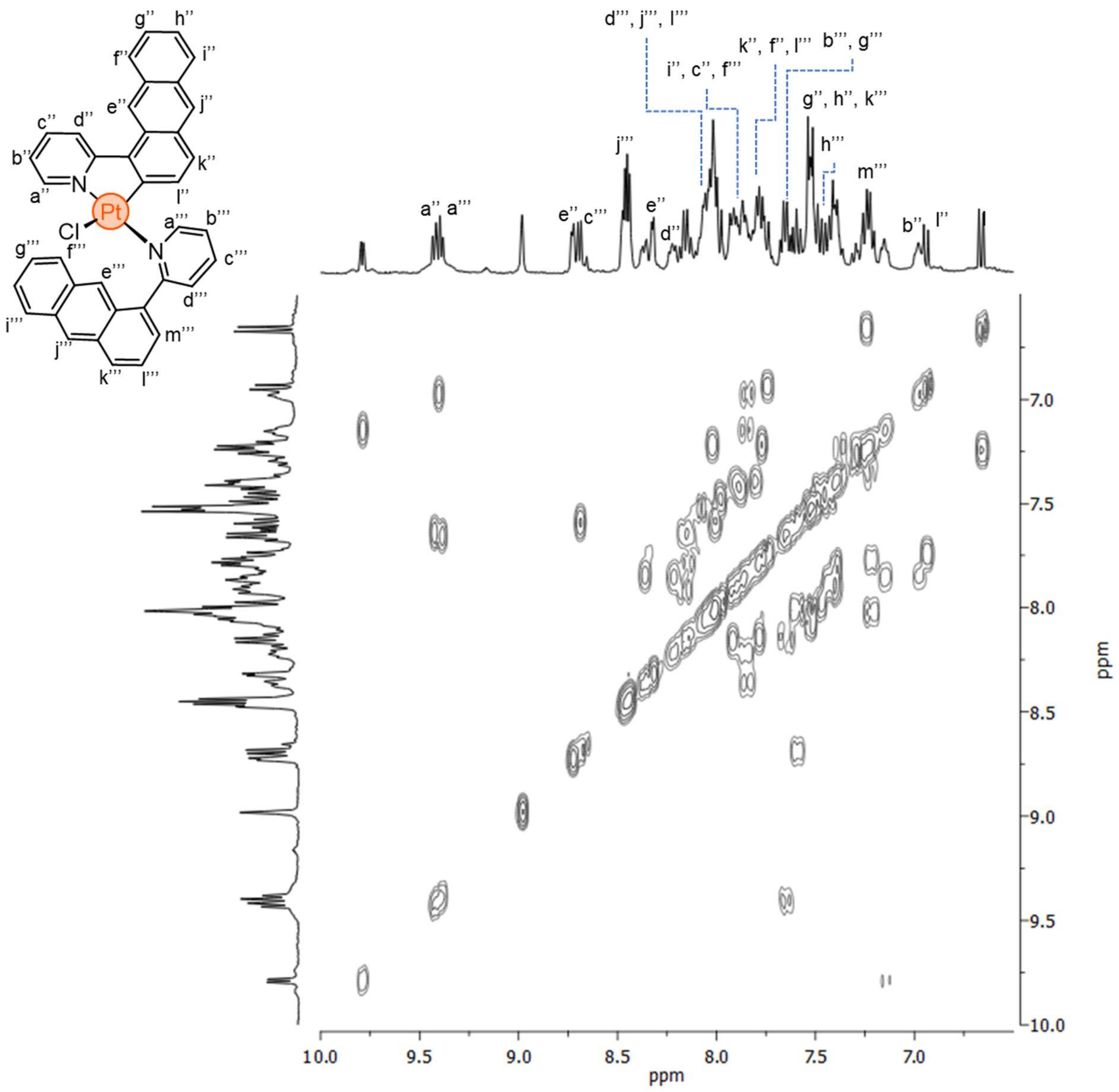

Figure S27 (continued). ^{1}H–^{1}H COSY NMR spectrum (400 MHz, DCM-d_2) of **3a** (signals have been assigned to each rotamer).

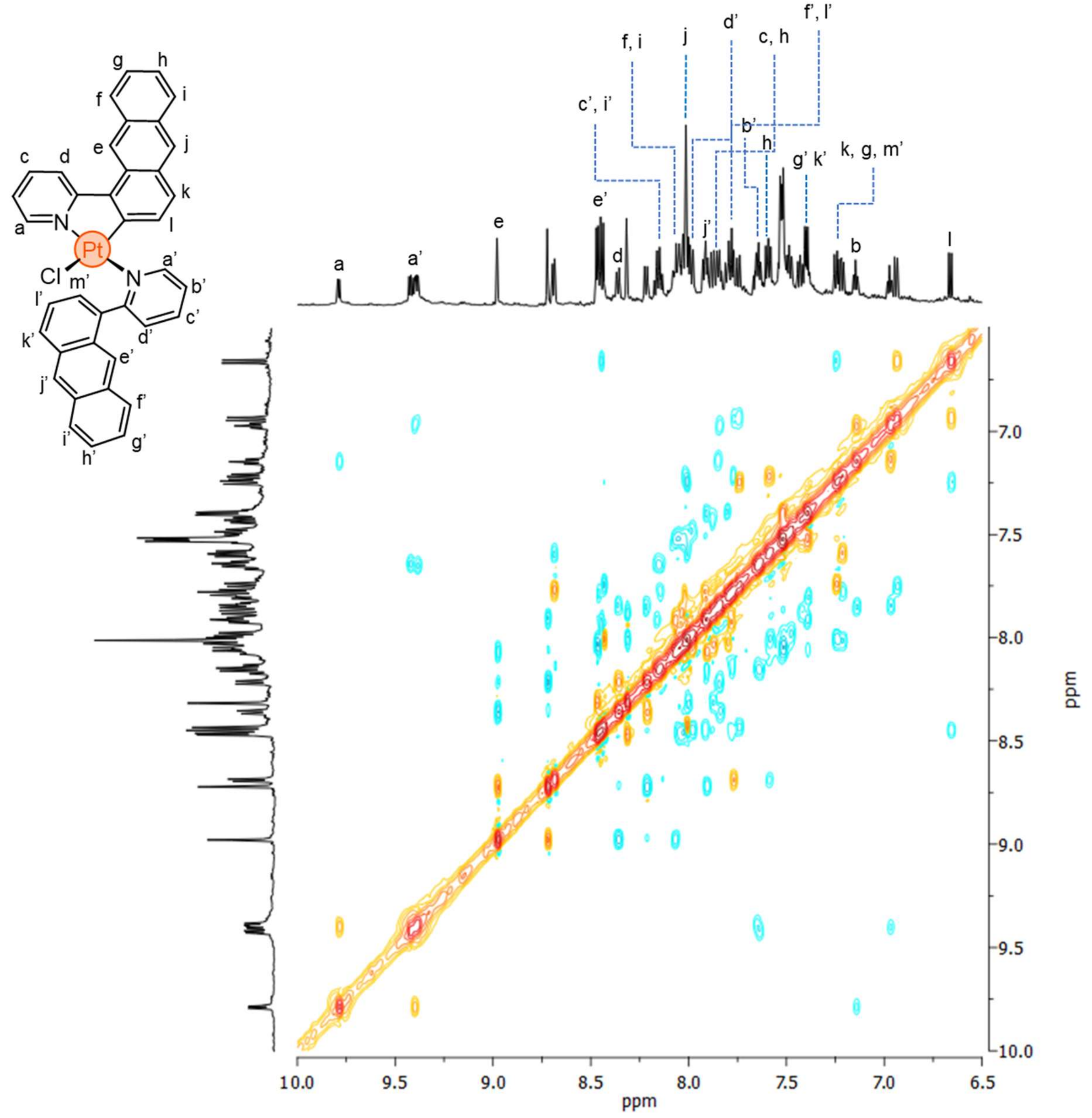

Figure S28. ^{1}H-^{1}H NOESY (blue) and EXSY (orange) NMR spectra (400 MHz, DCM-d_2) of **3a** (signals have been assigned to each rotamer).

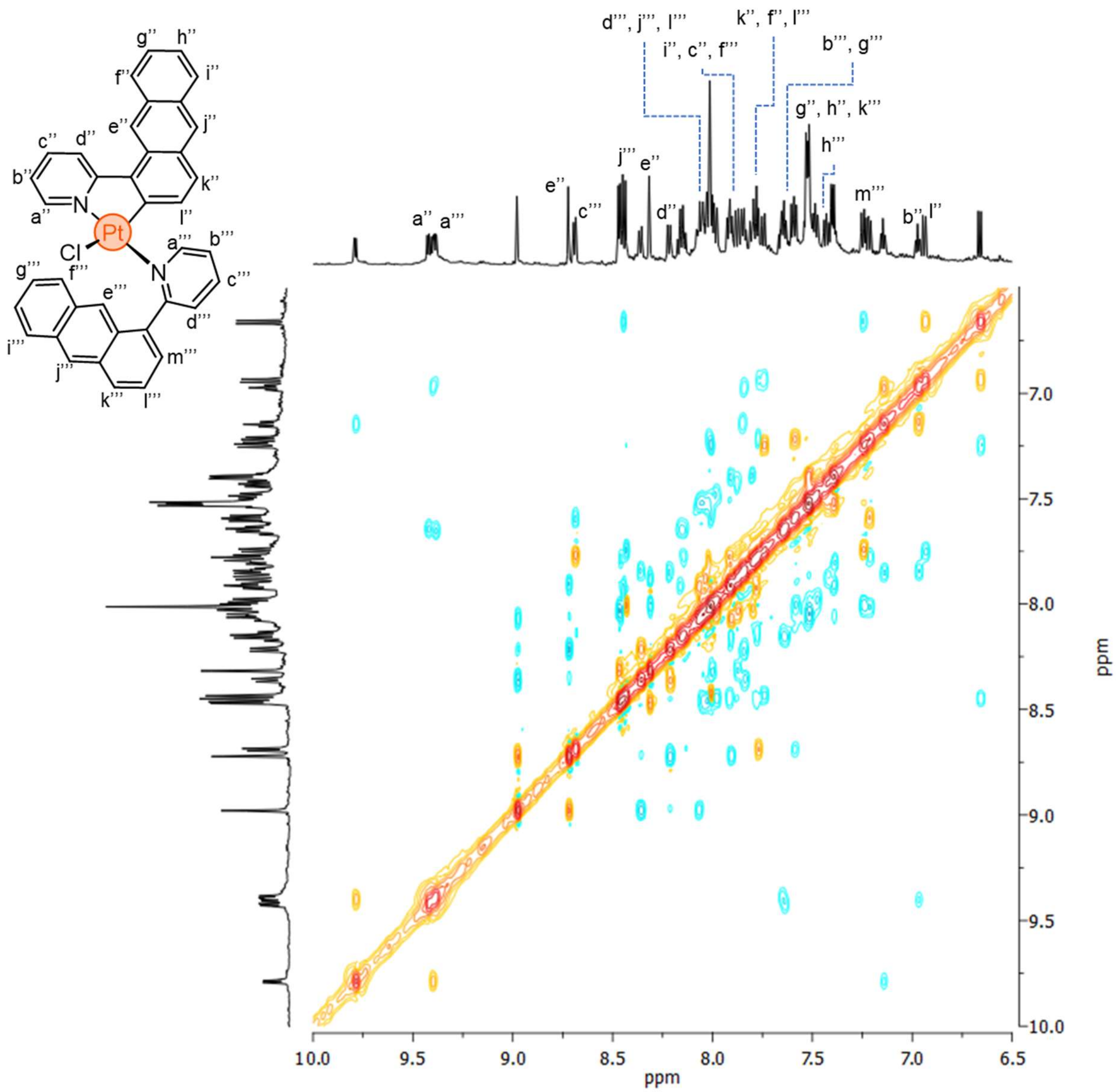

Figure S28 (continued). ^{1}H-^{1}H NOESY (blue) and EXSY (orange) NMR spectra (400 MHz, DCM-d_2) of **3a** (signals have been assigned to each rotamer).

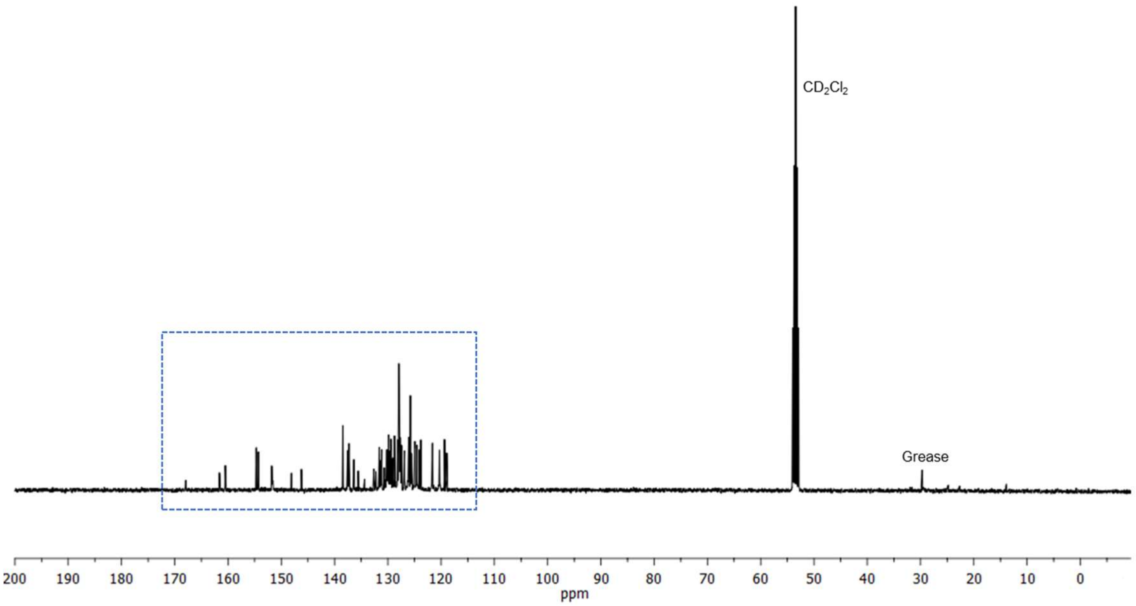

Figure S29. $^{13}C\{^{1}H\}$ NMR spectrum (101 MHz, DCM-d_2) of **3a**.

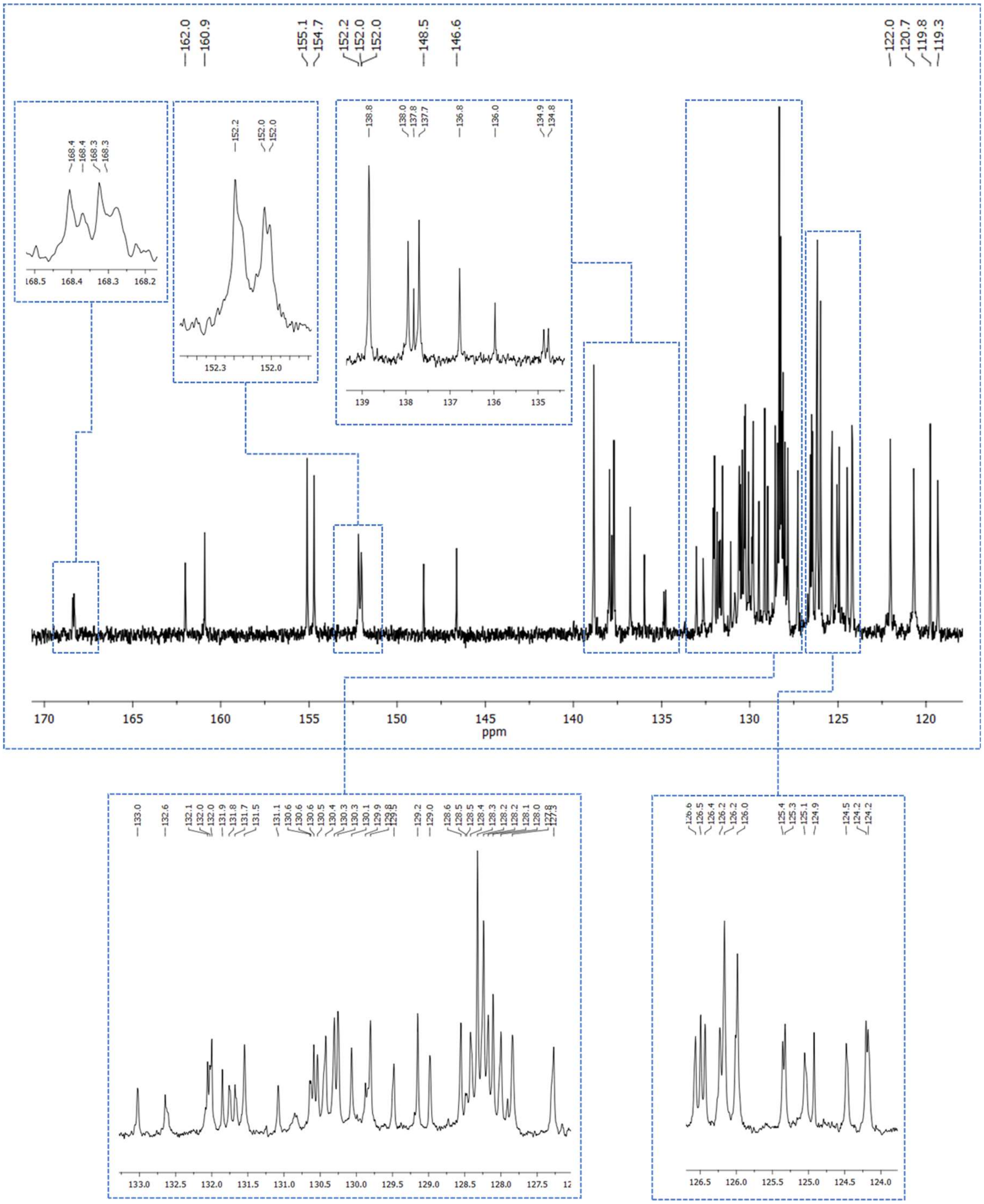

Figure S30. Zoomed-in $^{13}C\{^1H\}$ NMR spectrum (101 MHz, DCM-d_2) of **3a**.

Complex 2b

Compound **2a** (5.0 mg, 7.81 μmol) was dissolved in 1,1,2,2-tetrachloroethane (1.5 mL) and heated to 130 °C. $PhICl_2$ (2.4 mg, 8.77 μmol) was added in one portion and the reaction mixture was stirred for 5 min. The solvent was removed under reduced pressure and the resulting yellow residue was washed with cold (0 °C) DCM (5 mL × 3) to give pure compound **2b** as an off-white solid (4.1 mg, 5.82 μmol, 78% yield). R_f = 0.39 (SiO_2, DCM/hexanes/acetone, 2/2/1, v/v/v). ^1H NMR (400 MHz, DCM-d_2) δ (ppm): 10.17 (*dd*, *J* = 6.0, 1.2 Hz, 2H), 8.66 (*d*, *J* = 8.3 Hz, 2H), 8.58 (*d*, *J* = 8.7 Hz, 2H), 8.25 (*td*, *J* = 8.0, 1.6 Hz, 2H), 7.77 (*d*, *J* = 8.0 Hz, 2H), 7.64 (*td*, *J* = 7.8, 1.3 Hz, 2H), 7.59 (*td*, *J* = 6.7, 1.3 Hz, 2H), 7.45 (*td*, *J* = 7.5, 0.5 Hz, 2H), 7.41 (*d*, *J* = 8.6 Hz, 2H), 6.37 (*d*, *J* = 8.6 Hz, 2H); $^{13}C\{^1H\}$ NMR (101 MHz, DMSO-d_6) δ (ppm): 163.2, 150.1, 144.1, 142.1, 134.0, 132.2, 132.1, 129.8, 129.6, 128.5, 125.5, 125.4, 124.5, 124.3, 122.3. HRMS: [**2b** - Cl]$^+$, *m*/*z* = 638.0947 Da (exp), 638.0963 Da (calc).

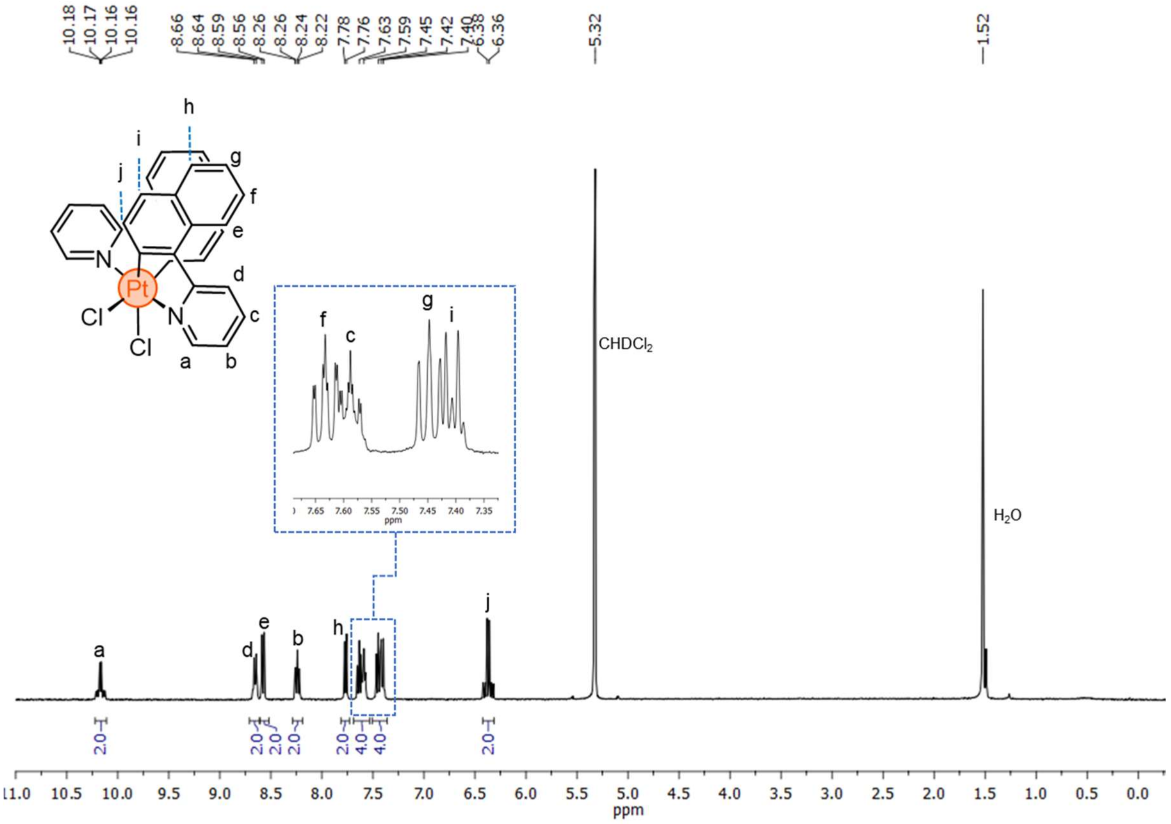

Figure S31. ^1H NMR spectrum (400 MHz, DCM-d_2) of **2b**.

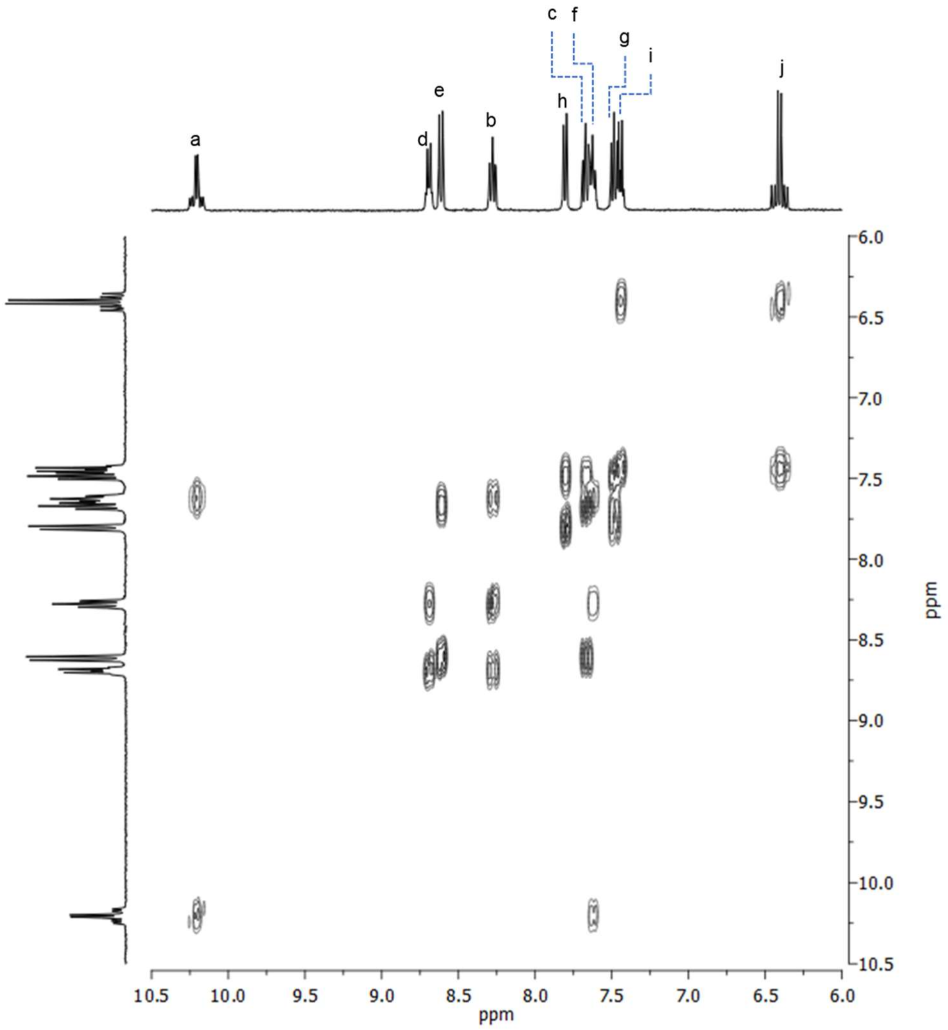

Figure S32. ^1H-^1H COSY NMR spectrum (400 MHz, DCM-d_2) of **2b**.

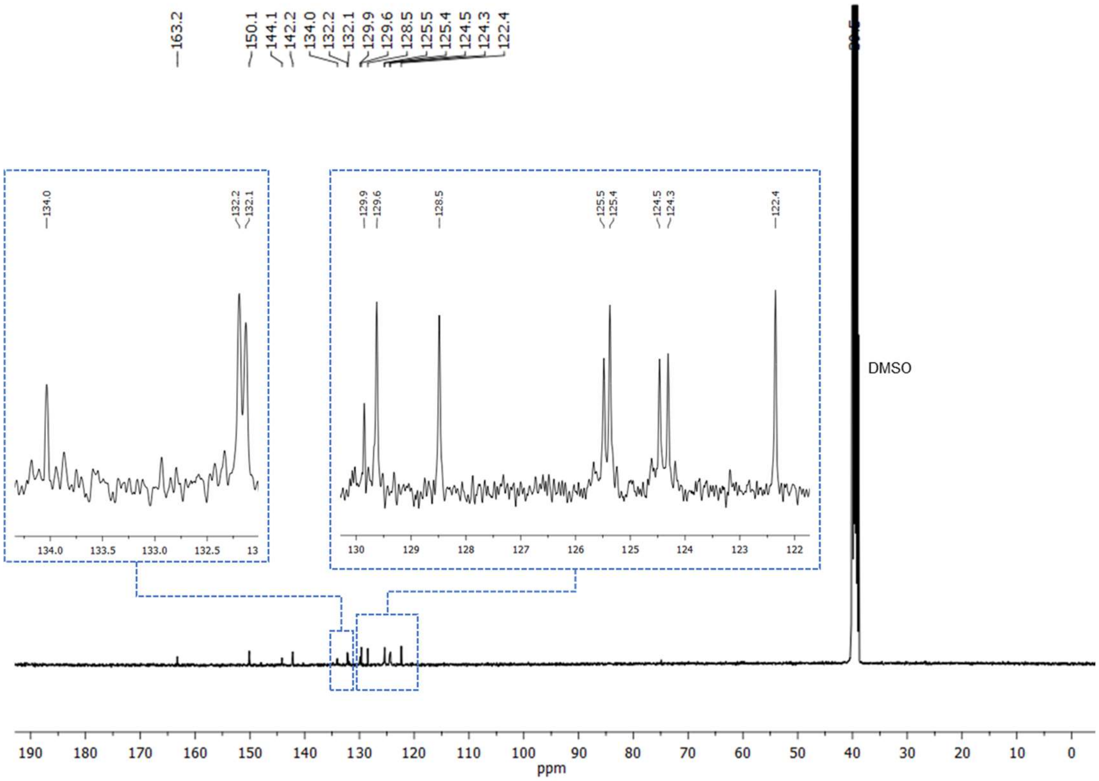

Figure S33. $^{13}C\{^{1}H\}$ NMR spectrum (101 MHz, DMSO-d_6) of **2b**.

Complex 2b’

A solution of compound **2a** (70 mg, 0.11 mmol) in DCM (11 mL) was treated with $PhICl_2$ (33 mg, 0.12 mmol) and stirred for 30 min at room temperature in the dark. The solvent was evaporated under vacuum, and the residue was washed with cold (0 °C) DCM (10 mL × 5) to obtain compound **2b’** as an off-white solid (10 mg, 0.015 mmol, 14% yield). R_f = 0.38 (DCM/hexanes/acetone, 2/2/1, v/v/v). ^{1}H NMR (400 MHz, DMSO-d_6) δ (ppm): 10.22 (*dd*, *J* = 6.3, 1.2 Hz, 1H), 10.02 (*dd*, *J* = 6.0, 1.3 Hz, 1H), 8.60 (*d*, *J* = 8.5 Hz, 1H), 8.53 – 8.35 (*m*, 4H), 8.31 (*td*, *J* = 8.0, 1.3 Hz, 1H), 7.90 – 7.70 (*m*, 4H), 7.64 – 7.38 (*m*, 5H), 6.97 (*t*, *J* = 7.7 Hz, 1H), 6.85 (*d*, *J* = 8.7 Hz, 1H), 6.68 (*d*, *J* = 7.3 Hz, 1H); ^{13}C {^{1}H} NMR (101 MHz, DMSO-d$_6$) δ (ppm): 163.2, 156.1, 152.6, 150.0, 146.8, 141.8, 141.6, 134.0, 133.3, 132.47, 132.43, 131.9, 130.8, 129.9, 129.44, 129.41, 128.1, 128.0, 127.77, 126.74, 126.6, 126.0, 125.6, 125.5, 125.2, 124.7, 124.0, 123.7, 123.5, 122.4. HRMS: [**2b**’ - Cl]$^+$, *m*/*z* = 638.0953 Da (exp), 638.0963 Da (calc).

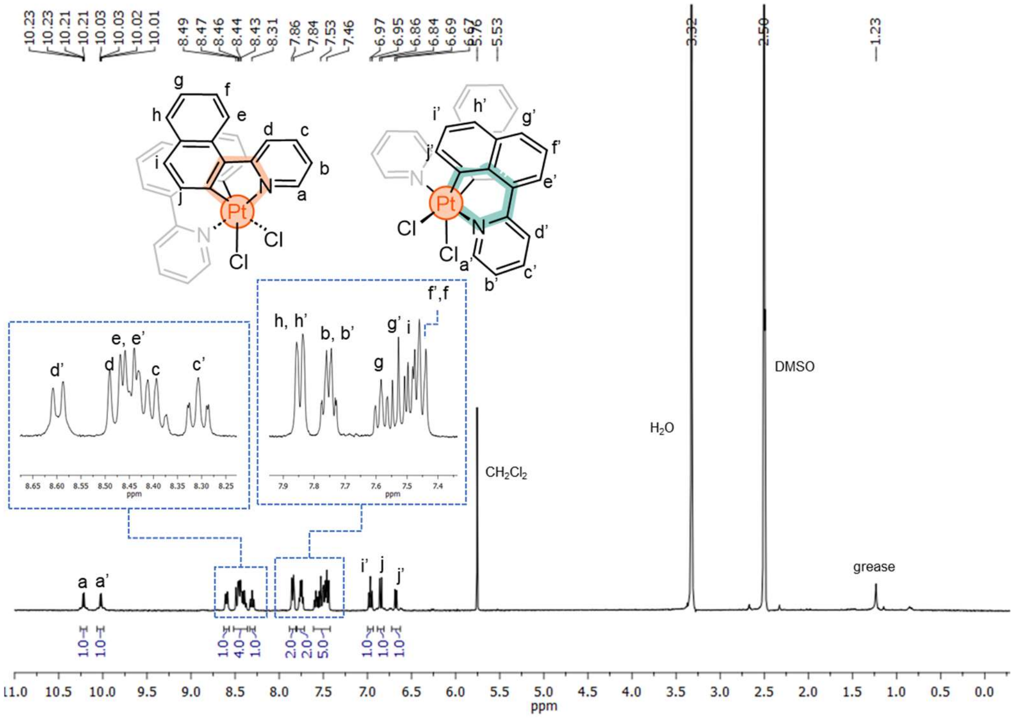

Figure S34. ^{1}H NMR spectrum (400 MHz, DMSO-d_6) of **2b’**. The proton signals corresponding to the ligand that forms a 5-membered metallacycle are labeled a-j; proton signals corresponding to the ligand that forms a 6-membered metallacycle are labeled a'-j'.

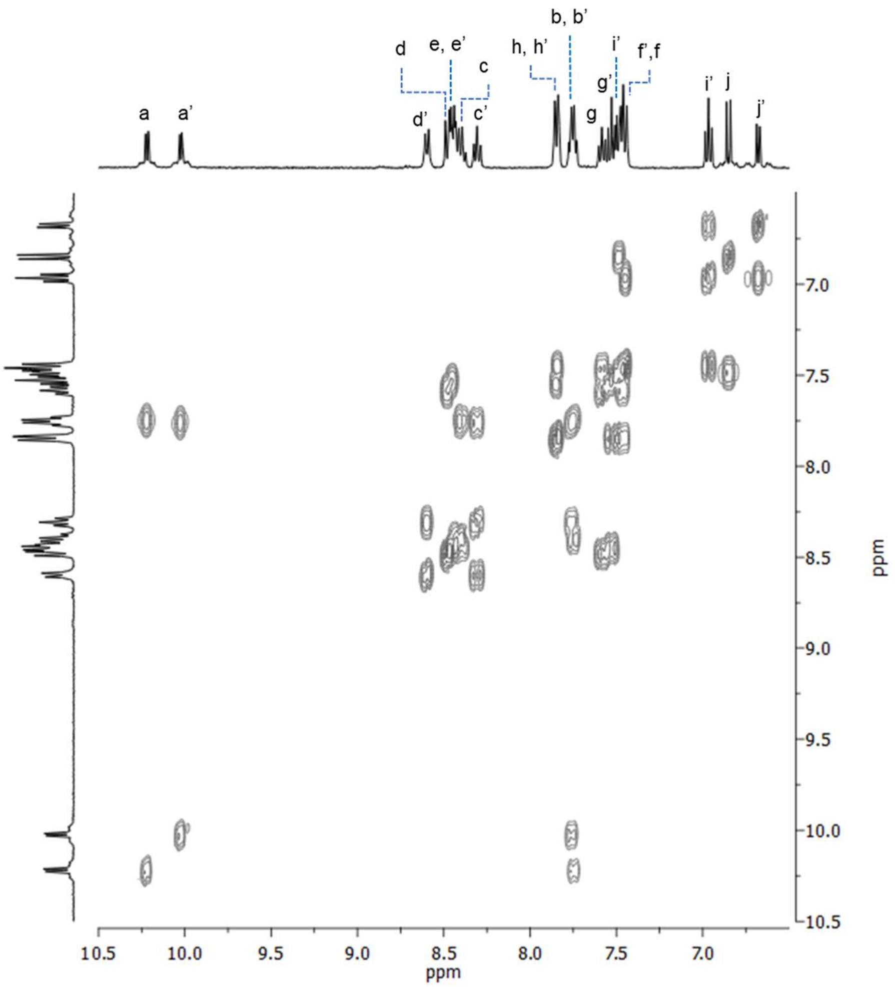

Figure S35. $^1H–^1H$ COSY NMR spectrum (400 MHz, DMSO-d_6) of **2b’**.

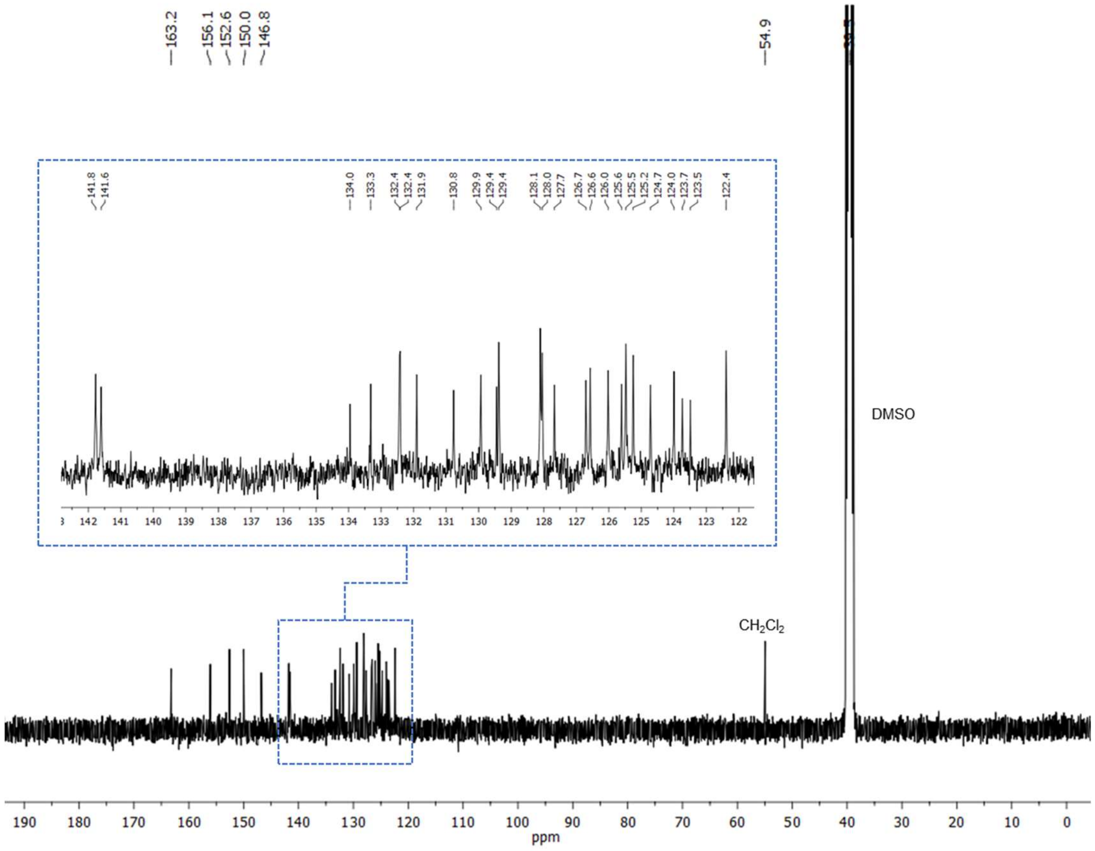

Figure S36. $^{13}C\{^1H\}$ NMR spectrum (101 MHz, DMSO-d_6) of **2b'**.

Compound 3b

A solution of compound **3a** (10 mg, 0.016 mmol) was treated with $PhICl_2$ (4.1 mg, 0.015 mmol) in DCM (3 mL) and stirred for 5 min at room temperature in the dark. The solvent was evaporated under vacuum and the desired product was purified by column chromatography (SiO_2, DCM/hexanes/acetone, 2/2/1, v/v/v, R_f = 0.35), and obtained as a yellow solid (3.4 mg, 4.3 μmol, 29% yield). ^{1}H NMR (400 MHz, DMSO-d_6) δ (ppm): 10.03 (*d*, *J* = 5.5 Hz, 2H), 9.34 (*s*, 2H), 9.13 (*d*, *J* = 8.4 Hz, 2H), 8.57 – 8.49 (*m*, 4H), 8.31 (*d*, *J* = 8.5 Hz, 2H), 8.03 (*d*, *J* = 8.2 Hz, 2H), 7.90 (*t*, *J* = 6.6 Hz, 2H), 7.71 (*d*, *J* = 8.9 Hz, 2H), 7.60 (*t*, *J* = 7.3 Hz 2H), 7.53 (*t*, *J* = 7.0 Hz, 2H), 6.34 (*d*, *J* = 9.1 Hz, 2H). ^{13}C {^{1}H} NMR could not be recorded with enough quality due to the low solubility of the compound in all available deuterated solvents. HRMS: [**3b** - Cl]$^+$, *m*/*z* = 738.1253 Da (exp), 738.1276 Da (calc).

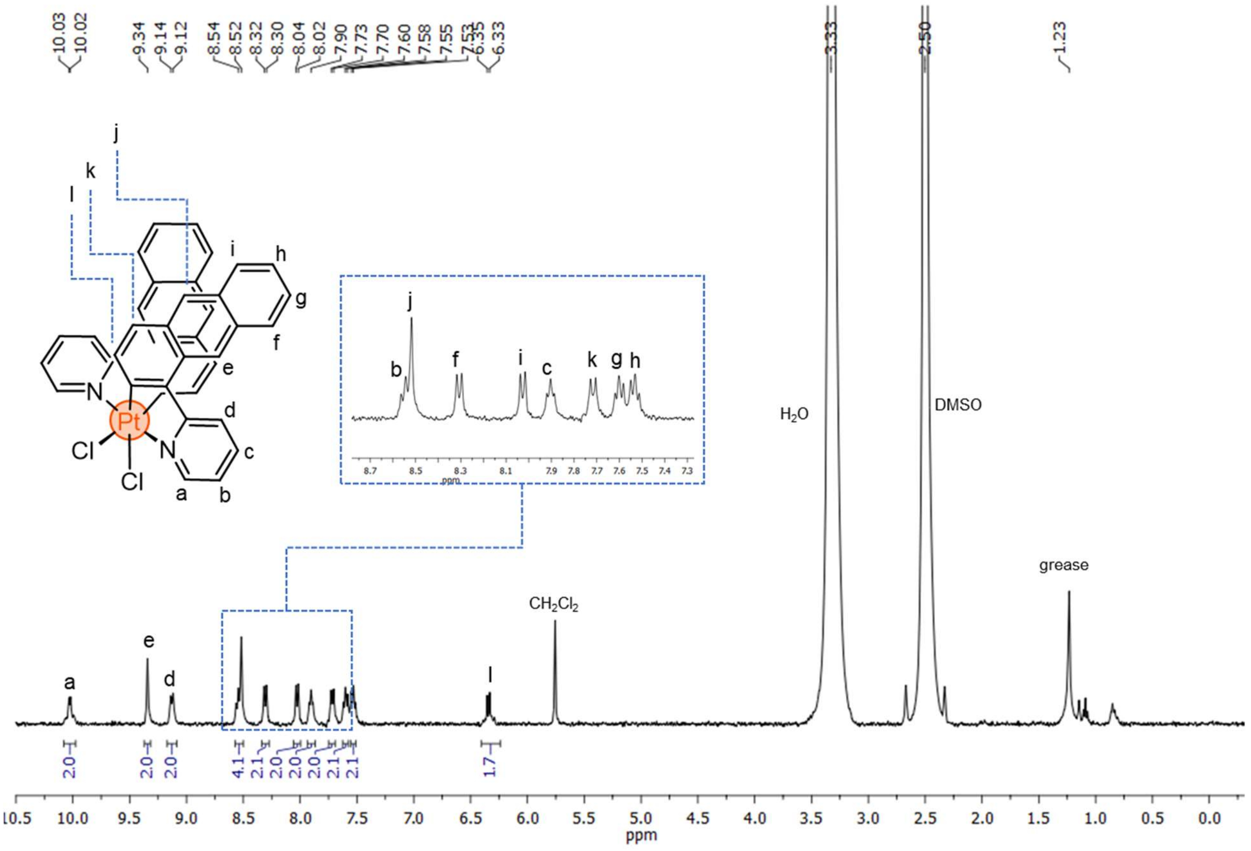

Figure S37. ^{1}H NMR spectrum (400 MHz, DMSO-d_6) of **3b**.

Complex 3b'

A solution of **3a** (10.1 mg, 0.014 mmol) in DCM (3 mL) was treated with $PhICl_2$ (4.1 mg, 0.015 mmol) and stirred for 5 min at room temperature in the dark. The solvent was evaporated under vacuum and the target product **3b'** was obtained as a red solid (5.1 mg, 6.5 μmol, 48% yield) by column chromatography (SiO_2, DCM/hexanes/acetone, 2/2/1, v/v/v, R_f = 0.38). ^{1}H NMR (400 MHz, DCM-d_2) δ (ppm): 10.60 (*dd*, *J* = 6.3, 1.4 Hz, 1H), 10.43 (*dd*, *J* = 6.1, 1.3 Hz, 1H), 9.56 (*d*, *J* = 8.6 Hz, 1H), 8.25 (*s*, 1H), 8.17 (*td*, *J* = 7.7, 1.5, 1H), 8.17 (*s*, 1H), 8.06 – 7.95 (*m*, 3H), 7.90 – 7.77 (*m*, 3H), 7.76 (*s*, 1H), 7.69 – 7.54 (*m*, 3H), 7.46 – 7.37 (*m*, 3H), 7.33 (*d*, *J* = 8.9 Hz, 1H), 7.27 – 7.15 (*m*, 3H), 7.07 (*d*, *J* = 8.9 Hz, 1H); ^{13}C {^{1}H} NMR (101 MHz, DCM-d_2) δ (ppm): 164.3, 157.7, 154.5, 153.4, 149.2, 141.0, 140.6, 135.0, 134.4, 133.15, 133.12, 132.48, 132.45, 132.1, 131.2, 130.7, 130.4, 130.3, 130.0, 129.4, 128.2, 128.1, 127.9, 127.8, 127.6, 126.9, 126.77, 126.75, 126.72, 126.0, 125.0, 124.3, 124.2, 124.0, 123.3, 123.0, 121.7, 120.8. HRMS: [**3b** - Cl]$^+$, *m*/*z* = 738.1273 Da (exp), 738.1276 Da (calc).

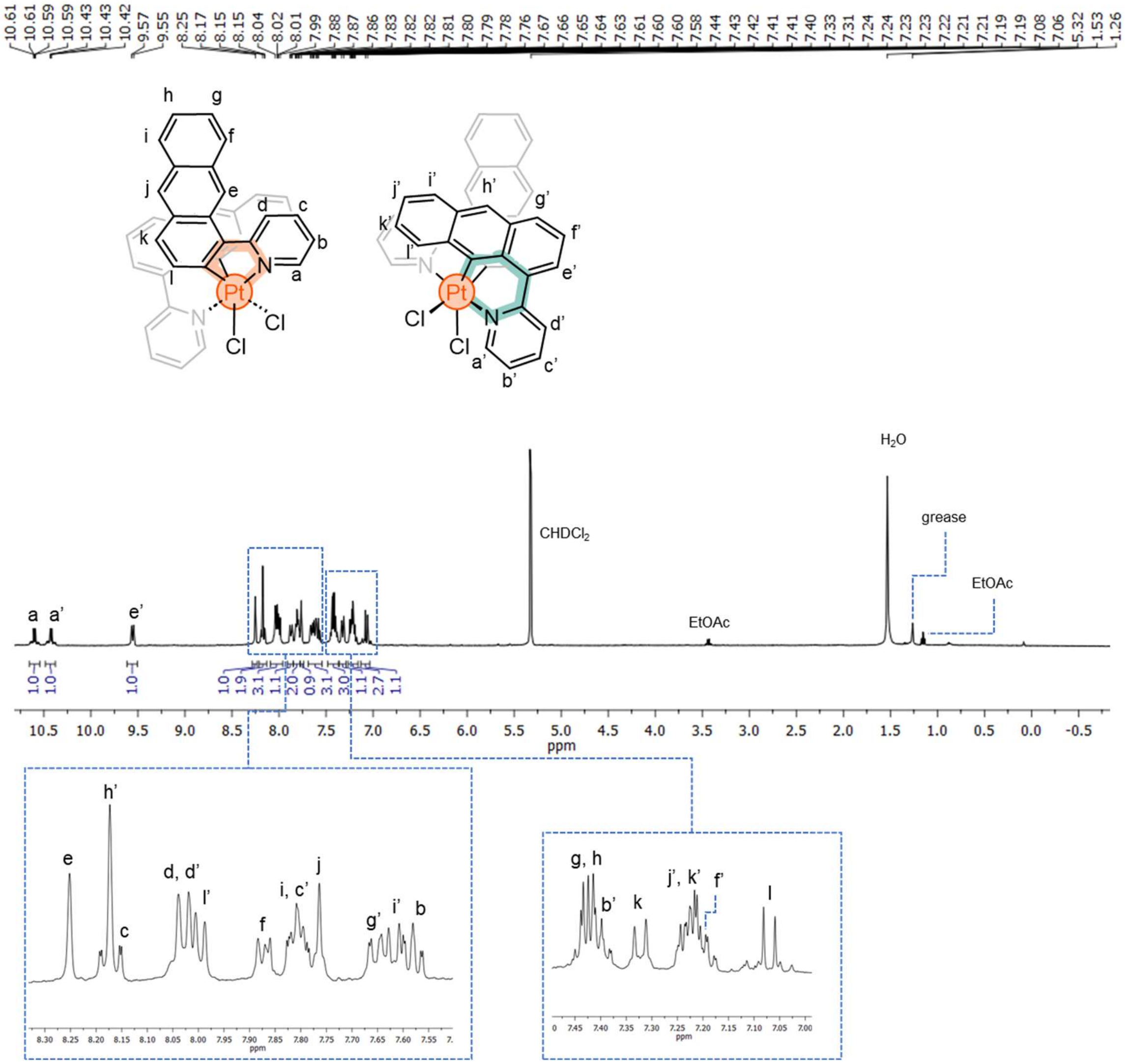

Figure S38. ^{1}H NMR spectrum (400 MHz, DCM-d_2) of **3b'**. The proton signals corresponding to the ligand that forms a 5-membered metallacycle are labeled a-l; proton signals corresponding to the ligand that forms a 6-membered metallacycle are labeled a'-l'.

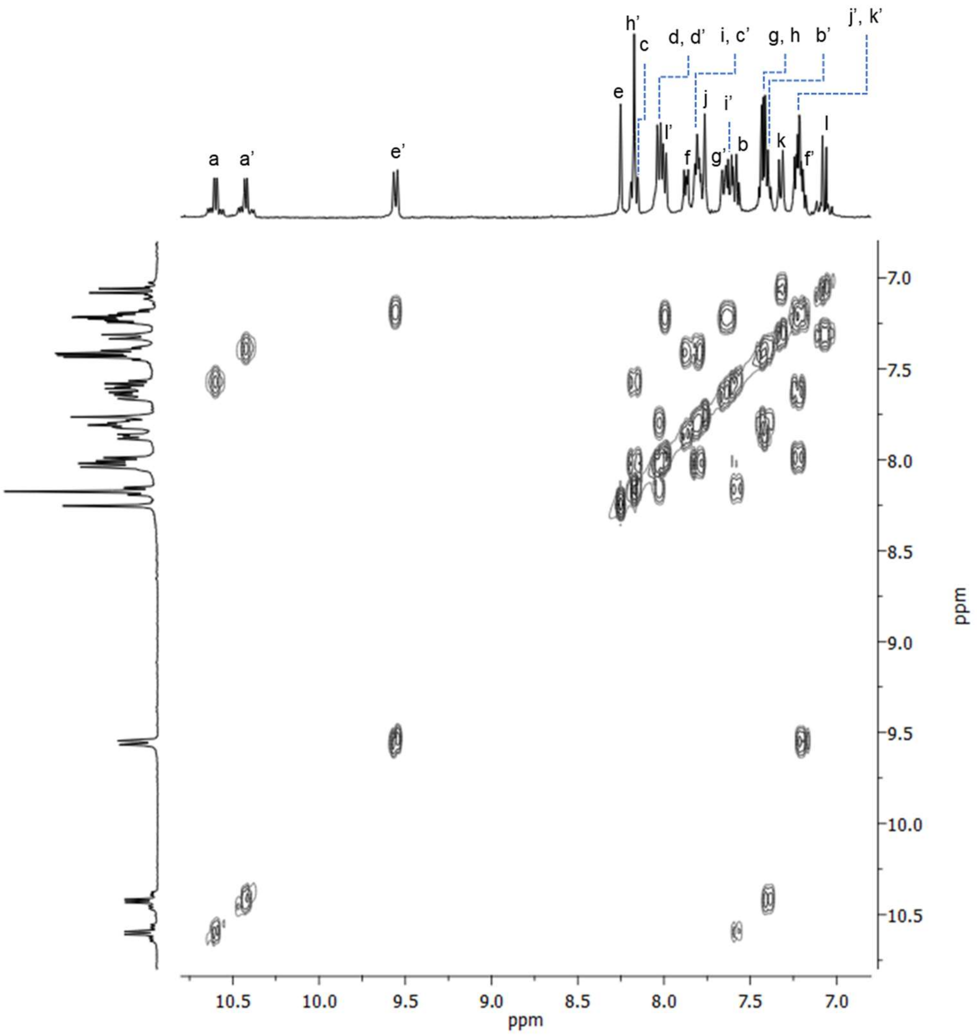

Figure S39. ^{1}H–^{1}H COSY NMR spectrum (400 MHz, DCM-d_2) of **3b'**.

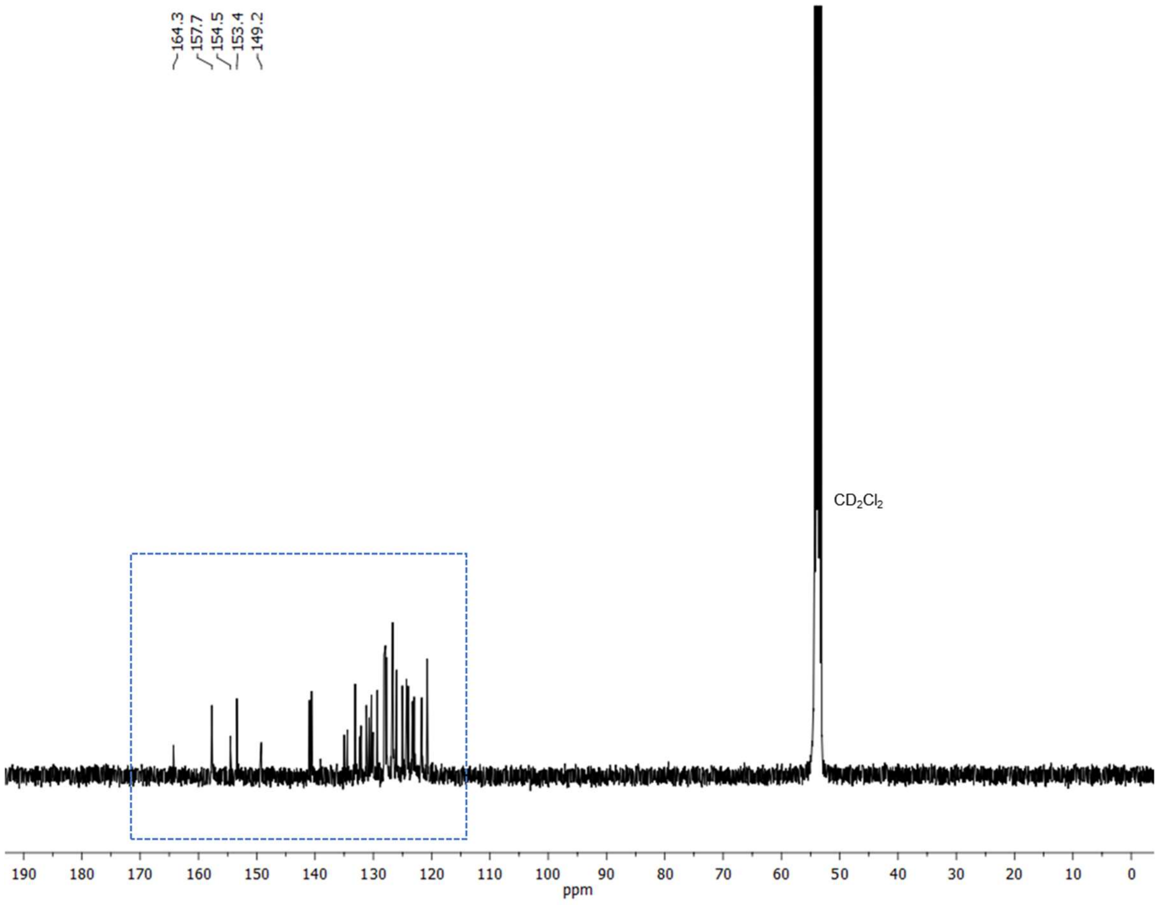

Figure S40. $^{13}C\{^{1}H\}$ NMR spectrum (101 MHz, DCM-d_2) of **3b’**.

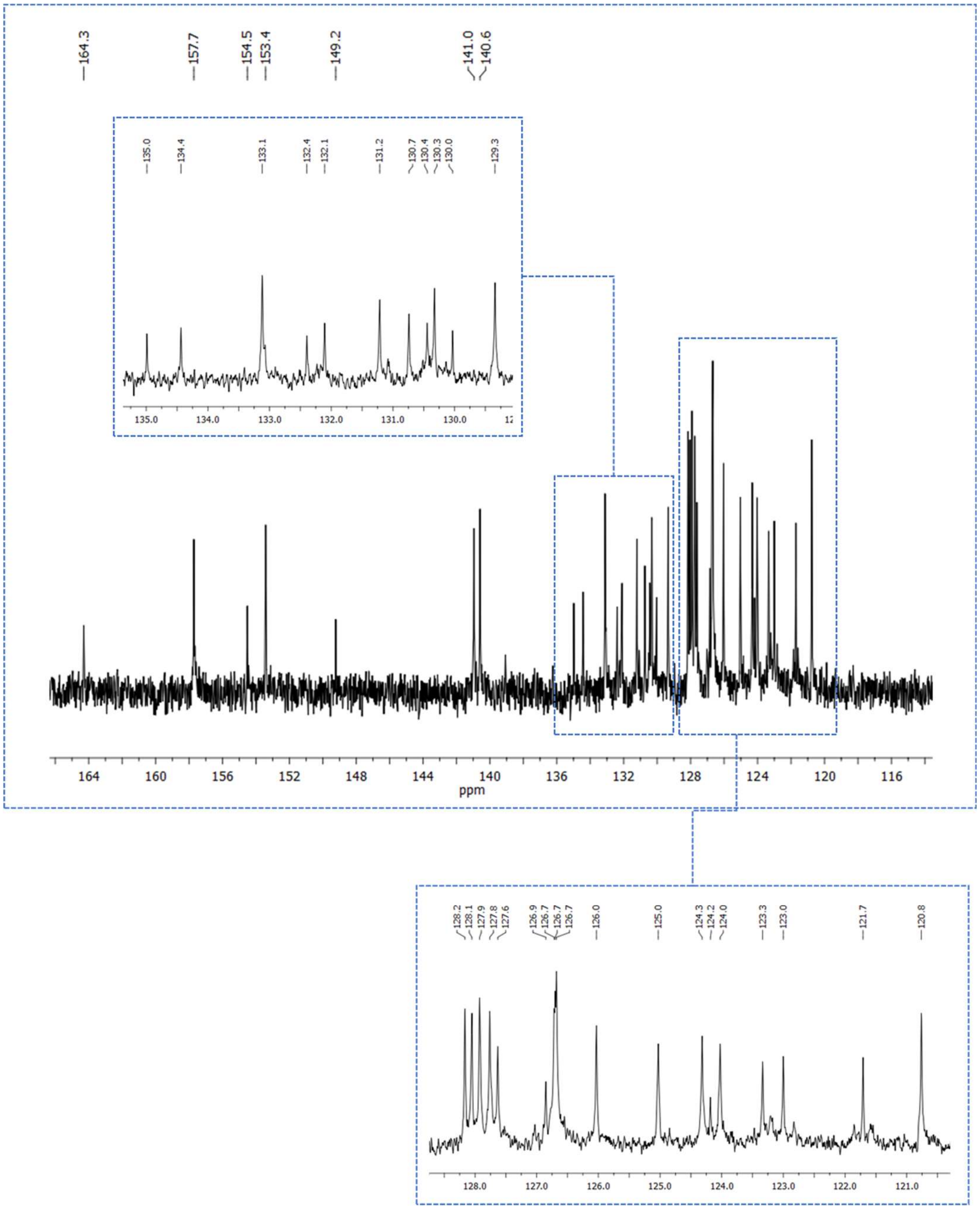

Figure S41. Zoomed-in $^{13}C\{^{1}H\}$ NMR spectrum (101 MHz, DCM-d_2) of **3b'**.

Complex 2c

A solution of compound **2b** (12.6 mg, 0.018 mmol) prepared in dry THF (2.0 mL) was mixed with *t*-BuOK (42.9 mg, 0.38 mmol) under the protection of N_2. The resulting suspension was heated at 60 °C and stirred under N_2 for 1 d. After cooling down to room temperature, the solvent was evaporated under vacuum (T < 35 °C) and the residue was partitioned between H_2O (10 mL) and DCM (10 mL). The aqueous phase was extracted with DCM (3 × 10 mL) and all organic layers were combined, dried over anhydrous Na_2SO_4, and concentrated by rotatory evaporation. The desired product was purified by column chromatography (SiO_2, DCM/hexanes/acetone, 8/8/1, v/v/v, R_f = 0.32), and obtained as a deep yellow solid (5.4 mg, 8.9 μmol, 49% yield). ^{1}H NMR (400 MHz, DCM-d_2) δ (ppm): 8.92 (*d*, *J* = 5.2 Hz, 2H), 8.49 (*d*, *J* = 8.6 Hz, 2H), 8.42 (*d*, *J* = 8.4 Hz, 2H), 8.32 (*d*, *J* = 8.2 Hz, 2H), 7.97 (*td*, *J* = 7.8,1.2, 2H), 7.88 (*d*, *J* = 7.8 Hz, 2H), 7.74 (*d*, *J* = 8.4 Hz, 2H), 7.52 (*td*, *J* = 8.5, 1.4 Hz, 2H), 7.42 (*td*, *J* = 7.4, 0.9, 2H), 7.36 (*t*, *J* = 6.1 Hz, 2H); ^{13}C {^{1}H} NMR (101 MHz, DCM-d_2) δ (ppm): 167.1, 153.4, 149.0, 141.3, 138.1, 136.0, 132.3, 130.7, 129.6, 129.3, 127.0, 124.5, 123.9, 122.7, 122.2. HRMS: [**2c** + H]$^+$, m/z = 604.1342 Da (exp), 604.1353 Da (calc).

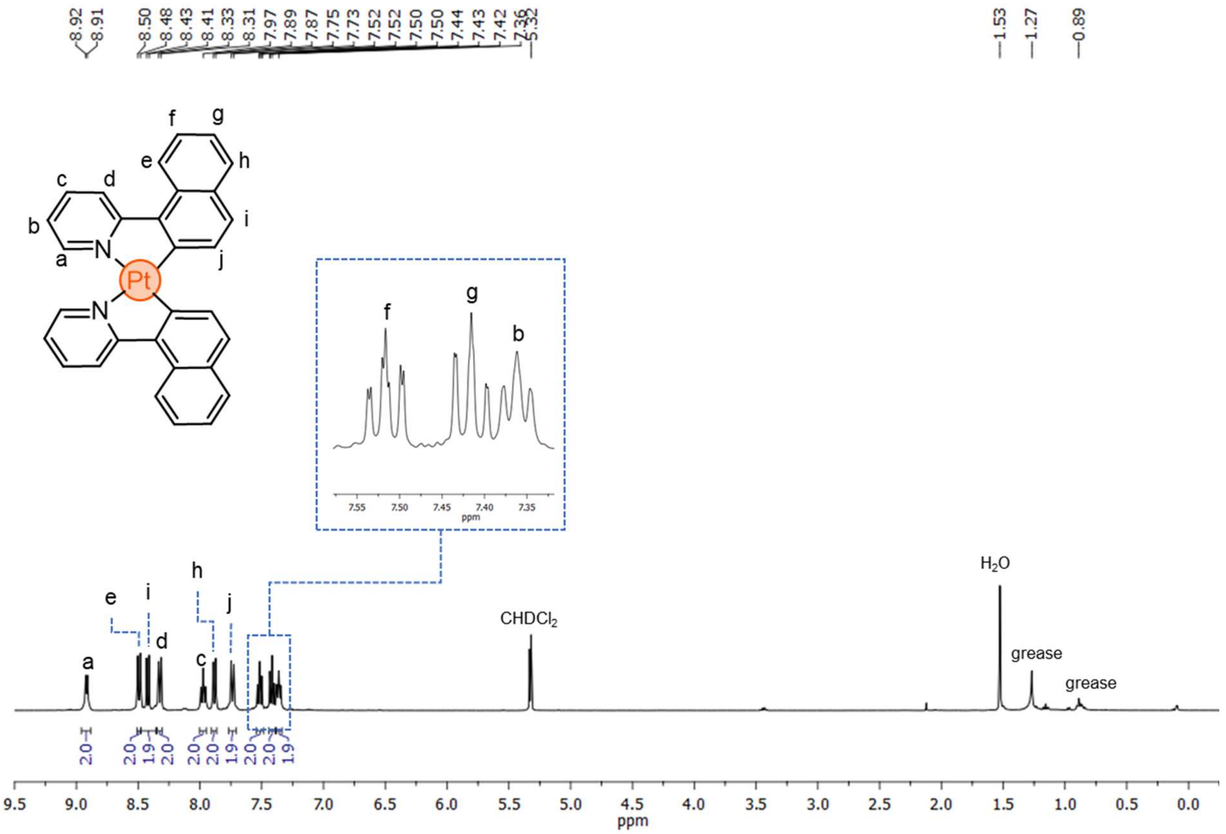

Figure S42. ^{1}H NMR spectrum (400 MHz, DCM-d_2) of **2c**

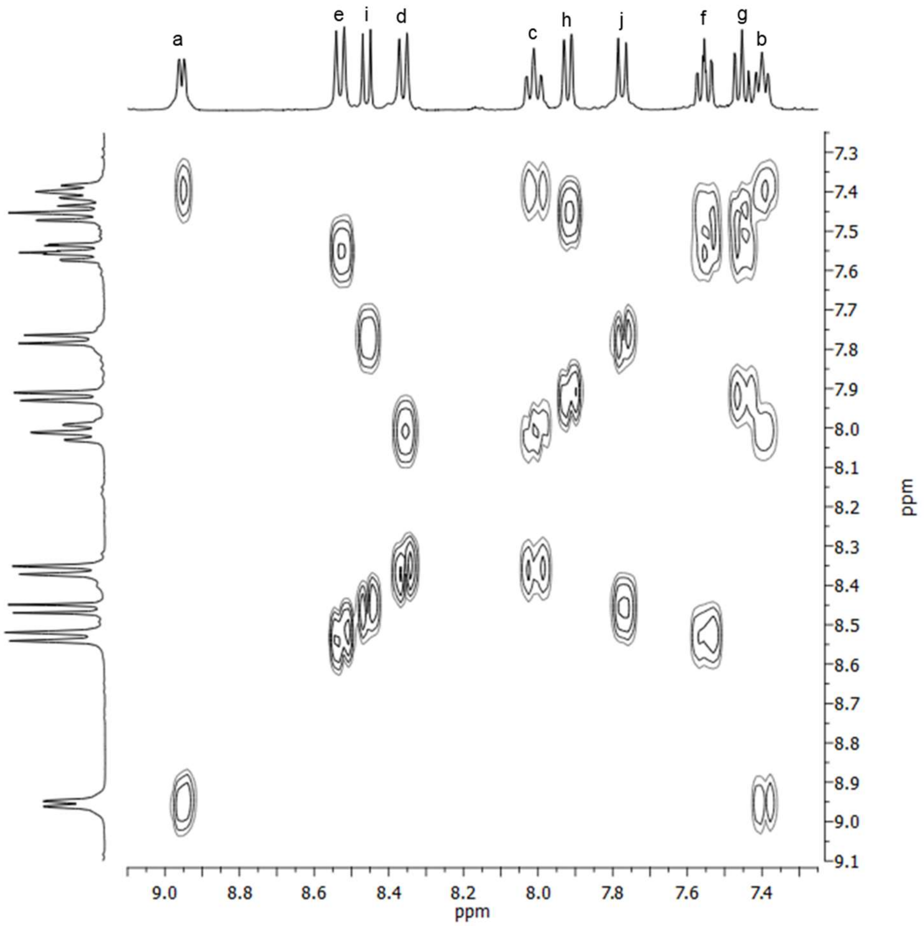

Figure S43. ^{1}H–^{1}H COSY NMR spectrum (400 MHz, DCM-d_2) of **2c**.

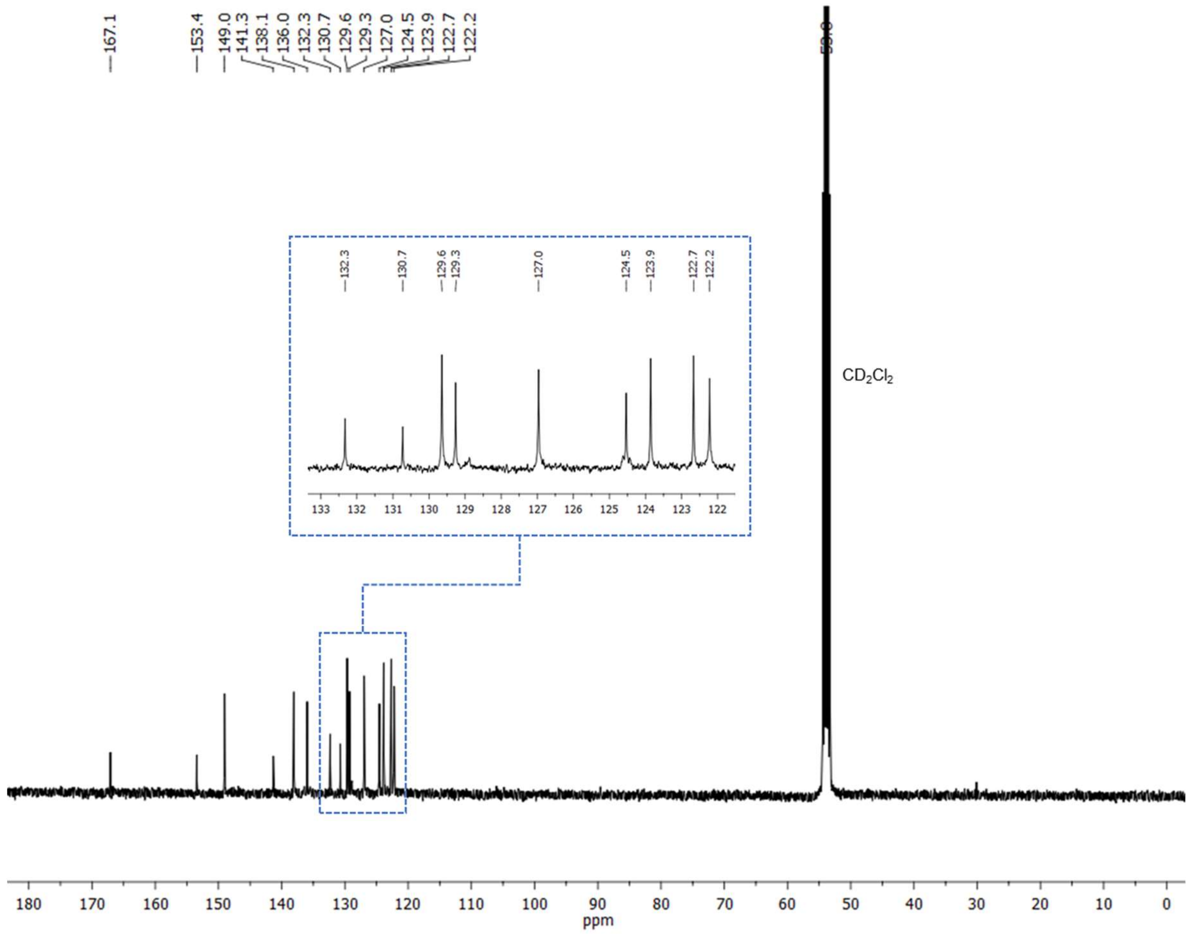

Figure S44. $^{13}C\{^{1}H\}$ NMR spectrum (101 MHz, DCM-d_2) of **2c**.

Complex 2c'

A solution of **2b'** (12.1 mg, 0.017 mmol) in dry THF (2.0 mL) was mixed with *t*-BuOK (40.3 mg, 0.36 mmol) under the protection of N_2. The resulting suspension was heated at 60 °C and stirred under N_2 for 1 d. After cooling the system down to room temperature, the solvent was evaporated under vacuum (T < 35 °C), and the residue was partitioned between H_2O (10 mL) and DCM (10 mL). The aqueous phase was extracted with DCM (3 × 10 mL) and all organic layers were combined, dried over anhydrous Na_2SO_4, and concentrated by rotatory evaporation. The product (**2c'**) was purified by column chromatography (SiO_2, DCM/hexanes/acetone, 8/8/1, v/v/v, R_f = 0.30) and obtained as a deep yellow solid (5.3 mg, 8.8 μmol, 49% yield). ^{1}H NMR (400 MHz, DCM-d_2) δ (ppm): 9.04 (*dd*, *J* = 5.6, 1.0 Hz, 1H), 8.46 (*d*, *J* = 8.6 Hz, 1H), 8.28 (*d*, *J* = 8.3 Hz, 1H), 8.17 – 8.05 (*m*, 2H), 8.00 – 7.90 (*m*, 3H), 7.85 (*td*, *J* = 8.0, 1.6 Hz, 1H), 7.80 (*d*, *J* = 7.8 Hz, 1H), 7.77 (*dd*, *J* = 6.9, 0.9 Hz, 1H), 7.57 (*dd*, *J* = 7.9, 0.8 Hz, 1H), 7.49 (*td*, *J* = 7.6, 1.2 Hz, 1H), 7.46 (*d*, *J* = 7.8 Hz, 1H), 7.44 (*d*, *J* = 6.6 Hz, 1H), 7.40 – 7.30 (*m*, 3H), 7.27 (*d*, *J* = 8.4 Hz, 1H), 7.10 (*td*, *J* = 6.6, 1.2 Hz ,1H); ^{13}C {^{1}H} NMR (101 MHz, DCM-d_2) δ (ppm): 167.0, 157.4, 156.1, 152.3, 148.2, 144.6, 142.0, 139.1, 138.2, 138.1, 136.1, 135.8, 135.1, 132.6, 132.4, 132.2, 130.7, 129.3, 127.2, 126.5, 126.4, 126.2, 124.5, 123.6, 123.34, 123.31, 123.2, 130.0, 122.4, 121.2. HRMS: [**2c'** + H]$^+$, *m*/*z* = 604.1342 Da (exp), 604.1353 Da (calc).

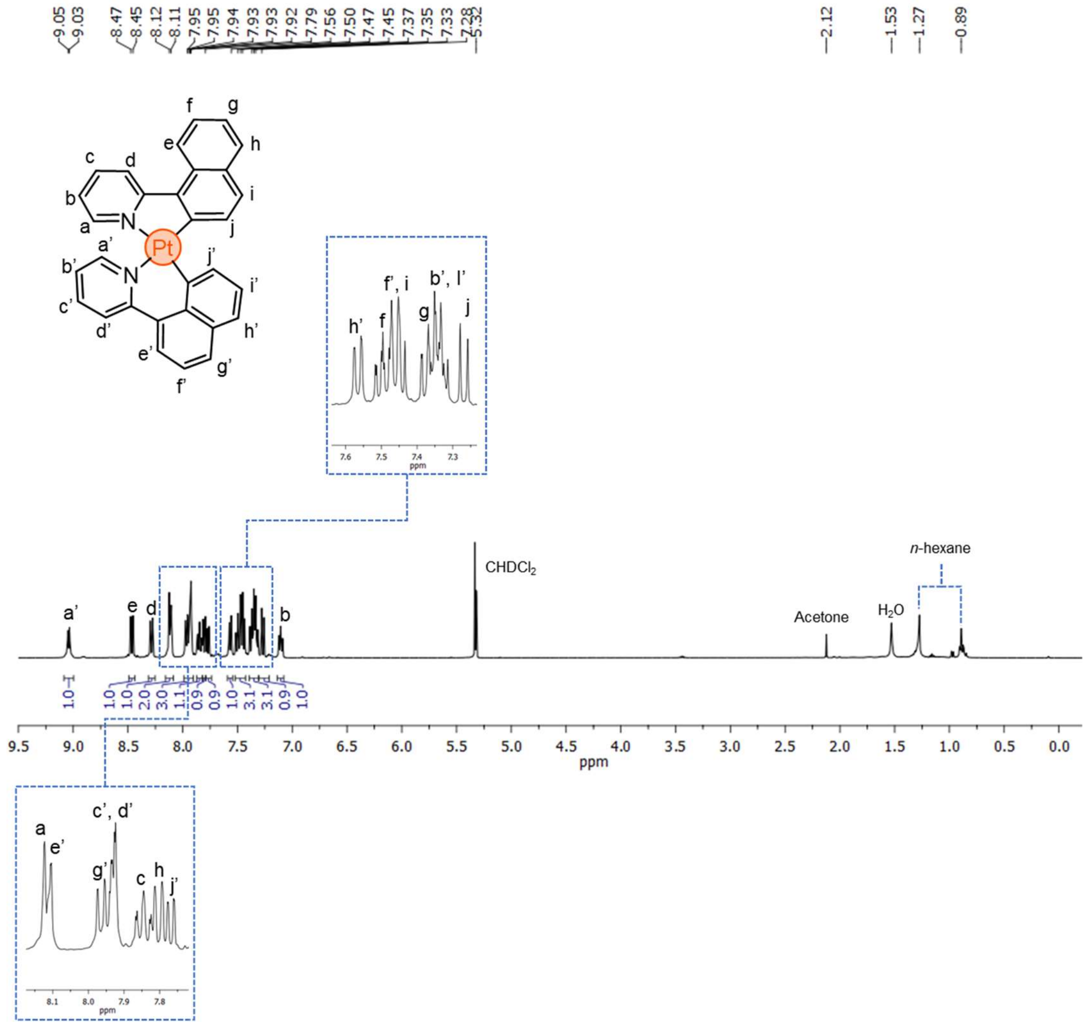

Figure S45. ^{1}H NMR spectrum (400 MHz, DCM-d_2) of **2c'**.

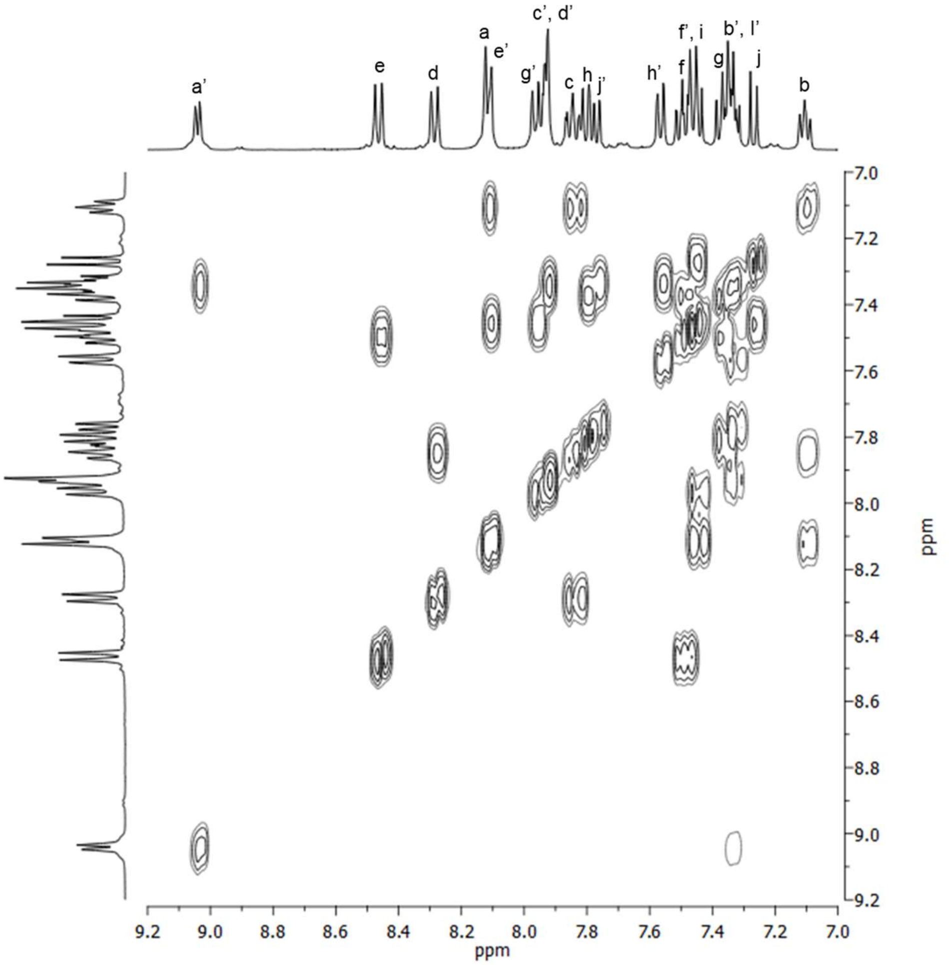

Figure S46. ^{1}H–^{1}H COSY NMR spectrum (400 MHz, DCM-d_2) of **2c'**.

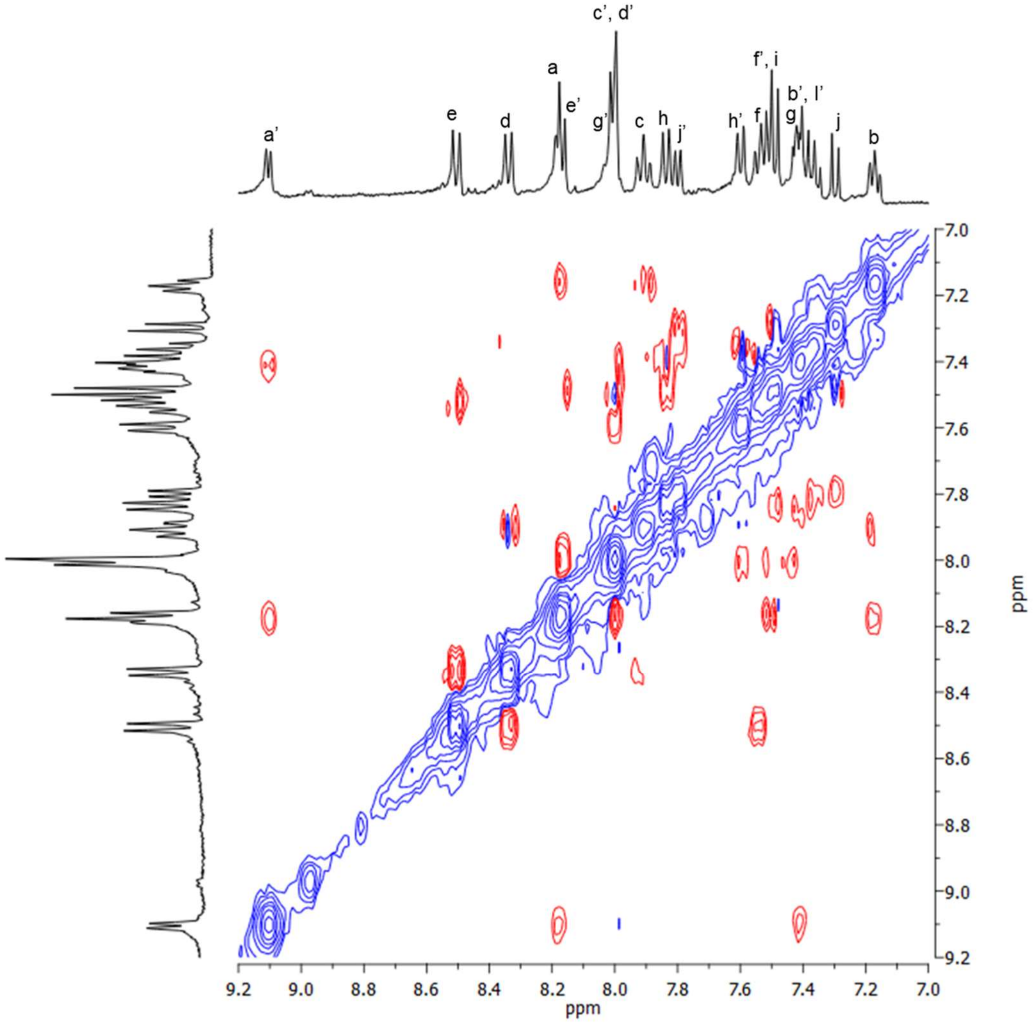

Figure S47. ^{1}H–^{1}H NOESY NMR spectrum (400 MHz, DCM-d_2) of **2c'**.

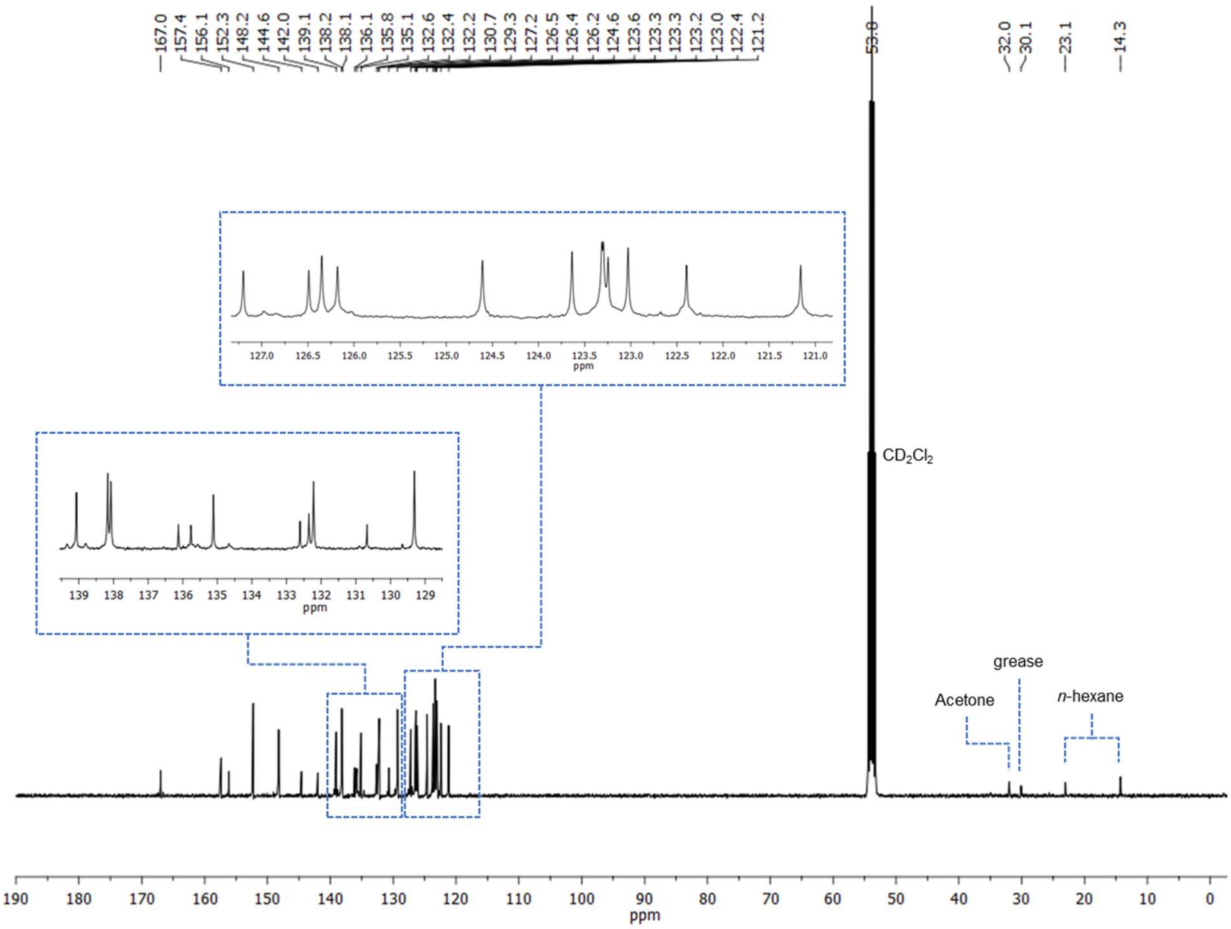

Figure S48. $^{13}C\{^1H\}$ NMR spectrum (101 MHz, DCM-d_2) of **2c'**.

Pt^{II} complex 3c

A solution of precursor **3b** (5.0 mg, 6.45 μmol) prepared in dry THF (1.0 mL) was mixed with *t*-BuOK (14.5 mg, 0.13 mmol) under the protection of a N_2 atmosphere. The resulting suspension was heated at 60 °C and stirred under N_2 for 1 d. After cooling down to room temperature, the solvent was evaporated under vacuum (T < 35 °C) and the residue was partitioned between H_2O (10 mL) and DCM (10 mL). The aqueous phase was extracted with DCM (3 × 10 mL) and all organic layers were combined, dried over anhydrous Na_2SO_4, and concentrated by rotatory evaporation. The desired product (**3c**) was purified by column chromatography (SiO_2, DCM/hexanes/acetone, 8/8/1, v/v/v, R_f = 0.29) and obtained as a deep yellow solid (2.3 mg, 3.27 μmol, 51% yield). ^{1}H NMR (400 MHz, DCM-d_2) δ (ppm): 9.07 (*s*, 2H), 8.97 (*dd*, *J* = 5.5, 0.9 Hz, 2H), 8.56 (*d*, *J* = 8.3 Hz, 2H), 8.46 (*s*, 2H), 8.37 (*d*, *J* = 10.0 Hz, 2H), 8.10 – 7.98 (*m*, 6H), 7.93 (*d*, *J* = 8.6 Hz, 2H), 7.52 – 7.46 (*m*, 4H), 7.34 (*td*, *J* = 6.4, 1.2 Hz, 2H); ^{13}C {^{1}H} NMR (101 MHz, DCM-d_2) δ (ppm): 167.3, 155.6, 149.2, 140.2, 138.4, 136.0, 132.8, 131.4, 130.5, 129.4, 129.2, 128.6, 128.4, 127.7, 126.0, 125.2, 124.3, 122.1, 120.4. HRMS: [**3c** + H]$^+$, m/z = 704.1662 Da (exp), 704.1666 Da (calc).

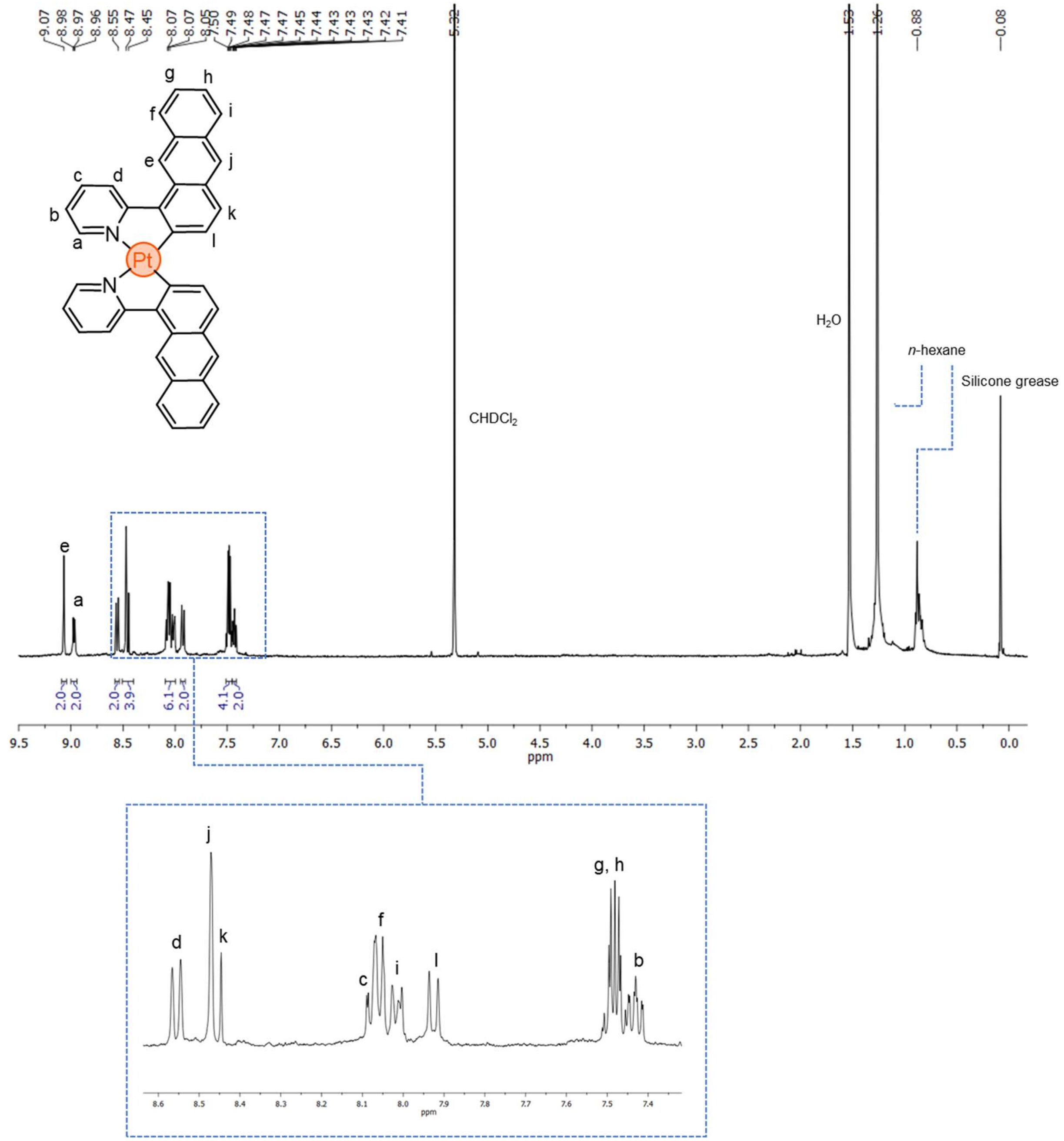

Figure S49. ^{1}H NMR spectrum (400 MHz, DCM-d_2) of **3c**.

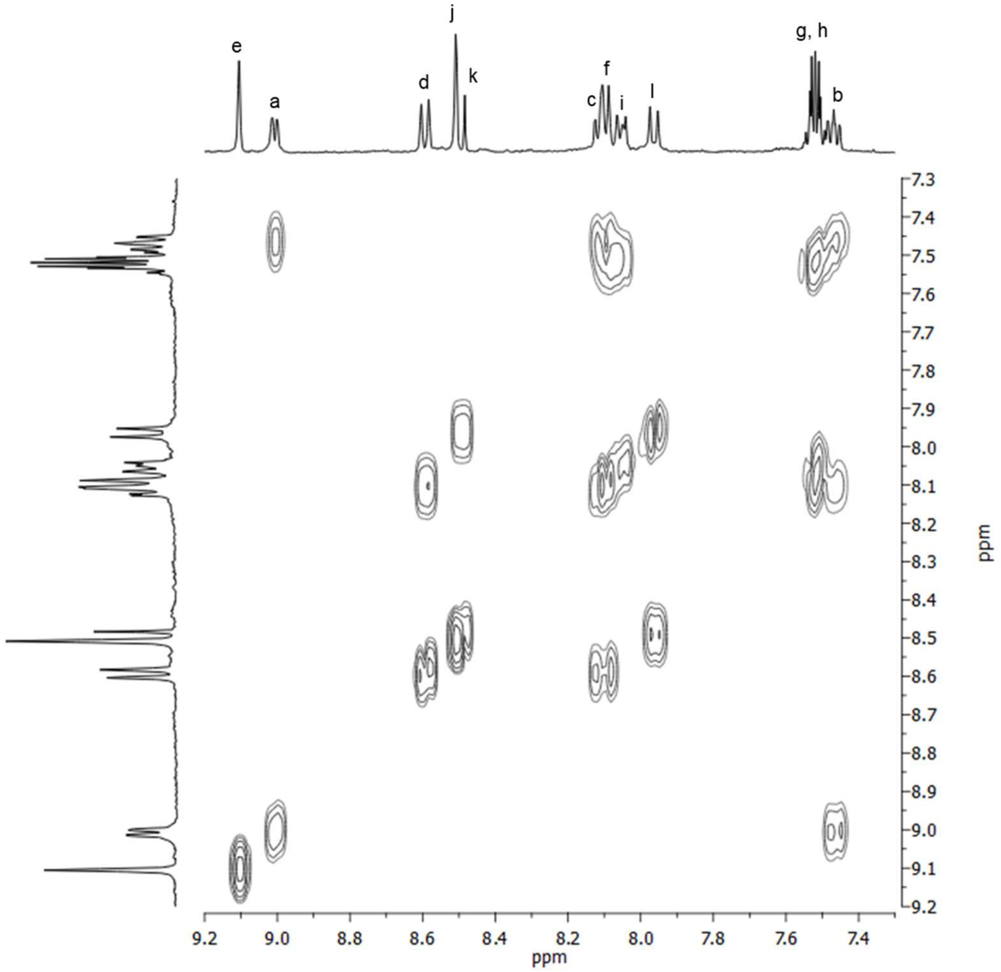

Figure S50. $^1H–^1H$ COSY NMR spectrum (400 MHz, DCM-d_2) of **3c**.

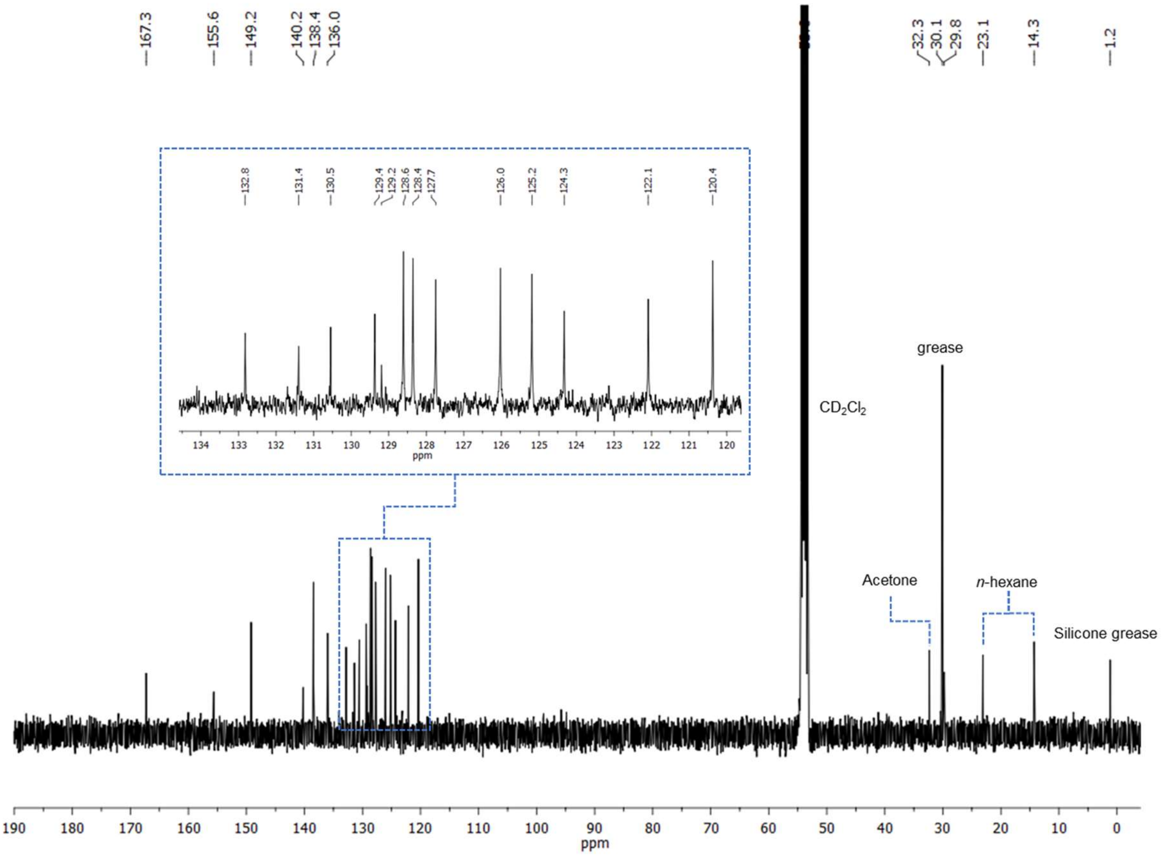

Figure S51. $^{13}C\{^{1}H\}$ NMR spectrum (101 MHz, DCM-d_2) of **3c**.

Pt^{II} complex 3c’

A solution of **3b’** (13.6 mg, 0.017 mmol) prepared in dry THF (1.0 mL) was mixed with *t*-BuOK (39.4 mg, 0.35 mmol) under the protection of a N_2 atmosphere. The resulting suspension was heated at 60 °C and stirred under N_2 for 1 d. After cooling down to room temperature, the solvent was evaporated under vacuum ($T < 35$ °C) and the residue was partitioned between H_2O (10 mL) and DCM (10 mL). The aqueous layer was extracted with DCM (3 × 10 mL) and all organic layers were combined, dried over anhydrous Na_2SO_4, and concentrated by rotatory evaporation. The desired product **3c’** was purified by column chromatography (SiO_2, DCM/hexanes/acetone, 8/8/1, v/v/v, $R_f = 0.27$) and obtained as a deep yellow solid (5.8 mg, 8.2 μmol, 47% yield). ^{1}H NMR (600 MHz, DCM-d_2) δ (ppm): 9.12 (*d*, *J* = 5.2 Hz, 1H), 8.98 (*s*, 1H), 8.52 (*d*, *J* = 8.3 Hz, 1H), 8.24 (*d*, *J* = 8.8 Hz, 1H), 8.21 (*dd*, *J* = 5.6, 1.2 Hz, 1H), 8.17 (*d*, *J* = 8.3 Hz, 1H), 8.15 (*d*, *J* = 6.0 Hz, 1H), 8.15 (*d*, *J* = 6.0 Hz, 1H), 8.13 (*d*, *J* = 6.9 Hz, 1H), 8.04 (*d*, *J* = 8.3 Hz, 1H), 8.99 – 7.91 (*m*, 4H), 7.90 (*d*, *J* = 8.3 Hz, 1H), 7.47 – 7.39 (*m*, 4H), 7.31 (*t*, *J* = 7.4, 1.1 Hz, 1H), 7.17 (*td*, *J* = 6.5, 1.1 Hz, 1H), 7.04 (*td*, *J* = 6.5, 1.1 Hz, 1H), 6.99 (*d*, *J* = 8.6 Hz, 1H), 5.82 (*d*, *J* = 8.6 Hz, 1H); ^{13}C {^{1}H} NMR (151 MHz, DCM-d_2) δ (ppm): 167.2, 158.0, 157.4, 154.6, 152.0, 147.8, 140.3, 140.1, 138.4, 138.1, 137.7, 137.4, 136.7, 133.3, 133.2, 132.3, 132.0, 131.3, 131.2, 130.3, 129.2, 128.5, 128.2, 127.5, 127.1, 126.3, 126.1, 126.0, 125.8, 125.3, 125.0, 123.6, 122.8, 122.3, 121.6, 120.9, 120.4, 119.7. HRMS: [**3c’** + H]$^+$, *m*/*z* = 704.1662 Da (exp), 704.1666 Da (calc).

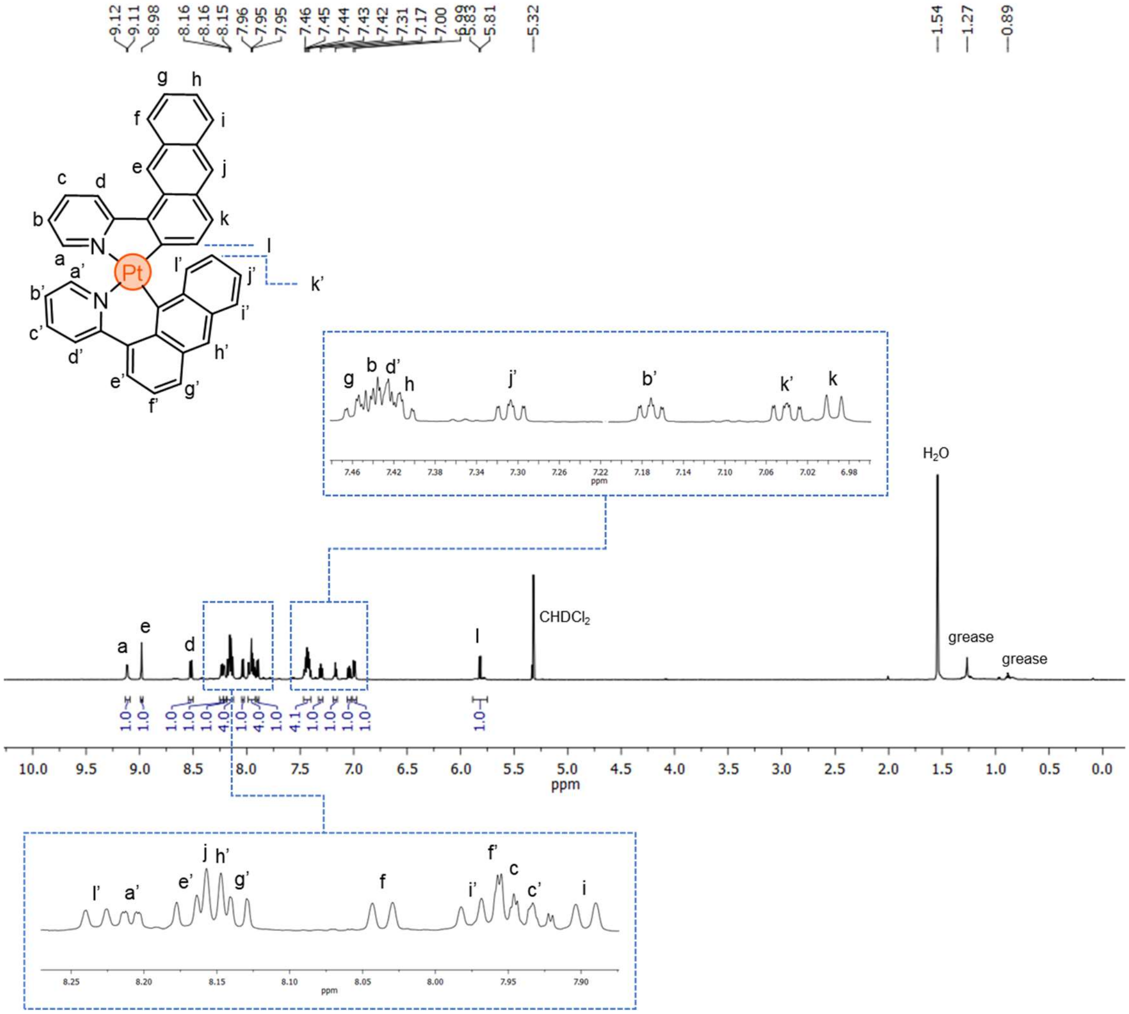

Figure S52. ^{1}H NMR spectrum (600 MHz, DCM-d_2) of **3c'**.

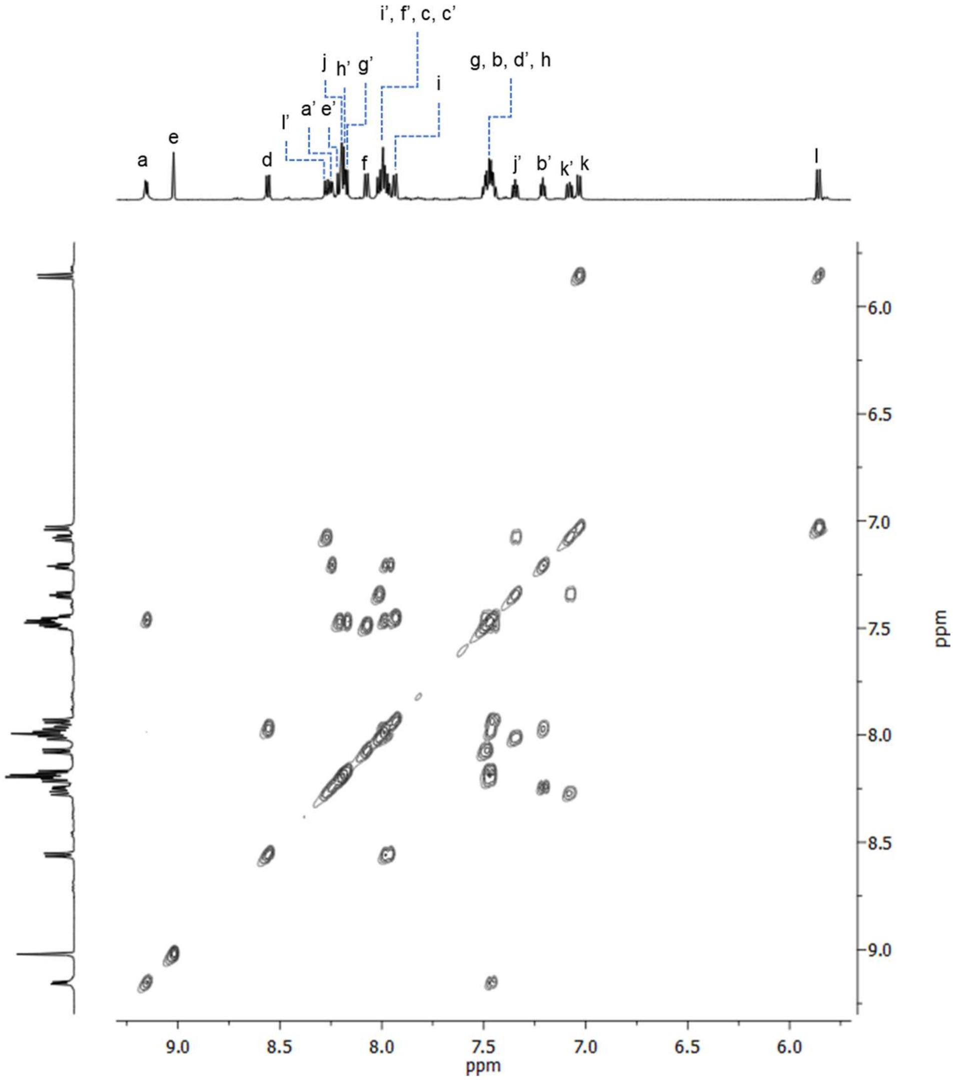

Figure S53. ^{1}H–^{1}H COSY NMR spectrum (600 MHz, DCM-d_2) of **3c'**.

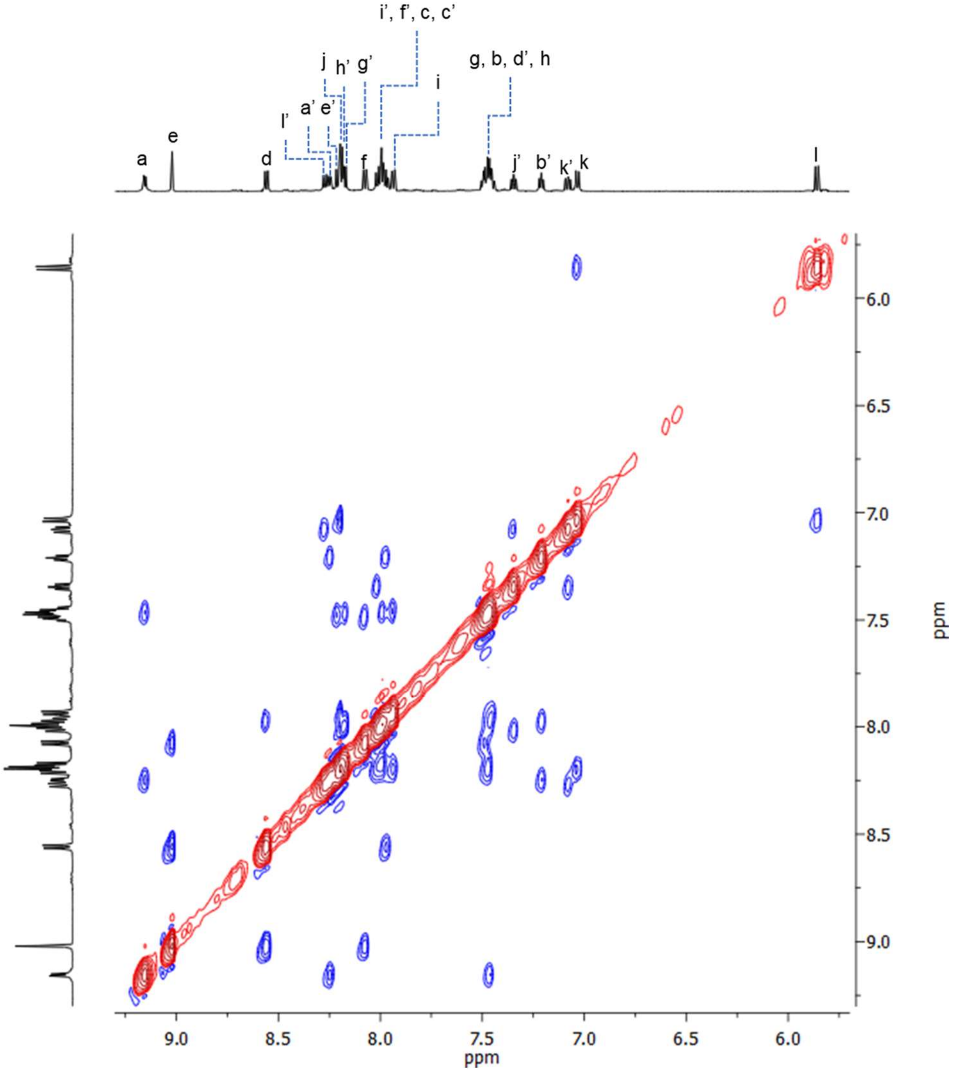

Figure S54. ^1H–^1H NOESY NMR spectrum (600 MHz, DCM-d_2) of **3c'**.

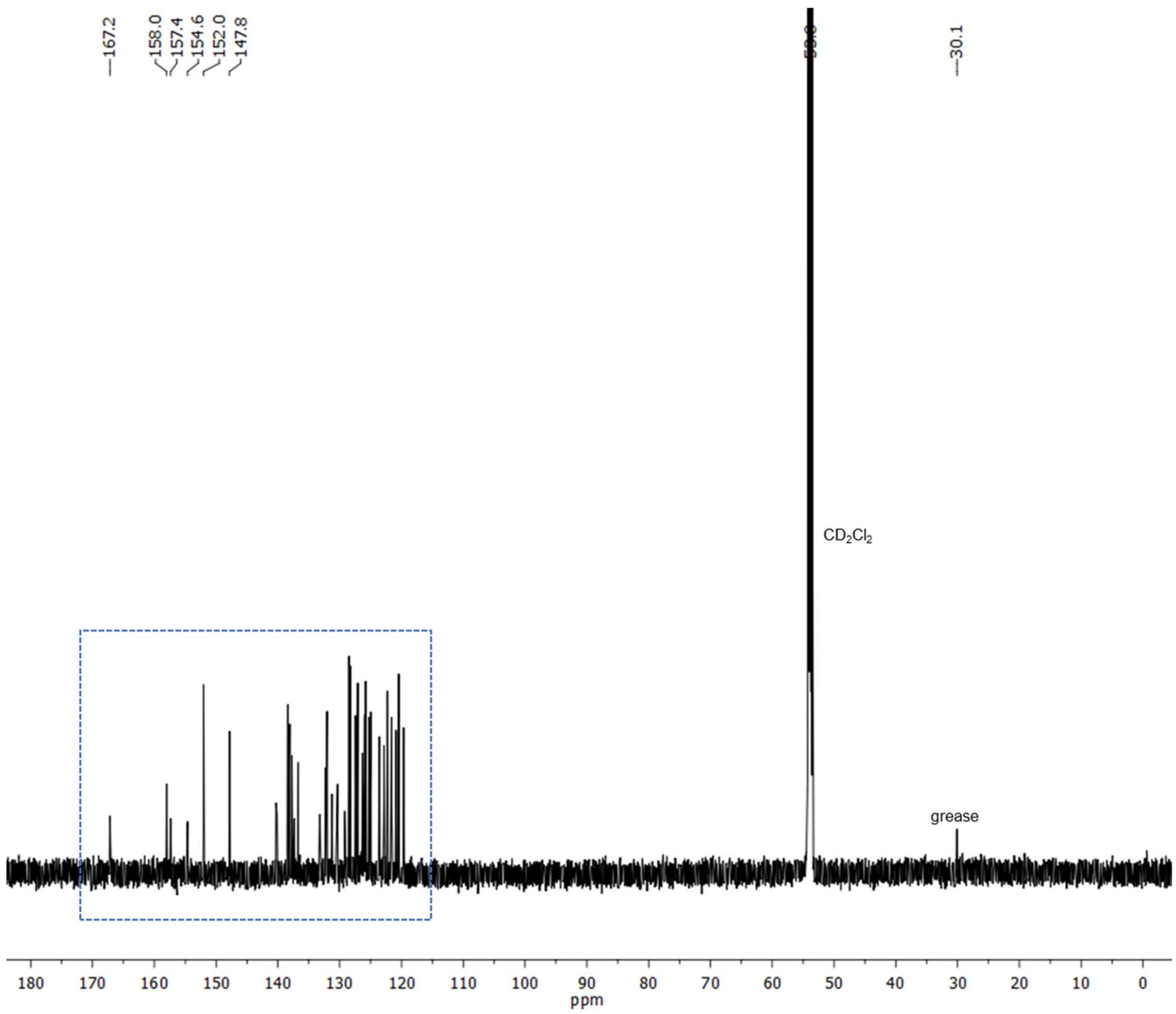

Figure S55. $^{13}C\{^1H\}$ NMR spectrum (151 MHz, DCM-d_2) of **3c’**.

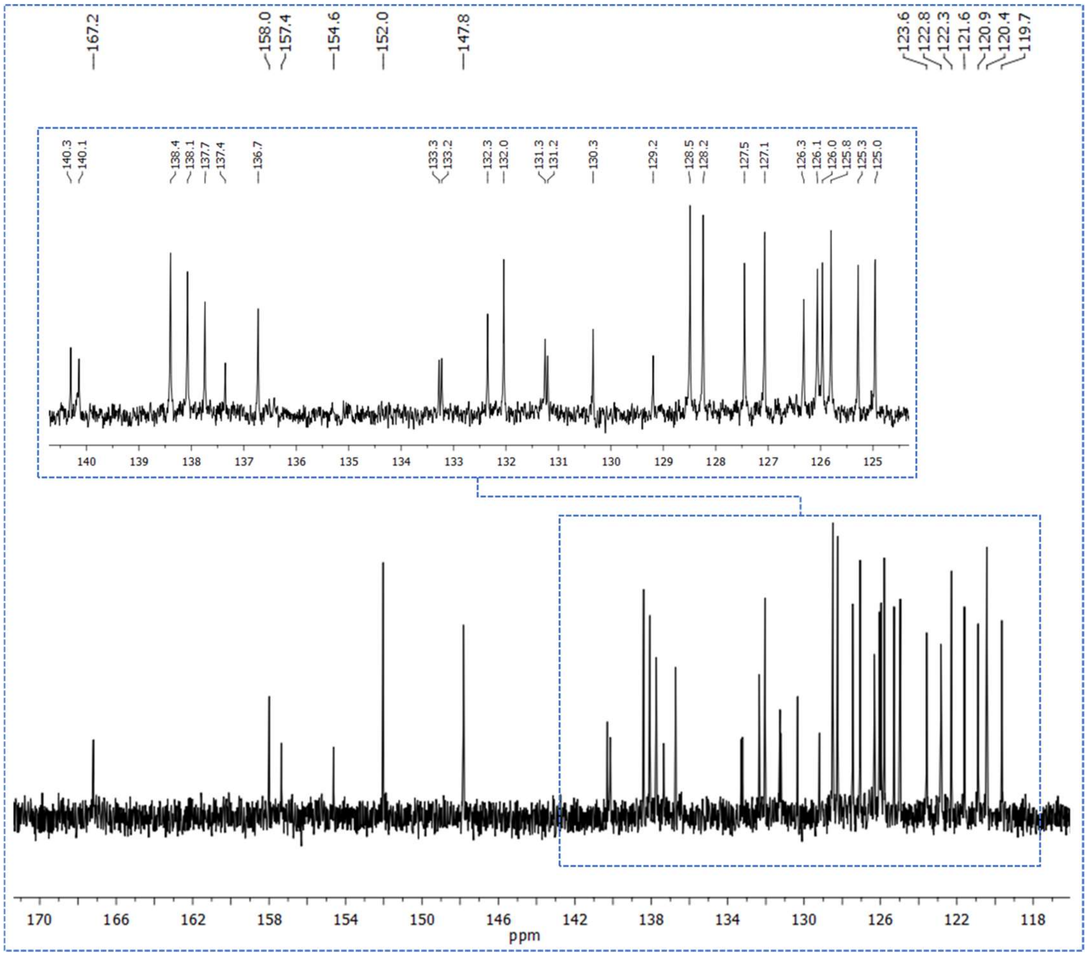

Figure S56. Zoomed-in $^{13}C\{^1H\}$ NMR spectrum (151 MHz, DCM-d_2) of **3c'**.

SCXRD analyses

Complex 2a (mm579)

Crystal Data and Experimental

Experimental. Orange block-shaped crystals of **2a** were obtained from a mixture of **2a** in DCM and Et_2O by slow evaporation. A suitable crystal with dimensions 0.24 × 0.16 × 0.07 mm^3 was selected and mounted on a Bruker APEX-II CCD diffractometer. The crystal was kept at a steady T = 100(2) K during data collection. Data were measured using ω and ϕ scans of 0.5° per frame for 5 s using MoKα radiation (TRIUMPH monochromator, sealed X-ray tube, 45 kV, 30 mA). The total number of runs and images was based on the strategy calculation from the program APEX4. The maximum resolution that was achieved was Θ = 30.724° (0.83 Å). The structure was solved in the ShelXT 2018/2 [20] solution program using iterative methods and by using Olex2.1.5[21] as the graphical interface. The model was refined with XL[20] using full matrix least squares minimization on F^2.

Crystal Data. $C_{30}H_{21}ClN_2Pt$, M_r = 640.03, orthorhombic, $Pna2_1$ (# 33), a = 14.4996(19) Å, b = 14.069(2) Å, c = 22.580(3) Å, $a = b = \gamma = 90^{\circ}$, V = 4606.4(11) Å^3, T = 100(2) K, Z = 8, Z' = 2, μ(MoK$_a$) = 6.231, 102158 reflections measured, 14048 unique (R_{int} = 0.0343) which were used in all calculations. The final wR_2 was 0.0714 (all data) and R_1 was 0.0335 (I≥2 σ(I)).

Structure solution and refinement

The unit cell was refined using SAINT V8.40B[22] on 9069 reflections, 9% of the observed reflections. The final completeness is 99.90 % out to 30.724° in Θ.

SADABS-2016/2[23] was used for absorption correction. wR_2 (int) was 0.1216 before and 0.0440 after correction. The Ratio of minimum to maximum transmission is 0.6693. The λ/2 correction factor is not present. The absorption coefficient μ of this material is 6.231 mm^{-1} at this wavelength (λ = 0.71073 Å)

and the minimum and maximum transmissions are 0.499 and 0.647.

The structure was solved and the space group *Pna*2_1 (# 33) determined by the ShelXT 2018/2[24] structure solution program using dual methods and refined by full matrix least squares minimization on F^2 using version 2019/1 of XL.[20] All non-hydrogen atoms were refined anisotropically. Hydrogen atom positions were calculated geometrically and refined using the riding model.

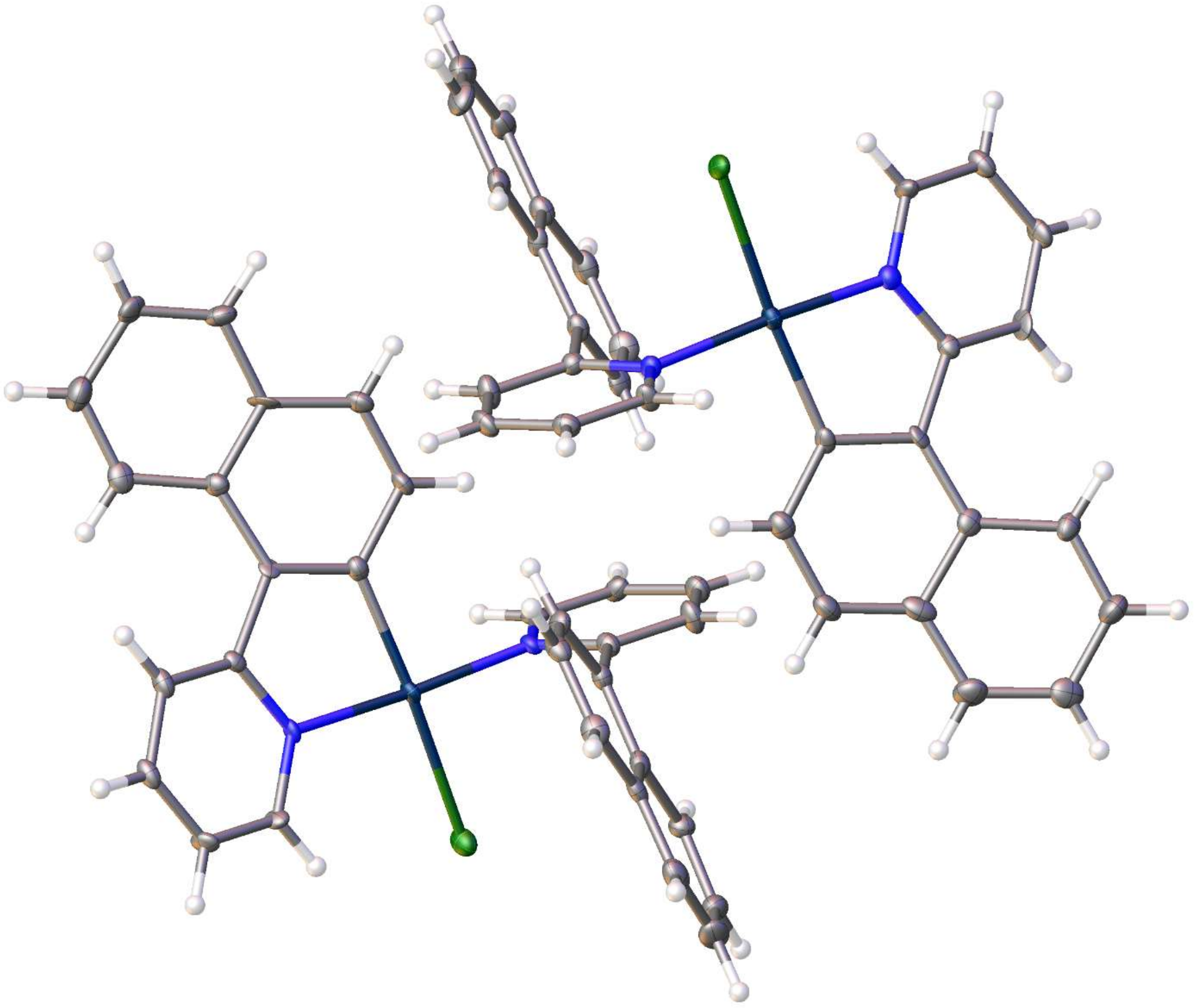

Figure S57. Molecular structure of **2a** as determined by SCXRD. Ellipsoids are plotted at 50% probability level.

Complex 2b (mm607)

Crystal Data and Experimental

Experimental. Single orange block-shaped crystals of **2b** were obtained by slow evaporation of a solution of **2b** in a mixture of DCM and Et_2O. A suitable crystal with dimensions 0.28 × 0.04 × 0.03 mm^3 was selected and mounted on a Bruker APEX-II CCD diffractometer. The crystal was kept at a steady T = 100(2) K during data collection. Data were measured using ω and ϕ scans of 0.5 ° per frame for 10 s using MoKα radiation (TRIUMPH monochromator, sealed X-ray tube, 45 kV, 30 mA). The total number of runs and images was based on the strategy calculation from the program APEX4. The maximum resolution that was achieved was Θ = 22.487° (0.93 Å). The structure was solved with the ShelXT 2018/2 [20] solution program using iterative methods and by using Olex2.1.5[21] as the graphical interface. The model was refined with XL[20] using full matrix least squares minimization on F^2.

Crystal Data. $C_{31}H_{22}Cl_4N_2Pt$, M_r = 759.39, monoclinic, $P2_1/n$ (No. 14), a = 18.290(3) Å, b = 7.4428(10) Å, c = 21.746(3) Å, b = 114.056(4)°, $a = \gamma$ = 90°, V = 2703.2(7) $Å^3$, T = 100(2) K, Z = 4, Z' = 1, $\mu(MoK_a)$ = 5.611, 23667 reflections measured, 3642 unique (R_{int} = 0.0849) which were used in all calculations. The final wR_2 was 0.1682 (all data) and R_1 was 0.0782 (I≥2 σ(I)).

Structure solution and refinement

The unit cell was refined using SAINT V8.40B[22] on 9991 reflections, 42% of the observed reflections. The final completeness is 99.50 % out to 22.487° in Θ.

SADABS-2016/2[23] was used for absorption correction.wR_2(int) was 0.1012 before and 0.0741 after correction. The Ratio of minimum to maximum transmission is 0.7046. The λ/2 correction factor is not present.

The structure was solved and the space group $P2_1/n$ (# 14) determined by the ShelXT 2018/2[24] structure solution program using iterative methods and refined by full matrix least squares minimization on F^2 using version 2019/1 of XL.[20] All non-hydrogen atoms were refined anisotropically. Hydrogen atom positions were calculated geometrically and refined using the riding model.

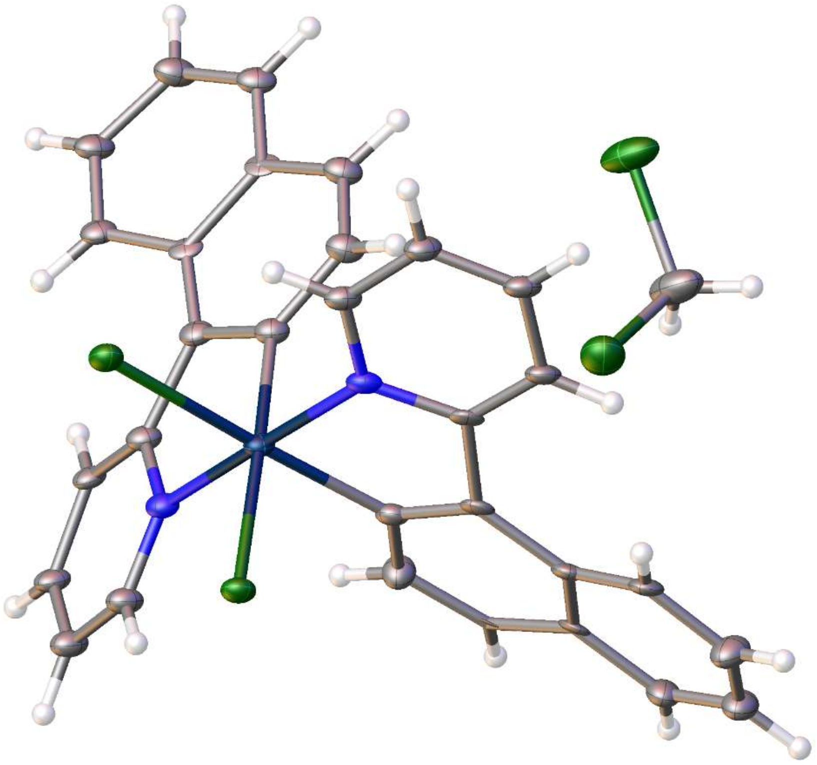

Figure S58. Molecular structure of **2b** as determined by SCXRD. Ellipsoids are plotted at 50% probability level.

Complex 2b' (mm567)

Crystal Data and Experimental

Experimental. Single orange block-shaped crystals of **2b'** were grown from a solution of **2b'** in a mixture of DCM and Et_2O by slow evaporation. A suitable crystal with dimensions 0.95 × 0.92 × 0.01 mm^3 was selected and mounted on a Bruker APEX-II CCD diffractometer. The

crystal was kept at a steady T = 100(2) K during data collection. Data were measured using ω and ϕ scans of 0.5 ° per frame for 30 s using MoKα radiation (TRIUMPH monochromator, sealed X-ray tube, 45 kV, 30 mA). The total number of runs and images was based on the strategy calculation from the program APEX4. The maximum resolution that was achieved was Θ = 25.408° (0.83 Å). The structure was solved with the ShelXT 2018/2[20] solution program using iterative methods and by using Olex2.1.5[21] as the graphical interface. The model was refined with XL[20] using full matrix least squares minimization on F^2.

Crystal Data. $C_{61}H_{42}Cl_4N_4Pt_2$, M_r = 716.93, triclinic, P-1 (# 2), a = 11.7578(7) Å, b = 11.7959(8) Å, c = 21.4886(13) Å, a = 104.299(2)°, b = 95.141(2)°, γ = 106.641(2)°, V = 2725.5(3) Å^3, T = 100(2) K, Z = 4, Z' = 2, μ(MoK$_a$) = 5.465, 9886 reflections measured, 9886 unique (R_{int} = 0.058) which were used in all calculations. The final wR_2 was 0.0994 (all data) and R_1 was 0.0475 (I≥2 σ(I)).

Structure solution and refinement

The unit cell was refined using SAINT V8.40B[22] on 6719 reflections, 68% of the observed reflections. The final completeness is 99.00 % out to 25.408° in Θ.

TWINABS-2012/1[25] was used for absorption correction. For component 1: wR_2(int) was 0.0670 before and 0.0479 after correction. For component 2: wR_2(int) was 0.0879 before and 0.0635 after correction. For component 3: wR_2(int) was 0.1115 before and 0.0870 after correction. The Ratio of minimum to maximum transmission is 0.77. Final HKLF 4 output contains 113570 reflections, R_{int} = 0.1039 (36409 with I > 3sig(I), R_{int} = 0.0580. The absorption coefficient μ of this material is 5.465 mm^{-1} at this wavelength (λ = 0.71073 Å) and the minimum and maximum transmissions are 0.729 and 0.947.

The structure was solved, and the space group P-1 (# 2) determined by the ShelXT 2018/2[24] structure solution program using iterative methods and refined by full matrix least squares minimization on F^2

using version 2019/1 of XL.[20] All non-hydrogen atoms were refined anisotropically. Hydrogen atom positions were calculated geometrically and refined using the riding model.

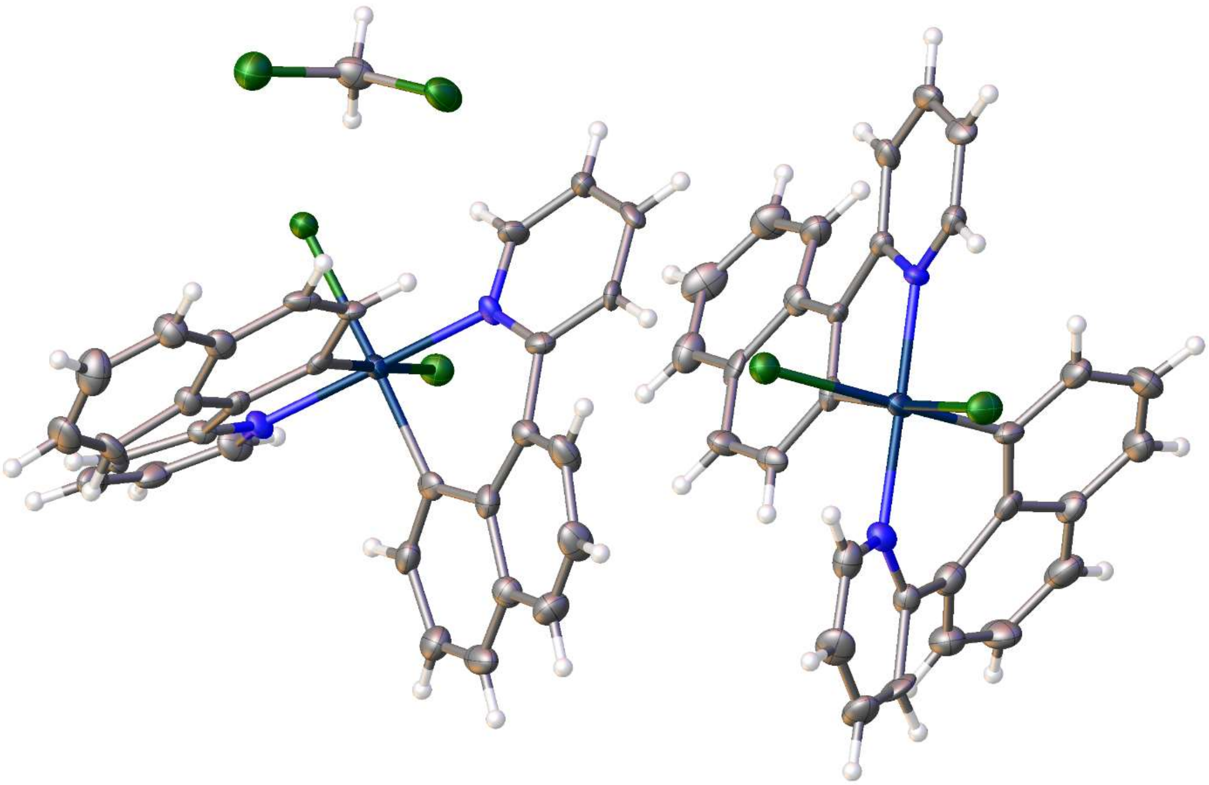

Figure S59. Molecular structure of **2b'** as determined by SCXRD. Ellipsoids are plotted at 50% probability level.

Complex 2c (mm585)

Crystal Data and Experimental

Experimental. Single colourless plate-shaped crystals of **2c** were obtained by slow evaporation of a solution of **2c** in a mixture of DCM and Et_2O. A suitable crystal with dimensions 0.43 × 0.38 × 0.06 mm^3 was selected and mounted on a Bruker APEX-II CCD diffractometer. The crystal was kept at a steady T = 100(2) K during data collection. Data were measured using ω and ϕ scans of 0.5 ° per frame for 5 s using MoKα radiation (TRIUMPH monochromator, sealed X-ray tube, 45 kV, 30 mA). The total number of runs and images was based on the strategy calculation from the program APEX4. The

maximum resolution that was achieved was Θ = 30.565° (0.83 Å). The structure was solved with the ShelXT 2018/2[20] solution program using iterative methods and by using Olex2.1.5[21] as the graphical interface. The model was refined with XL[20] using full matrix least squares minimization on F^2.

Crystal Data. $C_{30}H_{20}N_2Pt$, M_r = 603.57, monoclinic, $P2_1/n$ (# 14), a = 16.2246(7) Å, b = 7.6549(3) Å, c = 18.0393(8) Å, b = 109.5030(10)°, $a = \gamma$ = 90°, V = 2111.89(16) Å^3, T = 100.0 K, Z = 4, Z' = 1, $\mu(MoK_a)$ = 6.666, 34621 reflections measured, 6438 unique (R_{int} = 0.0187) which were used in all calculations. The final wR_2 was 0.0311 (all data) and R_1 was 0.0136 (I≥2 σ(I)).

Structure solution and refinement

The unit cell was refined using SAINT V8.40B[22] on 9925 reflections, 29% of the observed reflections. The final completeness is 99.60% out to 30.565° in Θ.

SADABS-2016/2[23] was used for absorption correction. wR_2(int) was 0.0580 before and 0.0286 after correction. The Ratio of minimum to maximum transmission is 0.7579. The λ/2 correction factor is not present. The absorption coefficient μ of this material is 6.666 mm^{-1} at this wavelength (λ = 0.71073 Å) and the minimum and maximum transmissions are 0.508 and 0.670.

The structure was solved, and the space group $P2_1/n$ (# 14) determined by the ShelXT 2018/2[24] structure solution program using iterative methods and refined by full matrix least squares minimization on F^2 using version 2019/1 of XL.[20] All non-hydrogen atoms were refined anisotropically. Hydrogen atom positions were calculated geometrically and refined using the riding model.

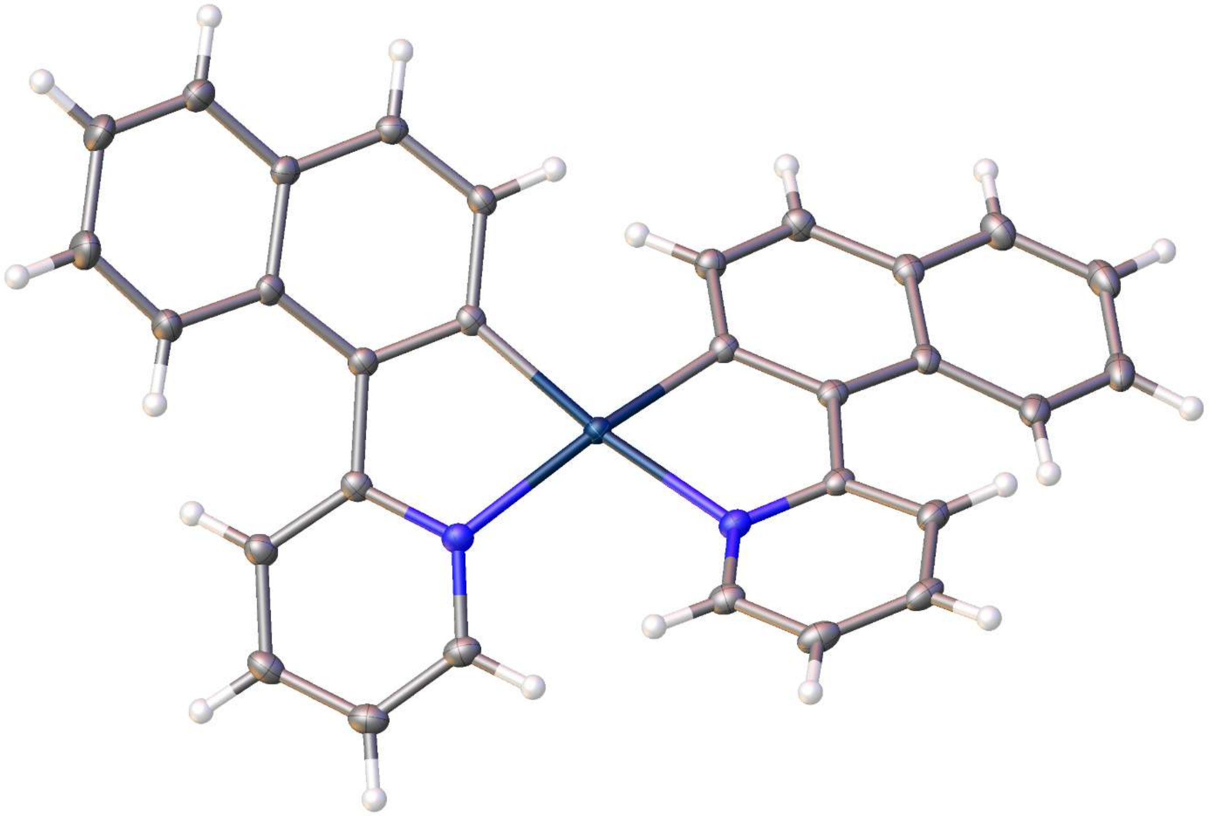

Figure S60. Molecular structure of **2c** as determined by SCXRD. Ellipsoids are plotted at 50% probability level.

Complex 3a (mm587)

Crystal Data and Experimental

Experimental. Single red block-shaped crystals of **3a** were obtained from slow evaporation of a solution of **3a** in a mixture of DCM and Et_2O. A suitable crystal with dimensions 0.38 × 0.32 × 0.25 mm^3 was selected and mounted on a Bruker APEX-II CCD diffractometer. The crystal was kept at a steady T = 100(2) K during data collection. Data were measured using ω and ϕ scans of 0.5° per frame for 30 s using MoKα radiation (TRIUMPH monochromator, sealed X-ray tube, 45 kV, 30 mA). The total number of runs and images was based on the strategy calculation from the program APEX4. The maximum resolution that was achieved was Θ = 25.505° (0.83 Å). The structure was solved with the

ShelXT 2018/2[20] solution program using iterative methods and by using Olex2.1.5[21] as the graphical interface. The model was refined with XL[20] using full matrix least squares minimization on F^2.

Crystal Data. $C_{77}H_{52}Cl_4N_4Pt_2$, M_r = 1565.20, triclinic, *P*-1 (# 2), a = 12.775(3) Å, b = 15.586(3) Å, c = 16.270(3) Å, *a* = 80.190(6)°, *b* = 76.548(6)°, *γ* = 85.221(6)°, *V* = 3101.4(11) Å^3, *T* = 100(2) K, *Z* = 2, *Z'* = 1, *μ*(MoK$_a$) = 4.727, 79093 reflections measured, 11460 unique (R_{int} = 0.0936) which were used in all calculations. The final wR_2 was 0.2045 (all data) and R_1 was 0.0684 (I≥2 *σ*(I)).

Structure solution and refinement

The unit cell was refined using SAINT V8.40B[22] on 9901 reflections, 13% of the observed reflections. The final completeness is 99.90% out to 25.505° in *Θ*.

No absorption correction was performed. The absorption coefficient *μ* of this material is 4.727 mm^{-1} at this wavelength (*λ* = 0.71073 Å) and the minimum and maximum transmissions are 0.217 and 0.307. The structure was solved, and the space group *P*-1 (# 2) determined by the ShelXT 2018/2[20] structure solution program using dual methods and refined by full matrix least squares minimization on F^2 using version 2019/3 of XL.[20] All non-hydrogen atoms were refined anisotropically. Hydrogen atom positions were calculated geometrically and refined using the riding model.

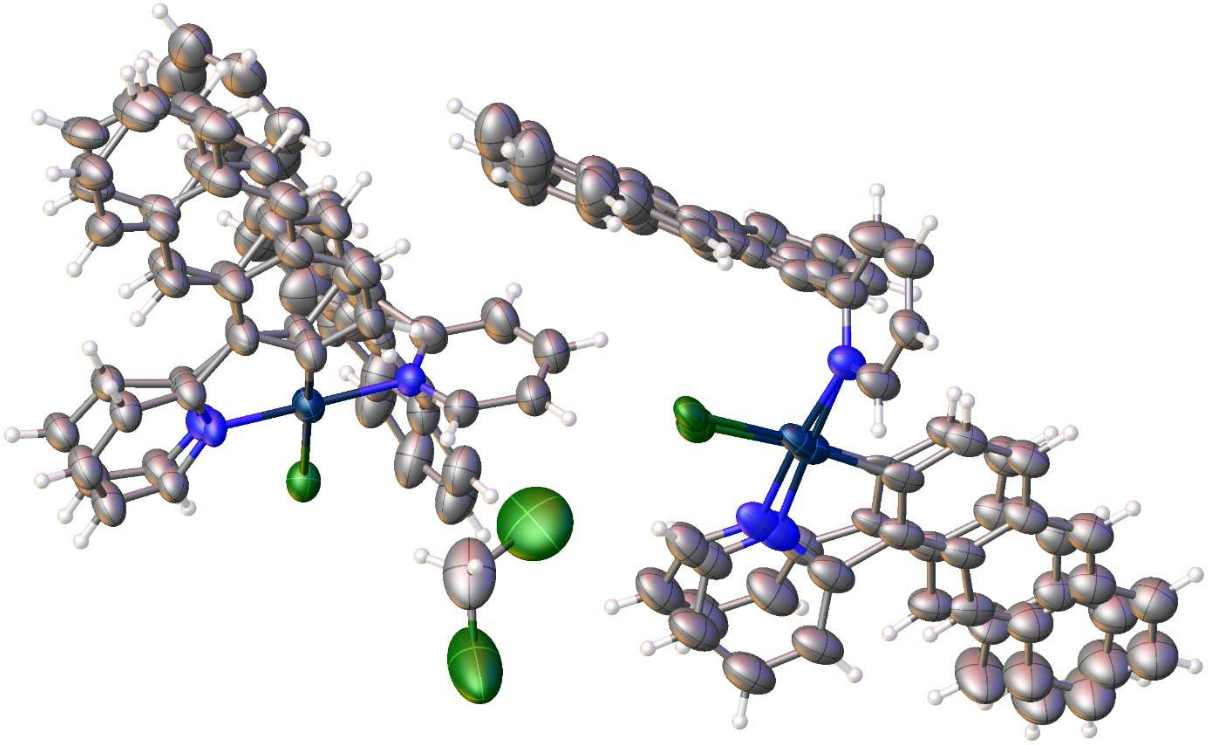

Figure S61. Molecular structure of **3a** as determined by SCXRD. Ellipsoids are plotted at 50% probability level.

Complex 3b (mm589)

Crystal Data and Experimental

Experimental. Single orange needle-shaped crystals of **3b** were recrystallized from a mixture of DCM, hexane and acetone by slow evaporation. A suitable crystal with dimensions 0.43 × 0.07 × 0.06 mm^3 was selected and mounted on a Bruker APEX-II CCD diffractometer. The crystal was kept at a steady T = 100(2) K during data collection. Data were measured using ω and ϕ scans of 0.5 ° per frame for 30 s using MoKα radiation (TRIUMPH monochromator, sealed X-ray tube, 45 kV, 30 mA). The total number of runs and images was based on the strategy calculation from the program APEX4. The maximum resolution that was achieved was Θ = 30.565° (0.83 Å). The structure was solved with the

ShelXT 2018/2[20] solution program using iterative methods and by using Olex2.1.5[21] as the graphical interface. The model was refined with XL[20] using full matrix least squares minimization on F^2.

Crystal Data. $C_{38}H_{24}Cl_2N_2Pt$, M_r = 774.58, orthorhombic, *Pbcn* (# 60), a = 14.9481(7) Å, b = 8.4291(4) Å, c = 21.4418(11) Å, $a = b = \gamma = 90^{\circ}$, V = 2701.6(2) Å^3, T = 100(2) K, Z = 4, Z' = 0.5, $\mu(MoK_a)$ = 5.426, 27983 reflections measured, 4115 unique (R_{int} = 0.0342) which were used in all calculations. The final wR_2 was 0.0876 (all data) and R_1 was 0.0345 ($I \geq 2\ \sigma(I)$).

Structure solution and refinement

The unit cell was refined using SAINT V8.40B[14] on 9869 reflections, 35% of the observed reflections. The final completeness is 99.80% out to 30.565° in Θ.

SADABS-2016/2[23] was used for absorption correction. wR_2(int) was 0.0966 before and 0.0451 after correction. The Ratio of minimum to maximum transmission is 0.6968. The λ/2 correction factor is not present. The absorption coefficient μ of this material is 5.426 mm^{-1} at this wavelength (λ = 0.71073 Å) and the minimum and maximum transmissions are 0.503 and 0.722.

The structure was solved and the space group *Pbcn* (# 60) determined by the XT[20] structure solution program using dual methods and refined by full matrix least squares minimization on F^2 using version 2019/1 of XL.[20] All non-hydrogen atoms were refined anisotropically. Hydrogen atom positions were calculated geometrically and refined using the riding model.

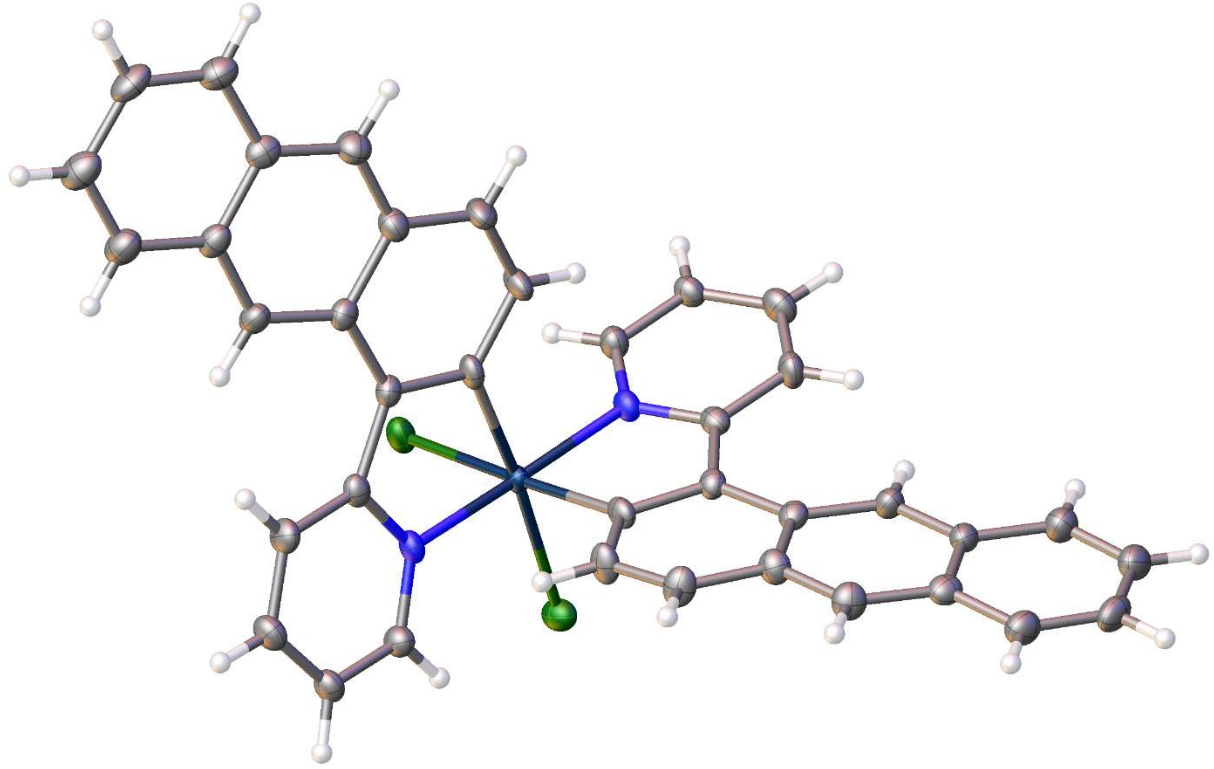

Figure S62. Molecular structure of **3b** as determined by SCXRD. Ellipsoids are plotted at 50% probability level.

Complex 3b' (mm574)

Crystal Data and Experimental

Experimental. Red needle-shaped crystals of **3b'** were recrystallized from a mixture of DCM and Et_2O by slow evaporation. A suitable crystal with dimensions 0.55 × 0.06 × 0.04 mm^3 was selected and mounted on a Bruker APEX-II CCD diffractometer. The crystal was kept at a steady T = 100(2) K during data collection. Data were measured using ω and ϕ scans of 0.5° per frame for 40 s using MoKα radiation (TRIUMPH monochromator, sealed X-ray tube, 45 kV, 30 mA). The total number of runs and images was based on the strategy calculation from the program APEX4. The maximum resolution that was achieved was Θ = 28.340° (0.83 Å). The structure was solved with the ShelXT 2018/2[20] solution program using iterative methods and by using Olex2.1.5[21] as the graphical interface. The model was

refined with XL[20] using full matrix least squares minimization on F^2.

Crystal Data. $C_{38.33}H_{24.66}Cl_{2.66}N_2Pt$, M_r = 802.71, monoclinic, $P2_1/c$ (# 14), a = 21.839(3) Å, b = 19.399(3) Å, c = 16.667(2) Å, b = 99.571(2)˚, $a = \gamma$ = 90˚, V = 6962.8(17) Å^3, T = 100(2) K, Z = 8, Z' = 2, μ(MoK$_a$) = 4.263, 105825 reflections measured, 17243 unique (R_{int} = 0.0580) which were used in all calculations. The final wR_2 was 0.1945 (all data) and R_1 was 0.0831 (I≥2 σ(I)).

Structure solution and refinement

The unit cell was refined using SAINT V8.40B[22] on 9941 reflections, 9% of the observed reflections. The final completeness is 99.80% out to 28.340˚ in Θ.

SADABS-2016/2[23] was used for absorption correction. wR_2(int) was 0.0650 before and 0.0485 after correction. The Ratio of minimum to maximum transmission is 0.6980. The λ/2 correction factor is not present. The absorption coefficient m of this material is 4.263 mm^{-1} at this wavelength (λ = 0.71073 Å) and the minimum and maximum transmissions are 0.589 and 0.843.

The structure was solved, and the space group $P2_1/c$ (# 14) determined by the XT 2018/2[20] structure solution program using dual methods and refined by full matrix least squares minimization on F^2 using version 2019/1 of XL.[20] All non-hydrogen atoms were refined anisotropically. Hydrogen atom positions were calculated geometrically and refined using the riding model.

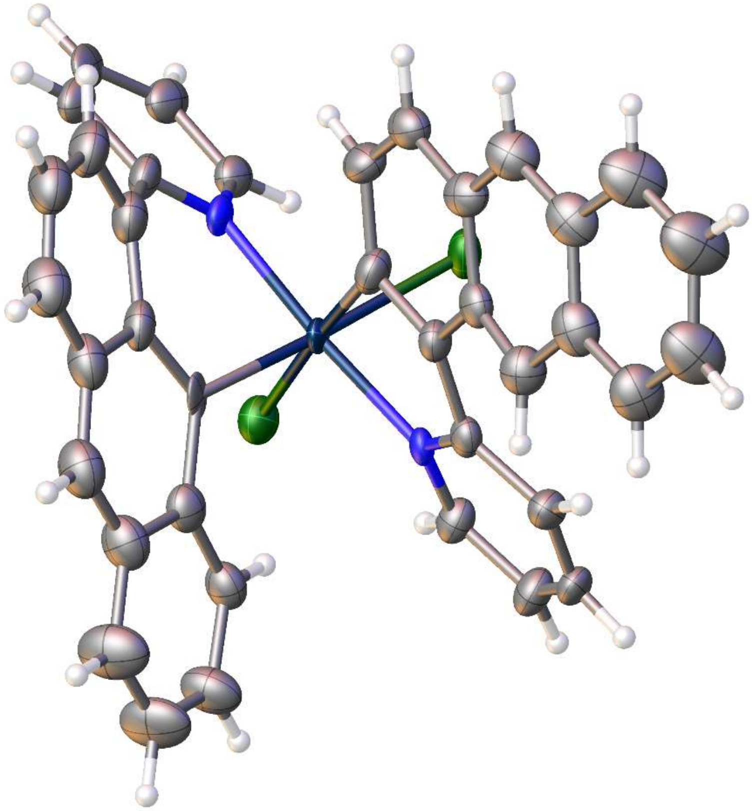

Figure S63. Molecular structure of **3b'** as determined by SCXRD. Ellipsoids are plotted at 50% probability level.

Complex 3c (mm582)

Crystal Data and Experimental

Experimental. Single colourless plate-shaped crystals of **3c** were recrystallized from a mixture of DCM and Et_2O by slow evaporation. A suitable crystal with dimensions 0.43 × 0.38 × 0.06 mm^3 was selected and mounted on a Bruker APEX-II CCD diffractometer. The crystal was kept at a steady T = 100(2) K during data collection. Data were measured using ω and ϕ scans of 0.5 ° per frame for 15 s using MoKα radiation (TRIUMPH monochromator, sealed X-ray tube, 45 kV, 30 mA). The total number of runs and images was based on the strategy calculation from the program APEX4. The maximum

resolution that was achieved was Θ = 28.102° (0.83 Å). The structure was solved with the ShelXT 2018/2[20] solution program using iterative methods and by using Olex2.1.5[21] as the graphical interface. The model was refined with XL[20] using full matrix least squares minimization on F^2.

Crystal Data. $C_{38}H_{24}N_2Pt$, M_r = 703.68, monoclinic, $P2_1/c$ (# 14), a = 17.067(3) Å, b = 7.9171(12) Å, c = 19.976(3) Å, b = 101.319(4)°, $a = \gamma$ = 90°, V = 2646.6(7) Å^3, T = 296.15 K, Z = 4, Z' = 1, $\mu(MoK_a)$ = 5.334, 41827 reflections measured, 6347 unique (R_{int} = 0.0780) which were used in all calculations. The final wR_2 was 0.1211 (all data) and R_1 was 0.0509 ($I \geq 2\ \sigma(I)$).

Structure solution and refinement

The unit cell was refined using SAINT V8.40B[22] on 9849 reflections, 24% of the observed reflections. The final completeness is 99.60% out to 28.102° in Θ.

SADABS-2016/2[23] was used for absorption correction. wR_2(int) was 0.1292 before and 0.0763 after correction. The Ratio of minimum to maximum transmission is 0.4281. The $\lambda/2$ correction factor is not present. The absorption coefficient μ of this material is 5.334 mm^{-1} at this wavelength (λ = 0.71073 Å) and the minimum and maximum transmissions are 0.365 and 0.852.

The structure was solved, and the space group $P2_1/c$ (# 14) determined by the XT 2018/2[20] structure solution program using iterative methods and refined by full matrix least squares minimization on F^2 using version 2019/1 of ShelXL.[20] All non-hydrogen atoms were refined anisotropically. Hydrogen atom positions were calculated geometrically and refined using the riding model.

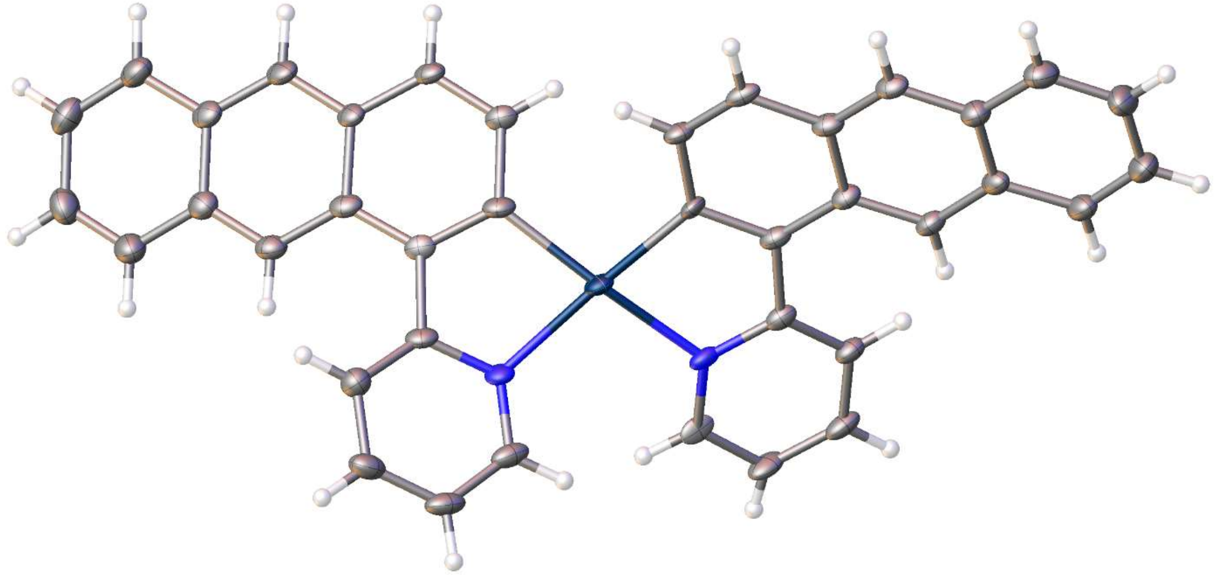

Figure S64. Molecular structure of **3c** as determined by SCXRD. Ellipsoids are plotted at 50% probability level.

Table S7. Crystallograhic structure refinement data and for all Pt complexes.

	**2a (mm579)**	**2b (mm607)**	**2b' (mm567)**	**2c (mm585)**
Chemical formula	$C_{30}H_{21}ClN_2Pt$	$C_{31}H_{22}Cl_4N_2Pt_2$	$C_{30.5}H_{21}Cl_3N_2Pt$	$C_{30}H_{20}N_2Pt$
Fomula weight	640.03	759.39	716.93	603.57
Crystal size (mm)	0.24 × 0.16 × 0.07	0.28 × 0.04 × 0.03	0.95 × 0.92 × 0.01	0.43 × 0.38 × 0.06
Temperature (K)	100(2)	100(2)	100(2)	100(2)
Crystal system	Orthorhombic	Monoclinic	Triclinic	Monoclinic
Space group	$Pna2_1$	$P2_1/c$	P-1	$P2_1/c$
Unit cell dimensions	a = 14.4996(19) Å<br>b = 14.069(2) Å<br>c = 22.580(3) Å<br>a = b = γ = 90°	a = 18.290(3) Å<br>b = 7.4428(10) Å<br>c = 21.746(3) Å<br>a = $γ$ = 90°<br>$β$ = 114.056(4)°	a = 11.7578(7) Å<br>b = 11.7959(8) Å<br>c = 21.4886(13) Å<br>a = 104.299(2)°<br>$β$ = 95.141(2)°<br>γ = 106.641(2)°	a = 16.2246(7) Å<br>b = 7.6549(3) Å<br>c = 18.0393(8) Å<br>a = $γ$ = 90°<br>$β$ = 109.5030(10)°
Unit Cell Volume($Å^3$)	4606.4(11)	2703.2(7)	2725.5(3)	2111.89(16)
Z	8	4	4	4
Absorption coefficient (mm^{-1})	6.231	5.611	5.465	6.666
Total reflections collected	102158	23667	9886	34621
Unique reflections	14048 [R_{int} = 0.0343]	3642 [R_{int} = 0.0849]	9886 [R_{int} = 0.058]	6438 [R_{int} = 0.0187]
Completeness to theta	30.724°<br>99.90%	22.487°<br>99.50%	25.408°<br>99.00%	30.565°<br>99.60%
Goodness-of-fit on F^2	1.241	1.298	1.056	1.058
Final R_1 value [I>2σ(I)]	0.0335	0.0782	0.0475	0.0136

Final wR_2 value (all data)	0.0714	0.1682	0.0994	0.0311

	**3a (mm587)**	**3b (mm589)**	**3b' (mm574)**	**3c' (mm582)**
Chemical formula	$C_{77}H_{52}Cl_4N_4Pt_2$	$C_{38}H_{24}Cl_2N_2Pt$	$C_{38.33}H_{24.66}Cl_{2.66}N_2Pt$	$C_{38}H_{24}N_2Pt$
Fomula weight	1565.2	774.58	802.73	703.68
Crystal size (mm)	0.38 × 0.32 × 0.25	0.43 × 0.07 × 0.06	0.55 × 0.06 × 0.04	0.32 × 0.06 × 0.03
Temperature (K)	100(2)	100(2)	100(2)	100(2)
Crystal system	Triclinic	Orthorhombic	Monoclinic	Monoclinic
Space group	*P*-1	*Pbcn*	$P2_1/c$	$P2_1/c$
Unit cell dimensions	*a* = 12.775(3) Å<br>*b* = 15.586(3) Å<br>*c* = 16.270(3) Å<br>*a* = 80.190(6)°<br>*β* = 76.548(6)°<br>γ = 85.221(6)°	a = 14.9481(7) Å<br>b = 8.4291(4) Å<br>c = 21.4418(11) Å<br>a = b = γ = 90°	*a* = 21.839(3) Å<br>*b* = 19.399(3) Å<br>*c* = 16.667(2) Å<br>*a* = *γ* = 90°<br>*β* = 99.571(2)°	*a* = 17.067(3) Å<br>*b* = 7.9171(12) Å<br>*c* = 19.976(3) Å<br>*a* = *γ* = 90°<br>*β* = 101.319(4)°
Unit Cell Volume($Å^3$)	3101.4(11)	2701.6(2)	6962.8(17)	2646.6(7)
Z	2	4	8	4
Absorption coefficient (mm^{-1})	4.727	5.426	4.263	5.334
Total reflections collected	79093	27983	105825	41827
Unique reflections	11460 [R_{int} = 0.0936]	4115 [R_{int} = 0.0342]	17243 [R_{int} = 0.0580]	6347 [R_{int} = 0.0780]
Completeness to theta	25.505°<br>99.90%	30.565°<br>99.80%	38.340°<br>99.80%	28.102°<br>99.60%
Goodness-of-fit on F^2	1.037	1.092	1.048	1.159
Final R_1 value [$I>2\sigma(I)$]	0.0684	0.0345	0.0831	0.0509
Final wR_2 value (all data)	0.2045	0.0876	0.1945	0.1211

References

1 X. Yu, J. Tang, X. Jin, Y. Yamamoto and M. Bao, *Asian J. Org. Chem.*, 2018, **7**, 550–553.

2 J.-Y. Cho, K. Yu. Suponitsky, J. Li, T. V. Timofeeva, S. Barlow and S. R. Marder, *J. Organomet. Chem.*, 2005, **690**, 4090–4093.

3 C. P. Newman, K. Casey-Green, G. J. Clarkson, G. W. V. Cave, W. Errington and J. P. Rourke, *Dalton Trans.*, 2007, 3170–3182.

4 I. Allison, H. Lim, A. Shukla, V. Ahmad, M. Hasan, K. Deshmukh, R. Wawrzinek, S. K. M. McGregor, J. K. Clegg, V. V. Divya, C. Govind, C. H. Suresh, V. Karunakaran, N. U. K. N., A. Ajayaghosh, E. B. Namdas and S.-C. Lo, *ACS Appl. Electron. Mater.*, 2019, **1**, 1304–1313.

5 A. M. Brouwer, *Pure Appl. Chem.*, 2011, **83**, 2213–2228.

6 M. J. Frisch, G. W. Trucks, H. B. Schlegel, G. E. Scuseria, M. A. Robb, J. R. Cheeseman, G. Scalmani, V. Barone, G. A. Petersson, H. Nakatsuji, X. Li, M. Caricato, A. V. Marenich, J. Bloino, B. G. Janesko, R. Gomperts, B. Mennucci, H. P. Hratchian, J. V. Ortiz, A. F. Izmaylov, J. L. Sonnenberg, D. Williams-Young, F. Ding, F. Lipparini, F. Egidi, J. Goings, B. Peng, A. Petrone, T. Henderson, D. Ranasinghe, V. G. Zakrzewski, J. Gao, N. Rega, G. Zheng, W. Liang, M. Hada, M. Ehara, K. Toyota, R. Fukuda, J. Hasegawa, M. Ishida, T. Nakajima, Y. Honda, O. Kitao, H. Nakai, T. Vreven, K. Throssell, J. A. Montgomery Jr., J. E. Peralta, F. Ogliaro, M. J. Bearpark, J. J. Heyd, E. N. Brothers, K. N. Kudin, V. N. Staroverov, T. A. Keith, R. Kobayashi, J. Normand, K. Raghavachari,

A. P. Rendell, J. C. Burant, S. S. Iyengar, J. Tomasi, M. Cossi, J. M. Millam, M. Klene, C. Adamo, R. Cammi, J. W. Ochterski, R. L. Martin, K. Morokuma, O. Farkas, J. B. Foresman and D. J. Fox, 2016.

7 J.-D. Chai and M. Head-Gordon, *J. Chem. Phys.*, 2008, **128**, 084106.

8 Y. Zhao and D. G. Truhlar, *Theor. Chem. Acc.*, 2008, **120**, 215–241.

9 S. Grimme, S. Ehrlich and L. Goerigk, *J. Comput. Chem.*, 2011, **32**, 1456–1465.

10 M. M. Francl, W. J. Pietro, W. J. Hehre, J. S. Binkley, M. S. Gordon, D. J. DeFrees and J. A. Pople, *J. Chem. Phys.*, 1982, **77**, 3654–3665.

11 T. H. Dunning Jr., *J. Chem. Phys.*, 1989, **90**, 1007–1023.

12 D. Figgen, K. A. Peterson, M. Dolg and H. Stoll, *J. Chem. Phys.*, 2009, **130**, 164108.

13 G. Scalmani and M. J. Frisch, *J. Chem. Phys.*, 2010, **132**, 114110.

14 Y. Wu, G. Wang, Q. Li, J. Xiang, H. Jiang and Y. Wang, *Nat. Commun.*, 2018, **9**, 1953.

15 J. Wu, B. Xu, Y. Xu, L. Yue, J. Chen, G. Xie and J. Zhao, *Inorg. Chem.*, 2023, **62**, 19142–19152.

16 F. Juliá, M.-D. García-Legaz, D. Bautista and P. González-Herrero, *Inorg. Chem.*, 2016, **55**, 7647–7660.

17 R. E. N. Njogu, P. Fodran, Y. Tian, L. W. Njenga, D. K. Kariuki, A. O. Yusuf, I. Scheblykin, O. F. Wendt and C.-J. Wallentin, *Synlett*, 2019, **30**, 792–798.

18 N. P. Tsvetkov, E. Gonzalez-Rodriguez, A. Hughes, G. dos Passos Gomes, F. D. White, F. Kuriakose and I. V. Alabugin, *Angew. Chem. Int. Ed.*, 2018, **57**, 3651–3655.

19 X. Wang, W.-G. Liu, L.-T. Liu, X.-D. Yang, S. Niu, C.-H. Tung, L.-Z. Wu and H. Cong, *Org. Lett.*, 2021, **23**, 5485–5490.

20 G. M. Sheldrick, *Acta Crystallogr. Sect. Found. Adv.*, 2015, **71**, 3–8.

21 O. V. Dolomanov, L. J. Bourhis, R. J. Gildea, J. a. K. Howard and H. Puschmann, *J. Appl. Crystallogr.*, 2009, **42**, 339–341.

22 Madison, WI, 2013.

23 SADABS-2016/2, Bruker AXS, Madison, WI., Sadabs (version 2016/2) Bruker 2016.

24 L. J. Bourhis, O. V. Dolomanov, R. J. Gildea, J. a. K. Howard and H. Puschmann, *Acta Crystallogr. Sect. Found. Adv.*, 2015, **71**, 59–75.

25 M. Sevvana, M. Ruf, I. Usón, G. M. Sheldrick and R. Herbst-Irmer, *Acta Crystallogr. Sect. Struct. Biol.*, 2019, **75**, 1040–1050.
